# Dioxygen Activation with Molybdenum Complexes Bearing Amide-Functionalized Iminophenolate Ligands

**DOI:** 10.3390/molecules24091814

**Published:** 2019-05-10

**Authors:** Niklas Zwettler, Madeleine A. Ehweiner, Jörg A. Schachner, Antoine Dupé, Ferdinand Belaj, Nadia C. Mösch-Zanetti

**Affiliations:** Institute of Chemistry, Inorganic Chemistry, University of Graz, Schubertstrasse 1, 8010 Graz, Austria; niklas.zwettler@uni-graz.at (N.Z.); madeleine.ehweiner@uni-graz.at (M.A.E.); joerg.schachner@uni-graz.at (J.A.S.); antoine.dupe@uni-graz.at (A.D.); ferdinand.belaj@uni-graz.at (F.B.)

**Keywords:** molybdenum, iminophenolate ligands, dioxygen activation, peroxido complexes

## Abstract

Two novel iminophenolate ligands with amidopropyl side chains (**HL2** and **HL3**) on the imine functionality have been synthesized in order to prepare dioxidomolybdenum(VI) complexes of the general structure [MoO_2_**L_2_**] featuring pendant internal hydrogen bond donors. For reasons of comparison, a previously published complex featuring *n*-butyl side chains (**L1**) was included in the investigation. Three complexes (**1**–**3**) obtained using these ligands (**HL1**–**HL3**) were able to activate dioxygen in an in situ approach: The intermediate molybdenum(IV) species [MoO(PMe_3_)**L_2_**] is first generated by treatment with an excess of PMe_3_. Subsequent reaction with dioxygen leads to oxido peroxido complexes of the structure [MoO(O_2_)**L_2_**]. For the complex employing the ligand with the *n*-butyl side chain, the isolation of the oxidomolybdenum(IV) phosphino complex [MoO(PMe_3_)(**L1**)_2_] (**4**) was successful, whereas the respective Mo(IV) species employing the ligands with the amidopropyl side chains were found to be not stable enough to be isolated. The three oxido peroxido complexes of the structure [MoO(O_2_)**L_2_**] (**9**–**11**) were systematically compared to assess the influence of internal hydrogen bonds on the geometry as well as the catalytic activity in aerobic oxidation. All complexes were characterized by spectroscopic means. Furthermore, molecular structures were determined by single-crystal X-ray diffraction analyses of **HL3**, **1**–**3**, **9**–**11** together with three polynuclear products {[MoO(**L2**)_2_]_2_(µ-O)} (**7**), {[MoO(**L2**)]_4_(µ-O)_6_} (**8**) and [C_9_H_13_N_2_O]_4_[Mo_8_O_26_]·6OPMe_3_ (**12**) which were obtained during the synthesis of reduced complexes of the type [MoO(PMe_3_)**L_2_**] (**4**–**6**).

## 1. Introduction

Molybdenum is an earth-abundant transition metal with crucial biological relevance for most organisms including humans [1,2,3]. The molybdenum(VI) dioxido motif, which is widespread in oxygen atom transfer (OAT) enzymes [1,4,5], sparked the intensive investigation of mononuclear dioxidomolybdenum(VI) complexes over the past decades, both in biomimetic chemistry as well as in oxygenation catalysis [2,6,7,8,9,10,11,12,13]. A major drawback in virtually all reported catalytic systems is, however, the necessity of a terminal oxidant [13,14,15,16,17,18,19,20,21]. Thus, the possibility to use environmentally benign and cheap dioxygen (or even air) would be highly desirable [22]. Several transition metals, including molybdenum, are known to activate dioxygen [23,24,25,26,27,28,29,30,31,32]. In our group, molybdenum(IV) complexes based on β-ketiminate and iminophenolate ligand systems were investigated regarding their usability in dioxygen activation [33,34,35,36]. As a result, we were able to demonstrate the catalytic aerobic oxidation of phosphanes and determine the influence of steric and electronic properties of the ligands. In this context, we reported the unusual electronic properties of the first imido supported molybdenum peroxido complex [35]. A common disadvantage of the investigated systems is, however, the rather high stability of molybdenum(VI) oxido and peroxido complexes, which is why we are currently investigating intra- and intermolecular additives to render these moieties more reactive. Very recently, we were able to increase the reactivity of a terminal molybdenum oxido group by the addition of a strong Lewis acid, leading to a reduction of the Mo=O bond with hydrosilanes [37]. 

**Figure 1:**
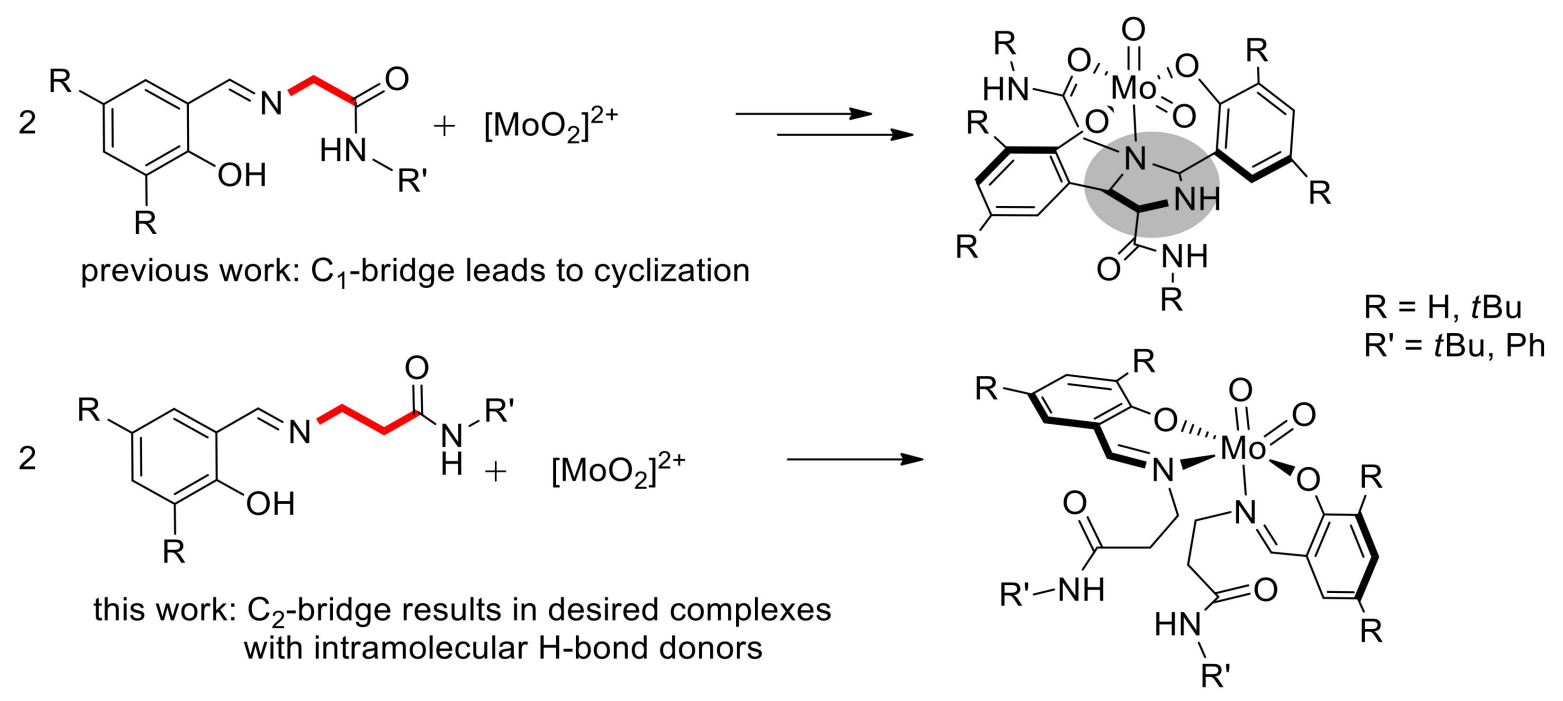
Iminophenolate ligands with amide functionalities featuring a C_1_ and C_2_ bridge, respectively, and their behavior upon coordination to [MoO_2_]^2+^.

Inspired by biomimetic modeling approaches focusing on the importance of the second coordination sphere, e.g., hydrogen bonding [23,24,38,39,40,41,42,43], we started to investigate the influence of internal hydrogen bond donors on dioxygen activation. Previous results demonstrated a beneficial effect of such side-arm functionalized iminophenolate ligands on catalytic activity and selectivity [16]. First attempts to introduce an amide side-arm into an iminophenolate ligand system unexpectedly resulted in stepwise C–C and C–N coupling upon coordination at the electron poor dioxidomolybdenum(VI) center (Figure 1) [44]. This unusual coupling behavior is presumably caused by the two electron withdrawing moieties adjacent to the methylene carbon. We thus reasoned that an elongation of the side chain by one methylene group should allow for the isolation of the desired complexes (Figure 1).

Herein, we report on the synthesis and coordination chemistry of novel iminophenolate ligands with amidopropyl functionalities, which were successfully coordinated to dioxidomolybdenum centers to form mononuclear complexes bearing pendant amide functionalities as hydrogen bond donors. The synthesis of such mononuclear complexes has not always been achieved since basic conditions during the synthetic procedure often result in deprotonation of the N–H leading to unidentified polymeric compounds [45]. The resulting compounds were investigated with regard to the reduction of the metal center and subsequent dioxygen activation. For comparison, a previously reported dioxidomolybdenum complex featuring *n*-butyl side chains was also investigated [16]. All three dioxidomolybdenum complexes were able to activate dioxygen in an in situ approach via molybdenum(IV) species. The formed oxido peroxido complexes were fully characterized including single-crystal X-ray diffraction analysis. The dioxido complexes were furthermore found to be active precatalysts in aerobic oxidation.

## 2. Results and Discussion

### 2.1. Ligand Synthesis

**Scheme 1:**
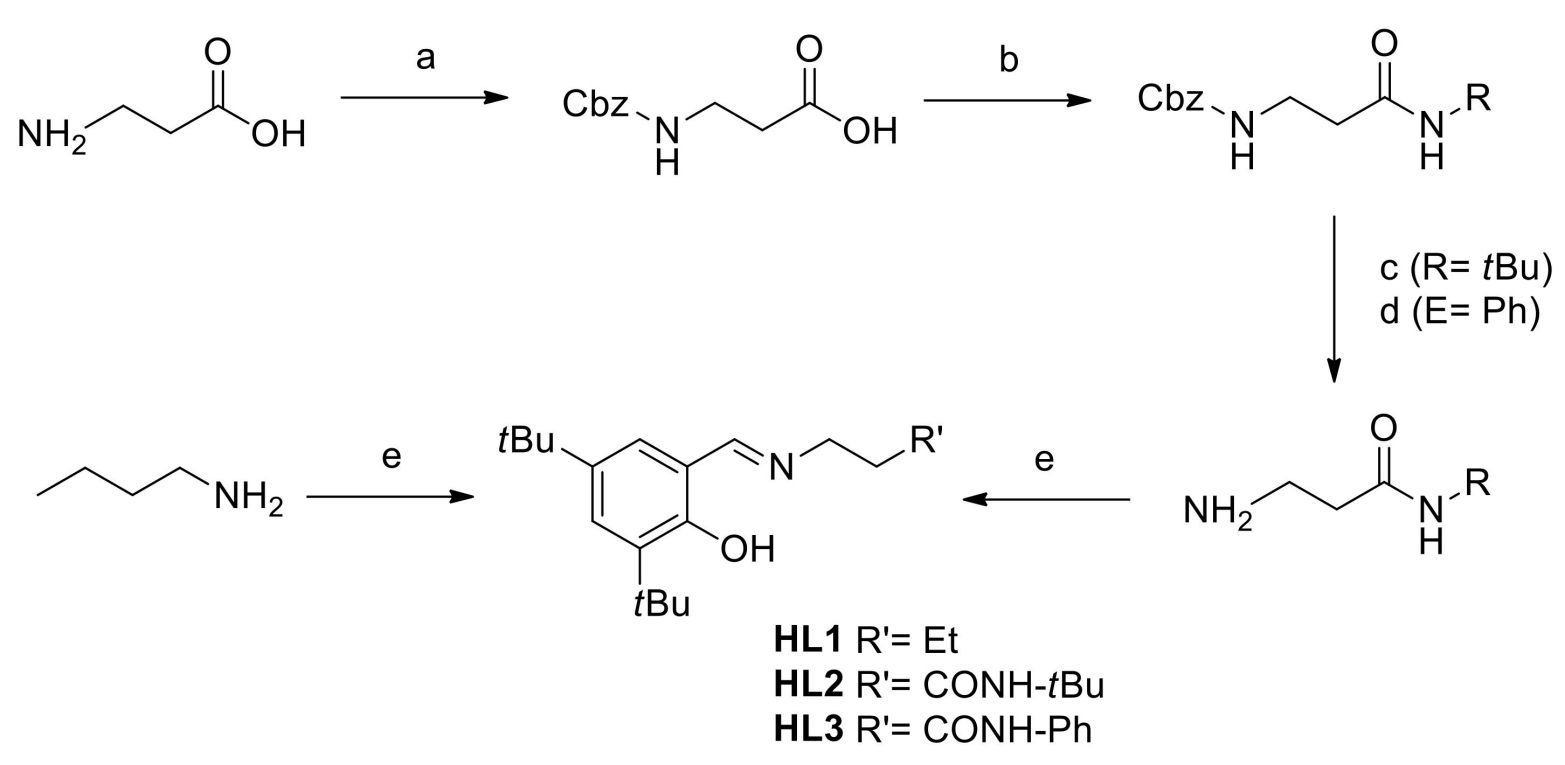
Synthesis of **HL1**–**HL3**; (a) benzyl chloroformate (1 equiv), NaOH 1 M, 0 °C to 25 °C, 16 h; (b) i. ethyl chloroformate (1 equiv), Et_3_N (1 equiv), toluene, −10 °C, 15 min; ii. R-NH_2_ (1 equiv in 10 mL CHCl_3_), toluene, −10 °C to 110 °C, 1 h; (c) Pd/C (15 w%), H_2_ (5 atm), MeOH, 25 °C, 16 h; (d) i. HBr (33% in AcOH, 20 mL), 25 °C, 16 h; ii. NaOH 2 M; (e) 3,5-di-*tert*-butyl-2-hydroxybenzaldehyde (1 equiv), MeOH, 60 °C, 16 h.

Ligand **HL1** featuring *n*-butyl side chains was synthesized according to a previously published procedure (Scheme 1) [46]. The novel amide-functionalized ligands **HL2** and **HL3** are accessible starting with commercially available β-alanine in a four-step procedure as shown in Scheme 1. In the first step, the amino group is protected with a carboxybenzyl (Cbz) group according to published procedures [47]. In the second step, the carboxylic acid moiety is chlorinated using ethyl chloroformate, and subsequent reaction with a primary amine yields the *tert*-butyl and phenyl amide derivatives, respectively. Deprotection with Pd/C, H_2_ (R = *t*Bu) or HBr (33% in acetic acid; R = Ph) gives the desired free aminoamides, which were subsequently condensed with 3,5-di-*tert*-butyl-2-hydroxybenzaldehyde to yield the desired Schiff base ligands **HL2** (R = *t*Bu, 56% overall yield) and **HL3** (R = Ph, 48% overall yield). 

Analytical data for the previously reported ligand **HL1** match those in literature [46]. All three ligands **HL1**–**HL3** were characterized by ^1^H- and ^13^C-NMR and IR spectroscopy as well as EI-MS; for **HL3**, the molecular structure was determined via single-crystal X-ray diffraction analysis. Similar to the related amidoethyl (C1) analogues [44], **HL2** and **HL3** (amidopropyl, C2) feature a significant difference in the shift of the amide NH proton depending on the R’ substituent (4.86 ppm in **HL2** and 6.89 ppm in **HL3**, *tert*-butyl amide vs. phenyl amide, C_6_D_6_). 

### 2.2. Synthesis of Dioxidomolybdenum(VI) Complexes

**Scheme 2:**
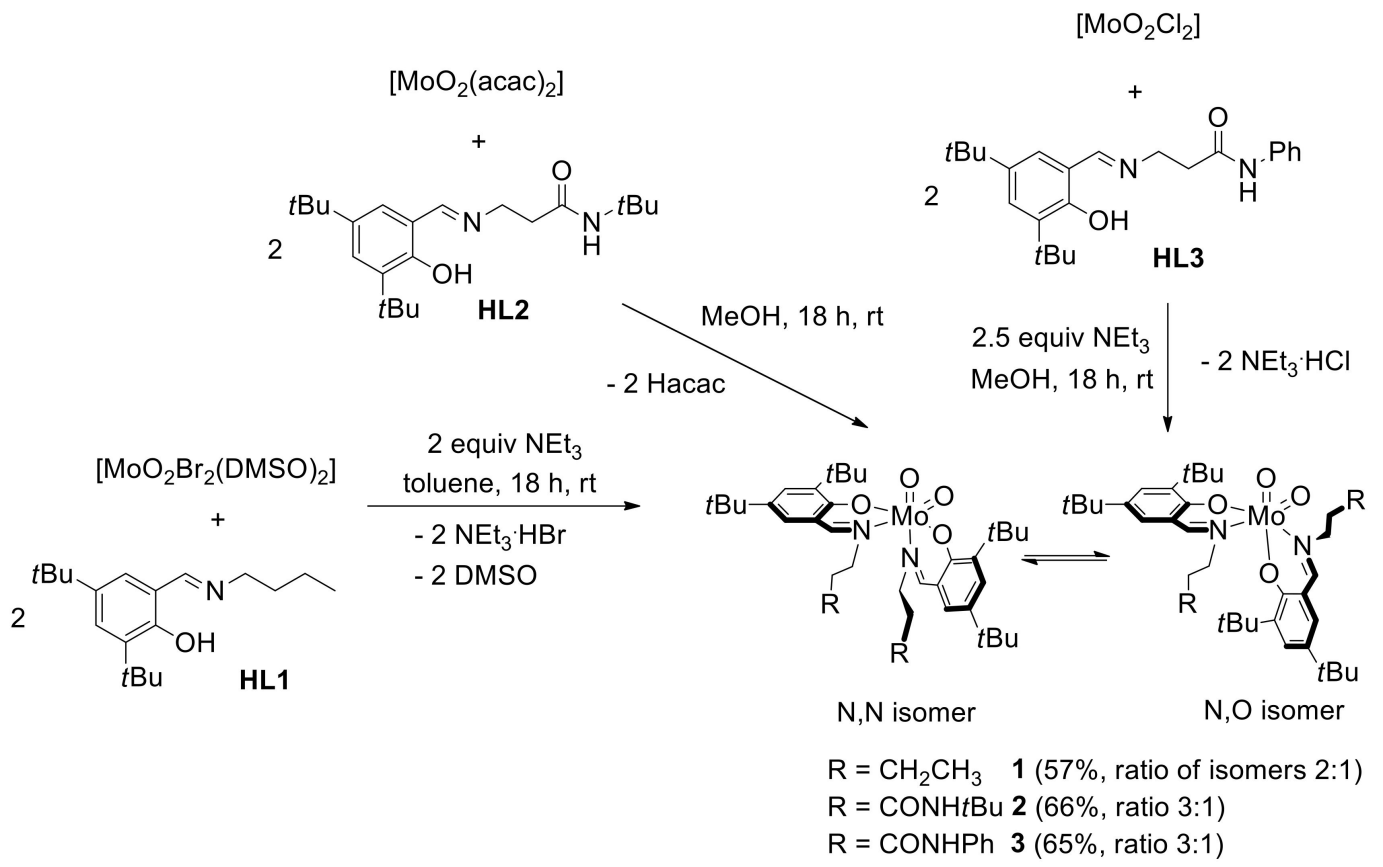
Synthesis of complexes **1**–**3**.

For the synthesis of molybdenum(VI) complexes of the type [MoO_2_**L_2_**], synthetic procedures using [MoO_2_(acac)_2_] [48], [MoO_2_Cl_2_] or [MoO_2_Br_2_(DMSO)_2_] [49] as metal source were investigated. Alternative to the literature [16], complex [MoO_2_(**L1**)_2_] (**1**) was synthesized via a reaction of two equiv of **HL1** with the metal precursor [MoO_2_Br_2_(DMSO)_2_] in the presence of excess NEt_3_ in good yield. Complex [MoO_2_(**L2**)_2_] (**2**) was synthesized following a similar procedure using [MoO_2_Cl_2_] and two equiv **HL2** in the presence of excess NEt_3_. The compound was also accessible in good yield by reacting two equiv of **HL2** with [MoO_2_(acac)_2_]. Complex [MoO_2_(**L3**)_2_] (**3**) was synthesized in good yield via reaction of [MoO_2_Cl_2_] with two equiv of **HL3**. Synthesis attempts starting from [MoO_2_(acac)_2_] resulted in the formation of the monosubstituted complex [MoO_2_(acac)(**L3**)] (**3^acac^**) instead. This statement is supported by ^1^H-NMR spectroscopy featuring resonances for one acetylacetonate and one iminophenolate ligand, as well as single-crystal X-ray diffraction analysis (vide infra). The formation of such a monosubstituted complex was previously observed with a phenyl-imine based ligand [36]. All complex syntheses are summarized in Scheme 2. 

Complexes **1**–**3** are in a dynamic equilibrium between a symmetric (*N*,*N* with respect to the oxido *trans* positions) and an asymmetric isomer (*N*,*O*) in solution, with approximate isomeric ratios of 2:1 (**1**, C_6_D_6_) and 3:1 (**2** and **3**, C_6_D_6_). The *N*,*N* isomer is electronically favored while large groups at *N* lead to the *O*,*O* isomer as observed in a system with a iminophenolate ligand with a *tert*-butyl group at nitrogen [34]. However, in solid state, complexes **1**–**3** exhibit exclusively the *N*,*N* arrangement (vide infra), which is in good agreement with previous observations [16,36]. Complexes **2** and **3** feature the desired dangling arms with intramolecular amide hydrogen bond donors, confirmed by the broad singlet resonances in the ^1^H-NMR spectra. In addition, ^1^H-NMR spectroscopy reveals a pronounced downfield shift of the resonances for the amide proton in comparison to the free ligand (e.g., 6.12 vs. 4.86 ppm, **2** vs. **HL2**, C_6_D_6_), presumably indicating hydrogen bonding to an oxido ligand in solution [50,51,52,53]. The bidentate coordination mode of **L2** and **L3** is in agreement with previous observations for iminophenolate complexes with or without additional donor atoms, whereas related aminophenolate ligands coordinate in a facial tridentate fashion due to their higher coordinative flexibility [15,16,18].

As expected, complexes **1**–**3** are sensitive to moisture. Whereas complex **1** is well soluble in most common laboratory solvents including aliphatic hydrocarbons, complex **2** is well soluble in chlorinated hydrocarbons, benzene and toluene, but shows poor solubility in MeCN and MeOH. Complex **3** is well soluble in CH_2_Cl_2_, but exhibits only limited solubility in other standard laboratory solvents. The composition as well as the (mononuclear) structure of complexes **1**–**3** was confirmed by spectroscopic measurements (^1^H- and ^13^C-NMR, IR), mass spectrometry, elemental analysis and single-crystal X-ray diffraction analysis (vide infra).

### 2.3. Reduction of Complexes ***1***–***3*** and Activation of Dioxygen

**Figure 2:**
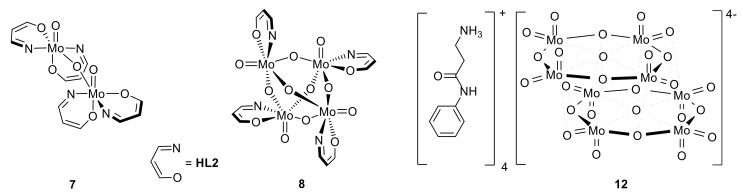
Polynuclear compounds **7** and **8** forming during reduction of **2** with PMe_3_, and **12** forming during reduction of **3** with PMe_3_ under O_2_ atmosphere.

**Scheme 3:**
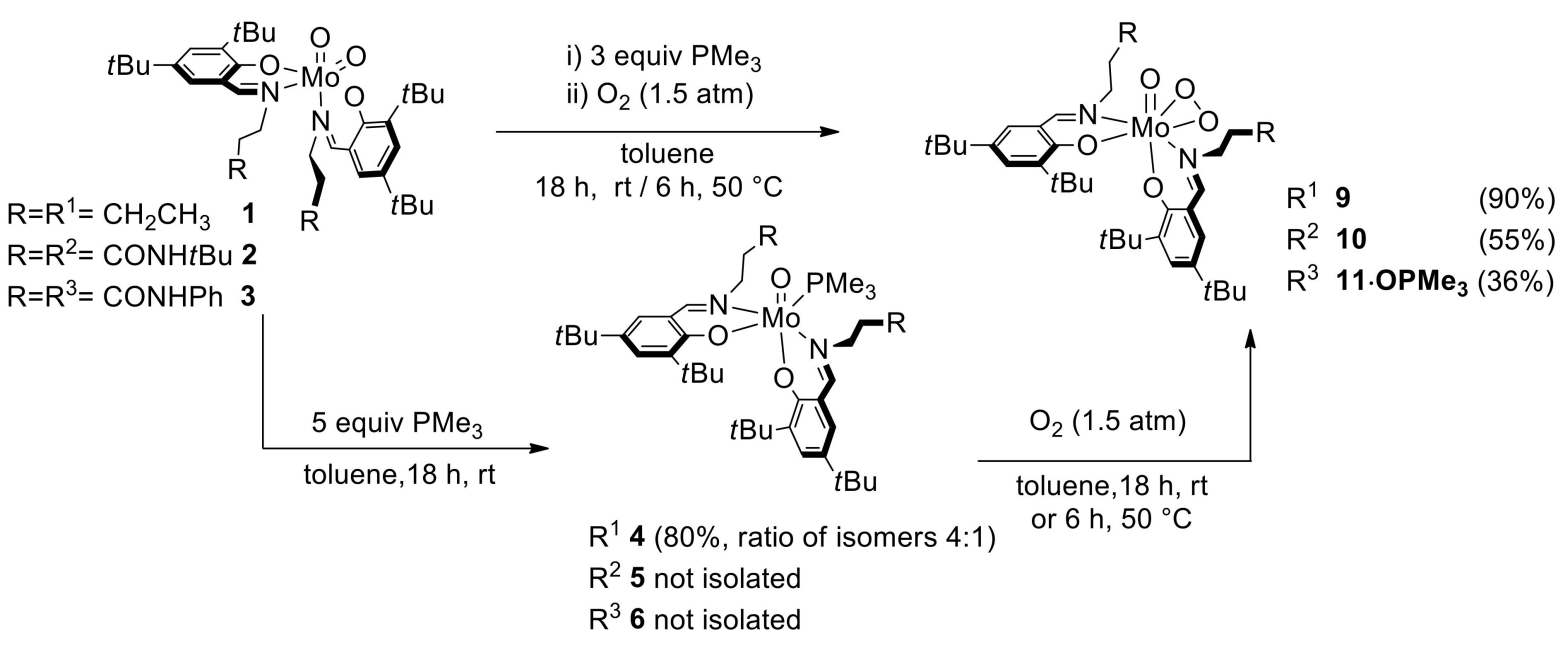
Oxygen atom transfer (OAT) reactivity of complexes **1**–**3** and activation of O_2_ to form oxido peroxido compounds **9**–**11**. For clarity, only the major isomer is depicted for all complexes.

Reaction of [MoO_2_(**L1**)_2_] (**1**) with five equiv of PMe_3_ resulted in the formation of the phosphane coordinated Mo(IV) oxido compound [MoO(PMe_3_)(**L1**)_2_] (**4**) in good yield (Scheme 3). Similar to the ^1^H-NMR resonances for **1**, also compound **4** features signals for two species, reflected by two distinct sets of resonances for two ligands each. Additionally, two distinct resonances for coordinated PMe_3_ are observed in the ^31^P{^1^H}-NMR spectrum_._ The isomeric ratio for compound **4** is approximately 4:1, with the major isomer probably adopting the *O*,*O* conformation, which is in accordance with previous reports [36]. Addition of five equiv of PMe_3_ to complexes **2** or **3** led to a similar mixture of isomers of compounds [MoO(PMe_3_)(**L2**)_2_] (**5**) and [MoO(PMe_3_)(**L3**)_2_] (**6**). ^1^H- and ^31^P{^1^H}-NMR spectroscopy revealed for **5** also two and for **6** at least three isomers in solution. Although these data point to the actual formation of **5** and **6**, we were unable to isolate them in pure form. Any purification attempts led to the formation of several new, presumably polynuclear species together with free ligand. Nevertheless, during such attempts with **5**, two types of single crystals were obtained revealing the formation of a µ-oxido bridged molybdenum(V) dimer, {[MoO(**L2**)_2_]_2_(µ-O)} (**7**), which is similar to previous observations [36], as well as a tetranuclear µ-oxido bridged molybdenum(VI) cluster, {[MoO(**L2**)]_4_(µ-O)_6_} (**8**), as depicted in Figure 2. We attribute the increased instability and the resulting decomposition to the additional acidic functionalities in the ligand side chains. The tetranuclear structure of **8** has not been described before in molybdenum coordination chemistry; only a tungsten derivative is known [54]. In contrast, the structural motifs of **7** and **12** (vide infra) are quite common [36,55,56,57].

Complex **4** is very well soluble in most common organic solvents including aliphatic hydrocarbons. The compound is highly sensitive to moisture and air and tends to slowly decompose in solution. The composition of **4** was additionally confirmed via FT-IR spectroscopy and elemental analysis. Compound **5** was characterized in situ via ^1^H-, ^13^C- and ^31^P{^1^H}-NMR spectroscopy under inert conditions. Compounds **7** and **8** were only obtained in small quantities of single crystals and thus were only characterized via single-crystal X-ray diffraction analysis.

The phosphane coordinated complex **4** cleanly reacts with dioxygen, which is indicated by a quick color change from red-brown to orange-red, to form the desired oxido peroxido compound [MoO(O_2_)(**L1**)_2_] (**9**) in excellent yield. Alternatively, **9** is also accessible in a one-pot reaction via the addition of three equiv of PMe_3_ to a toluene solution of **1** and subsequent stirring under O_2_ atmosphere for 18 h, which is similar to previous reports [34,36]. Such a direct formation of the oxido peroxido species from the corresponding molybdenum dioxido complex was also investigated for complexes **2** and **3** (Scheme 3). For the synthesis of [MoO(O_2_)(**L2**)_2_] (**10**), the same protocol as described above could be applied. A noteworthy difference, however, was the work-up procedure, since removal of residual OPMe_3_ via an extraction protocol using benzene or alkanes resulted in the isolation of a 1:1 mixture of **10** with OPMe_3_. This mixture was subsequently identified via single-crystal X-ray diffraction as **10**·OPMe_3_ in which OPMe_3_ is hydrogen-bonded to the amide (vide infra). Nonetheless, recrystallization of the crude reaction product from the coordinating solvent MeCN yielded microcrystalline **10** in good yield as well as single crystals of this OPMe_3_-free compound. For the formation of [MoO(O_2_)(**L3**)_2_] (**11**), an analogous reaction at room temperature was examined. However, only a slow reaction progress as well as side product formation (presumably a dimeric compound) was observed. An increase of the reaction temperature to 50 °C led to the formation of the desired compound and additionally allowed for a shorter reaction (6 h). Similar to **10**, after work-up, a 1:1 mixture of **11** and OPMe_3_ was observed. In contrast to **10**, however, recrystallization from MeCN did not remove the OPMe_3_, presumably because of the more acidic amide N-H groups and thus stronger hydrogen bonds. Compound **11** was thus isolated as **11**·OPMe_3_ in fair yield. Slow evaporation of a concentrated MeCN solution of **11**·OPMe_3_ led to single crystals of two distinct adducts of **11** (**11**·OPMe_3_ and **11**·2OPMe_3_) as well as a small quantity of crystals, which could be identified as a hydrolysis/decomposition product of the formula [C_9_H_13_N_2_O]_4_[Mo_8_O_26_]·6OPMe_3_ (**12**, Figure 2). The sensitivity of **11** towards hydrolysis is noteworthy, since it is in contrast to previously investigated molybdenum(VI) oxido peroxido complexes [35,36]. Complex **9** exists as a mixture of two isomers in solution, with an isomeric ratio of >10:1, as determined by ^1^H-NMR spectroscopy; for complexes **10** and **11**, only a single isomer is observed. All three complexes were found to adopt the *O*,*O* isomer in solid state via single-crystal X-ray diffraction analysis and, based on spectroscopic evidence, the isomeric conformation is the same in solution, which is in agreement with previous observations [14,33,34,36]. In compound **10**, the ^1^H-NMR resonances for the amide protons exhibit a slight upfield shift in comparison to [MoO_2_(**L2**)_2_] (**2**), which is in contrast to the solid state structures (vide infra), where no hydrogen bond is observed in **2** but a weak interaction to the peroxido group in **10**. This finding suggests generally flexible and weak interactions in our complexes. The amide resonances in **11** are shifted downfield in comparison to **3**, which is probably caused by hydrogen bonding to OPMe_3_. Complexes **9**–**11** are well soluble in most common laboratory solvents and moderately soluble in alkanes. Whereas complexes **9** and **10** are stable to air and only slightly sensitive to moisture, complex **11** tends to decompose within days upon exposure to moisture.

### 2.4. Molecular Structures

**Figure 3:**
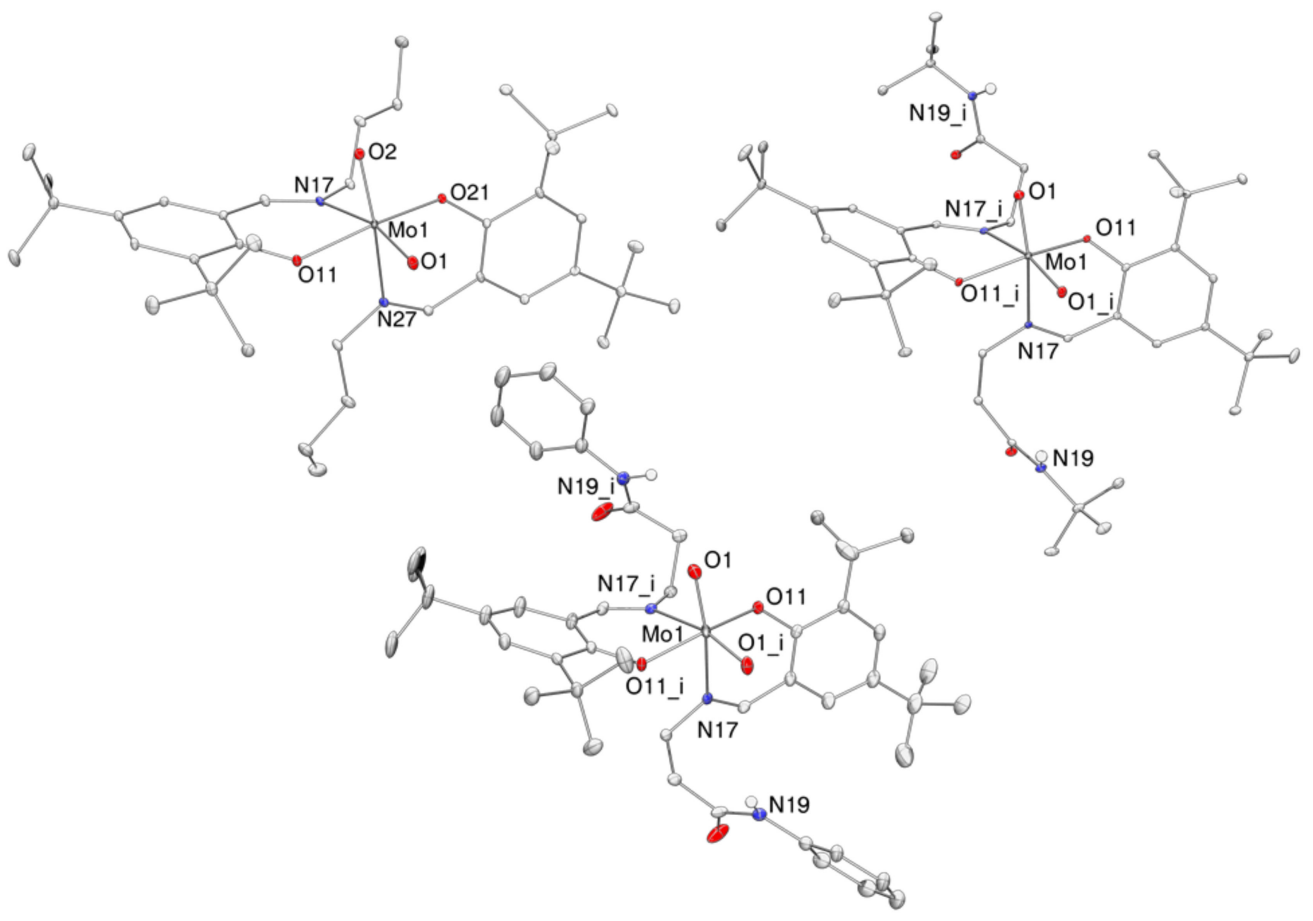
Molecular views (50% probability level) of the dioxidomolybdenum(VI) complexes [MoO_2_(**L1**)_2_]^15^ (**1**, top left), [MoO_2_(**L2**)_2_] (**2**, top right) and [MoO_2_(**L3**)_2_] (**3**, bottom). H atoms, except amide protons, as well as solvent molecules are omitted for clarity reasons.

**Figure 4:**
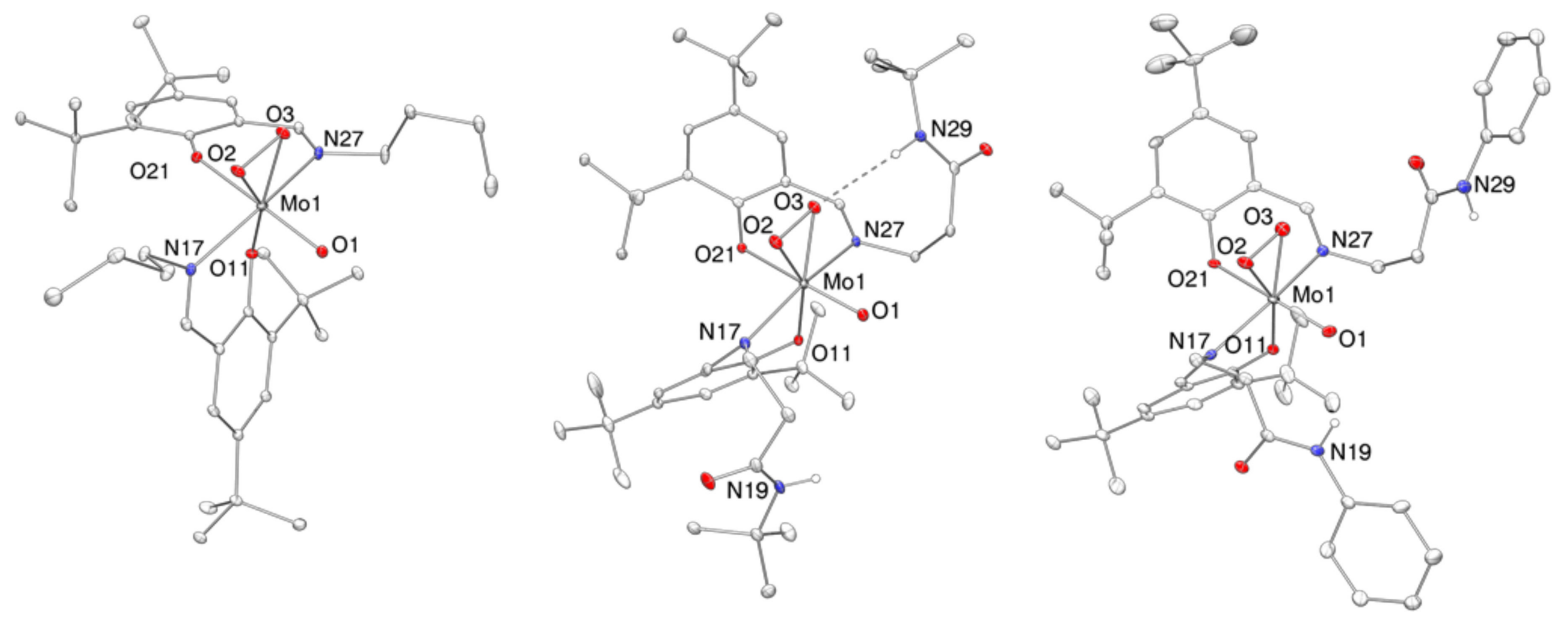
Molecular views (50% probability level) of the oxido peroxido molybdenum(VI) complexes [MoO(O_2_)(**L1**)_2_] (**9**, left), [MoO(O_2_)(**L2**)_2_] (**10**, middle) and [MoO(O_2_)(**L3**)_2_] (**11**, right). H atoms, except amide protons, as well as solvent molecules and OPMe_3_ (in **11**) are omitted for clarity reasons.

**Figure 5:**
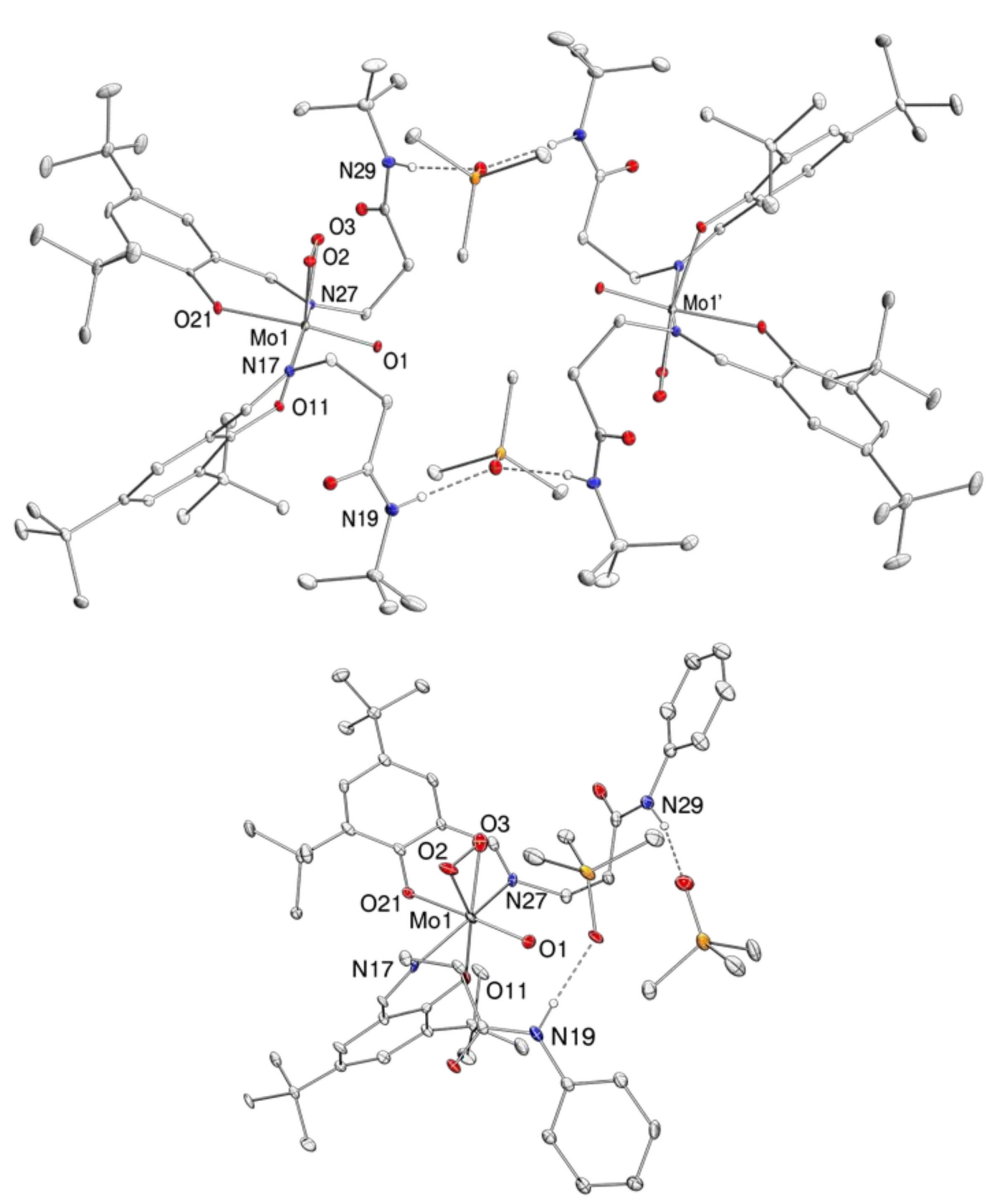
Molecular views (50% probability level) of the hydrogen bonds in the oxido peroxido complex adducts {[MoO(O_2_)(**L2**)_2_]_2_(OPMe_3_)_2_} (**10**·OPMe_3_, top) and {[MoO(O_2_)(**L3**)_2_](OPMe_3_)_2_} (**11**·2OPMe_3_, bottom). H atoms, except amide protons, as well as solvent molecules are omitted for clarity reasons.

Molecular structures of the ligand **HL3**, molybdenum(VI) dioxido complexes **1**–**3**, as well as molybdenum(VI) oxido peroxido complexes **9**, **10**, **10**·OPMe_3_, **11**·OPMe_3_, **11**·2OPMe_3_ and the side products **7**, **8** and **12** were determined by single-crystal X-ray diffraction analysis. Molecular views of **1**–**3** are given in Figure 3, molecular views of **9**, **10** and **11**(OPMe_3_) in Figure 4 and the intermolecular hydrogen bonding in the phosphane oxide adducts of **10**·OPMe_3_ and **11**·2OPMe_3_ in Figure 5. The molecular views of the ligand **HL3**, the monosubstituted complex **3^acac^** as well as the polynuclear compounds **7**, **8** and **12** are depicted in Figures S2–S6 in the Supplementary Materials. Selected bond lengths and angles for complexes **1**–**3** are provided in Table 1 and for complexes **9**, **10** and **11**·2OPMe_3_ in Table 2. Full crystallographic details such as structure refinement as well as experimental details are provided within the Supplementary Materials.

In complexes **1**–**3**, the molybdenum atoms are coordinated in a distorted octahedral fashion by two bidentate ligands and two oxido ligands, with the iminophenolate ligands adopting the *N*,*N* isomeric form with respect to the oxido *trans* positions. Hydrogen bonding from the pendant amide functionalities to the oxido ligands is not observed. The bond lengths to all ligands are very similar in complexes **1**–**3**; only the Mo–N bonds are slightly shorter in complex **3**. The Mo=O bond lengths are within expected ranges (Table 1) [58].

In compounds **9**–**11**, the molybdenum centers are coordinated by two bidentate iminophenolate ligands, an oxido ligand and a η^2^ side-on coordinated peroxido ligand in a distorted octahedral fashion. All three compounds adopt the *O*,*O* isomeric form in solid state, with the phenolate oxygen atoms *trans* to the oxido and peroxido ligand, respectively. The change of the isomeric form, with respect to the parent complexes **1**–**3**, is reflected by the N17-Mo1-N27 and O11-Mo-O21 bond angles. Additionally, the Mo–N bonds are shortened by approx. 0.15 Å on average, whereas the Mo–O (phenolate) bonds are slightly elongated, caused by the *trans* influence of the oxido (and peroxido) ligands. The bonds to the bidentate ligands as well as the Mo=O bonds are of similar length in all oxido peroxido complexes in the observed modifications. Comparison of the Mo–(O–O) bond lengths of all three complexes shows a rather small influence of the ligand. The bond lengths in **10** follow the expected trend in comparison to **9**, namely a shortening of the Mo–(O–O) bonds as well as a slight elongation of the (O–O) bond due to the electron richer *tert*-butyl system. The shorter (O–O) bond in complex **11** could then be explained by the rather electron withdrawing character of the phenyl system.

The amido hydrogen atoms of compounds **10** and **11** are in principle involved in hydrogen bonding. Since the latter was only isolable with additional OPMe_3_ molecules, hydrogen bonding to the oxygen atom of the phosphine occurred exclusively (Figure 5, bottom). In compound **10**, a weak intramolecular hydrogen bond from the amide H to one O atom of the peroxido moiety is formed (Table S18: N29-H29-O3, D-H···A: d(D-H) 0.88 Å, d(H···A) 2.277(7) Å, d(A-D) 3.0718(19) Å, <(DHA) 150.1(12)°). However, comparison of the O–O bond length in **10** to that of **9** (without hydrogen bond) reveals little influence (Table 2). While the pending substituent is able to rotate into the right position or distance to the peroxido ligand, the flexibility seems to be too high for effective hydrogen bonding in the desired fashion. However, hydrogen bonding might facilitate reactivity by bringing the substrate in close proximity to the peroxido group.

### 2.5. Catalysis

The catalytic aerobic oxidation of trimethyl phosphane as a widespread model reaction using complexes **1**–**3**, according to Equation (1), was investigated. Used conditions were 1 mol% catalyst under 1 atm of dry O_2_ gas in C_6_D_6_ in 100 mL Schlenk flasks. Conversions were determined after 24 h by ^31^P{^1^H}-NMR spectroscopy. Although PMe_3_ is easily oxidized without any metal in presence of water and air, the stability of PMe_3_ against dry O_2_ is high as blank experiments under identical conditions but without catalysts led to a conversion of PMe_3_ <5%.


(1)
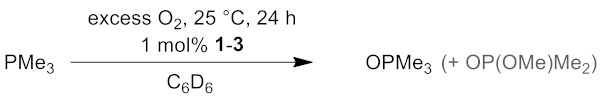



The highest conversion of trimethyl phosphane was observed for catalyst **2**, bearing *tert*-butyl amide functionalities, with a phosphane oxide yield of 53% accompanied by 4% of the previously observed side product methyl dimethylphosphinate (Table 3) [36]. Catalyst **1**, without hydrogen bond donors, selectively oxidized 46% of PMe_3_ to OPMe_3_ within 24 h. Both values are comparable to other molybdenum dioxido systems [36]. This is in line with the formation of a weak hydrogen interaction, which is not sufficient to increase the reactivity. Interestingly, complex **3**, equipped with a phenyl amide group, led only to 25% conversion (23% OPMe_3_). This finding is corroborated by the solid-state structures where the OPMe_3_ molecule is retained by hydrogen bonding thereby decreasing catalytic reactivity. Another possibility is the formation of polynuclear compounds during the reaction, as observed in the synthesis of the peroxido complex **11** (*vide supra*). With more challenging substrates such as cyclooctene or also SMe_2_ no conversion was observed.

## 3. Experimental Section

### 3.1. General

Unless specified otherwise, experiments were performed under inert conditions using standard Schlenk equipment. Chemicals were purchased from commercial sources and used as received. No further purification or drying operations have been performed. The metal precursors [MoO_2_(acac)_2_] [48] and [MoO_2_Br_2_(DMSO)_2_] [59] as well as the compound 3-(((benzyloxy)carbonyl)amino)propanoic acid [47] were synthesized according to published procedures. Solvents were purified via a Pure–Solv MD–4–EN solvent purification system from Innovative Technology, Inc. (Oldham, UK). Methanol was refluxed over activated magnesium for at least 24 h and then distilled prior to use. The ^1^H-, ^13^C-, ^31^P- and HSQC-NMR spectra were recorded on a Bruker Avance III instrument at 300/75/121 MHz (Bruker, Billerica, MA, USA). Peaks are denoted as singlet (s), broad singlet (bs), doublet (d), doublet of doublets (dd), doublet of triplets (dt), triplet (t), triplet of doublets (td) and multiplet (m). Used solvents and peak assignment are mentioned at the specific data sets. Electron impact mass spectroscopy (EI-MS) measurements have been performed with an Agilent 5973 MSD mass spectrometer with push rod (Agilent Technologies Inc., Santa Clara, CA, USA). Peaks are denoted as cationic mass peaks, and the unit is the according ion’s mass/charge ratio. Gas chromatography mass spectroscopy (GC–MS) measurements have been performed with an Agilent 7890 A gas chromatograph (column type, Agilent 19091J-433, Agilent Technologies Inc., Santa Clara, CA, USA), coupled to an Agilent 5975 C mass spectrometer. Samples for infrared spectroscopy were measured on a Bruker Optics ALPHA FT-IR spectrometer (Bruker, Billerica, MA, USA). IR bands are reported with wavenumber (cm^−1^) and intensities (s, strong; m, medium; w, weak). All elemental analyses were measured at the University of Technology of Graz, Institute of Inorganic Chemistry using an Elementar vario MICRO cube analyzer (Elementar, Langenselbold, Germany).

### 3.2. X–ray Diffraction Analyses

Single-crystal X-ray diffraction analyses were measured on a BRUKER–AXS SMART APEX II diffractometer equipped with a CCD detector (Bruker-AXS, Karlsruhe, Germany). All measurements were performed using monochromatized Mo K_α_ radiation from an Incoatec microfocus sealed tube at 100 K (Tables S1 and S2). Absorption corrections were performed semi–empirical from equivalents. Structures were solved by direct methods (SHELXS-97) [60] and refined by full–matrix least–squares techniques against *F*^2^ (SHELXL-2014/6) [61]. CCDC numbers 1865601–1865613 contain the supplementary crystallographic data for this paper. These data can be obtained free of charge from The Cambridge Crystallographic Data Centre via www.ccdc.cam.ac.uk/data_request/cif. Full experimental details for single-crystal X-ray diffraction analyses of all compounds are provided in the Supplementary Materials. 

### 3.3. Ligand Syntheses

All ligands are stable towards air but slightly sensitive towards moisture. They can be stored in a desiccator over P_2_O_5_ for several weeks without decomposition. 

#### 3.3.1. Synthesis of 2,4-Di-*tert*-butyl-6-((butylimino)methyl)phenol (**HL1**)

Ligand **HL1** was synthesized according to literature procedure [46]. Analytical data are consistent with literature, ^1^H- and ^13^C-NMR shifts in C_6_D_6_ as well as IR absorption bands are given for comparison reasons. ^1^H-NMR (300 MHz, C_6_D_6_, 25 °C) δ: 14.33 (s, 1H, OH), 7.82 (s, 1H, CH=N), 7.58 (d, 1H, ArH), 7.00 (d, 1H, ArH), 3.15 (td, 2H, CH_2_), 1.67 (s, 9H, *t*Bu), 1.43–1.33 (m, 2H, CH_2_), 1.35 (s, 9H, *t*Bu), 1.26–1.14 (m, 2H, CH_2_), 0.79 (t, 3H, CH_3_) ppm; ^13^C-NMR (75 MHz, C_6_D_6_, 25 °C) δ: 165.89 (C=N), 159.00 (Ar-O), 140.03, 137.17, 126.78, 126.25, 118.72 (Ar), 59.40 (CH_2_), 35.48, 34.33 (q-*t*Bu), 33.25 (CH_2_), 31.81, 29.83 (*t*Bu), 20.63 (CH_2_), 13.98 (CH_3_) ppm. IR (ATR, cm^−1^) δ: 3316 (w, OH + NH), 2955 (w, C-H), 1668 (m, C=O), 1629 (m, C=N), 1600 (s), 1543 (s), 1439 (s), 1310 (m), 1249 (m), 828 (m), 755 (s), 693 (s), 503 (m); IR (ATR, cm^−1^) ῦ: 3437 (br w, OH), 2955 (s, C-H), 1632 (s, C=N), 1466 (s), 1440 (s), 1361 (m), 1273 (s) 1251 (s), 1173 (s), 827 (m), 772 (m).

#### 3.3.2. Synthesis of *N*-(*tert*-Butyl)-3-((3,5-di-*tert*-butyl-2-hydroxybenzylidene)amino)-propanamide (**HL2**)

For the synthesis of **HL2**, a three-step procedure was applied. In the first step, 1 equiv of 3-(((benzyloxy)carbonyl)amino)propanoic acid (10.00 g, 44.8 mmol) and 1 equiv Et_3_N (6.2 mL, 44.8 mmol) were dissolved in 100 mL of dry toluene and subsequently cooled to −12 °C (ice/NaCl bath). Subsequently, 1 equiv of ethyl chloroformate (4.4 mL, 44.8 mmol) was added dropwise over 30 min while keeping the temperature below −10 °C. After stirring of the reaction solution at −12 °C for 30 min, 1 equiv of *tert*-butyl amine (4.7 mL, 44.8 mmol) in 10 mL dry dichloromethane was added dropwise over 15 min while keeping the temperature below −10 °C. Subsequently, the reaction mixture was stirred for 30 min at −12 °C, warmed to room temperature and finally stirred under reflux for 10 min. The reaction was then stirred at room temperature overnight. The subsequent work-up was performed at ambient conditions. After addition of 200 mL chloroform, the reaction mixture was washed two times with saturated aqueous NaHCO_3_ solution and deionized water (100 mL each). The organic phase was dried over MgSO_4_ and evaporated in vacuo. The crude product was recrystallized from ethyl acetate to yield benzyl (3-oxo-3-(phenylamino)propyl)carbamate as a white crystalline solid (68%, 8.53 g). 

In the second step, 1 equiv of benzyl (3-oxo-3-(phenylamino)propyl)carbamate (4.67 g, 17.0 mmol) was dissolved in 100 mL of MeOH. Subsequently 15 w% Pd/C (0.70 g) were added and the reaction mixture stirred under an H_2_ atmosphere (5 atm) overnight at room temperature. Filtration and evaporation of the filtrate in vacuo gave 3-amino-*N*-(*tert*-butyl)propanamide as light yellow oil (94%, 2.28 g). In the final step, 1.1 equiv of 3-amino-*N*-(*tert*-butyl)propanamide (1.00 g, 6.9 mmol) were added to a solution of 1 equiv of 3,5-di-*tert*-butyl-2-hydroxybenzaldehyde (1.48 g, 6.3 mmol) in 5 mL of MeOH. The resulting deep yellow solution was subsequently stirred at 50 °C for 3 h. Evaporation of all volatiles in vacuo led to a deep yellow oil that solidified over the course of several days. Washing of the crude product twice with 5 mL of pentane gave **HL2** as bright yellow solid (88%, 1.99 g, overall yield 56%). ^1^H-NMR (300 MHz, C_6_D_6_, 25 °C) δ: 14.06 (s, 1H, OH), 7.90 (s, 1H, CH=N), 7.56 (d, 1H, ArH). 6.96 (d, 1H, ArH), 4.86 (bs, 1H, NH), 3.54 (t, 2H, CH_2_), 1.93 (t, 2H, CH_2_), 1.66 (s, 9H, *t*Bu), 1.30 (s, 9H, *t*Bu), 1.21 (s, 9H, *t*Bu) ppm; ^13^C-NMR (75 MHz, C_6_D_6_, 25 °C) δ: 169.57 (C=O), 167.33 (C=N), 158.72 (Ar-O), 140.23, 136.92, 126.93, 126.58, 118.62 (Ar), 55.81 (CH_2_), 50.99 (q-*t*Bu), 38.68 (CH_2_), 35.42, 34.29 (q-*t*Bu), 31.74, 29.81, 28.74 (*t*Bu) ppm; EI-MS: *m*/*z* 360.4 ([M]^+^); IR (ATR, cm^−1^) ῦ: 3322 (w, OH + NH), 2952 (m, C-H), 1639 (s, C=O), 1629 (s, C=N), 1541 (s), 1438 (s), 1390 (m), 1276 (m).

#### 3.3.3. Synthesis of 3-((3,5-Di-*tert*-butyl-2-hydroxybenzylidene)amino)-*N*-phenylpropan-amide (**HL3**)

For the synthesis of **HL3**, a two-step procedure was applied. In the first step, 3-amino-*N*-phenylpropanamide was synthesized following a published procedure (yield: 58%) [62]. In the second step, 1 equiv of 3-amino-*N*-phenylpropanamide (1.45 g, 8.8 mmol) was added to a solution of 1 equiv of 3,5-di-*tert*-butyl-2-hydroxybenzaldehyde (2.07 g, 8.8 mmol) in 20 mL of MeOH. The resulting yellow solution was stirred at reflux temperature overnight and subsequently evaporated in vacuo. The crude product was washed thoroughly with pentane to give **HL3** as pale yellow solid (84%, 2.83 g, 48% overall yield). Crystals suitable for single-crystal X-ray diffraction analysis were obtained via crystallization from a concentrated C_6_D_6_ solution of **HL3** at 25 °C. ^1^H-NMR (300 MHz, C_6_D_6_, 25 °C) δ: 14.02 (s, 1H, OH), 7.91 (s, 1H, CH=N), 7.56 (d, 1H, ArH). 7.56–7.50 (m, 2H, ArH), 7.12–7.04 (m, 2H, ArH), 6.95 (d, 1H, ArH), 6.89–6.82 (m, 1H, ArH), 6.69 (bs, 1H, NH), 3.54 (t, 2H, CH_2_), 2.01 (t, 2H, CH_2_), 1.65 (s, 9H, *t*Bu), 1.30 (s, 9H, *t*Bu), 1.21 (s, 9H, *t*Bu) ppm; ^13^C-NMR (75 MHz, C_6_D_6_, 25 °C) δ: 168.69 (C=O), 167.80 (C=N), 158.75 (Ar-O), 140.34, 138.64, 137.00, 129.11, 127.14, 126.68, 124.25, 120.10, 118.57 (Ar), 55.18 38.23 (CH_2_), 35.43, 34.29 (q-*t*Bu), 31.71, 29.79 (*t*Bu) ppm; EI-MS: *m*/*z* 380.4 ([M]^+^); IR (ATR, cm^−1^) ῦ: 3316 (w, OH + NH), 2955 (w, C-H), 1668 (m, C=O), 1629 (m, C=N), 1600 (s), 1543 (s), 1439 (s), 1310 (m), 1249 (m), 828 (m), 755 (s), 693 (s), 503 (m).

### 3.4. Complex Syntheses

All complexes except **4**–**6** are stable towards air but sensitive towards moisture and can be stored in a desiccator over P_2_O_5_ for several weeks without decomposition. Complex **4** is sensitive towards air and moisture and can be stored in an N_2_-filled glovebox for several weeks without decomposition; compounds **5** and **6** could not be isolated due to substantial decomposition during work-up.

#### 3.4.1. Alternative Synthesis of [MoO_2_(**L1**)_2_] (**1**)

For the synthesis of **1**, 1 equiv of [MoO_2_Br_2_(DMSO)_2_] (610 mg, 1.37 mmol) was suspended in dry toluene (10 mL). Subsequently, 2 equiv of **HL1** (800 mg, 2.74 mmol) as well as 2.5 equiv of NEt_3_ (48 µL, 3.43 mmol) were added and the deep orange reaction mixture stirred at 60 °C for 18 h. The orange reaction mixture was subsequently concentrated in vacuo and stored at –25 °C for 24 h. The precipitate was filtered off and washed with little dry toluene and subsequently twice with dry pentane to give **1** as a yellow microcrystalline powder (57%, 550 mg). Crystals suitable for single-crystal X-ray diffraction analysis were obtained via crystallization from the residual filtrate at 5 °C (**1**·NEt_3_·HBr). Analytical data is consistent with the literature [16], ^1^H- and ^13^C-NMR shifts in C_6_D_6_ as well as IR data is given for comparison reasons. ^1^H-NMR (300 MHz, C_6_D_6_, 25 °C, major isomer) δ: 7.85 (s, 2H, CH=N), 7.72 (d, 2H, ArH), 7.12 (d, 2H, ArH), 3.50–3.29 (m, 4H, CH_2_), 1.66 (s, 18H, *t*Bu), 1.66–1.16 (m, 4H, CH_2_), 1.30 (s, 18H, *t*Bu), 1.05–0.98 (m, 4H, CH_2_), 0.69 (t, 6H, CH_3_) ppm; ^13^C-NMR (75 MHz, C_6_D_6_, 25 °C, major isomer, 1 Ar concealed by solvent) δ: 167.15 (CH=N), 161.15 (Ar–O), 142.04, 139.60, 129.64, 122.16 (Ar), 60.32 (CH_2_), 35.60, 34.40 (q-*t*Bu), 33.68 (CH_2_), 31.61, 30.35 (*t*Bu), 20.68 (CH_2_), 13.84 (CH_3_) ppm; IR (ATR, cm^−1^): ῦ 2957 (m, C-H), 1628 (s, C=N), 1439 (m), 1270 (s), 1249 (s), 917 (m, Mo=O), 904 (s, Mo=O), 844 (s), 754 (m), 552 (s, Mo-O), 431 (m).

#### 3.4.2. Synthesis of [MoO_2_(**L2**)_2_] (**2**)

For the synthesis of **2**, 2 equiv of **HL2** (150 mg, 0.42 mmol) as well as 1 equiv of [MoO_2_(acac)_2_] (68 mg, 0.21 mmol) were dissolved in dry MeOH (5 mL). The deep orange reaction mixture was heated to 50 °C and stirred for 18 h, whereupon a bright yellow precipitate formed. The mixture was cooled to 5 °C for 2 h and the precipitate subsequently filtered off and washed with a small portion of cold dry MeOH as well as cold dry pentane. Drying in vacuo gave **2** as a bright yellow microcrystalline solid (65%, 116 mg). Crystals suitable for single-crystal X-ray diffraction analysis were obtained via recrystallization from a concentrated MeCN solution at −35 °C (**2**·2MeCN). ^1^H-NMR (300 MHz, C_6_D_6_, 25 °C, major isomer (symm.)) δ: 8.02 (s, 2H, CH=N), 7.67 (d, 2H, ArH), 7.12 (d, 2H, ArH), 6.12 (bs, 2H, NH), 3.70–3.60 (m, 2H, CH_2_), 3.57–3.42 (m, 2H, CH_2_), 3.12–2.99 (m, 2H, CH_2_), 2.23–2.12 (m, 2H, CH_2_), 1.56 (s, 18H, *t*Bu), 1.26 (s, 18H, *t*Bu), 1.04 (s, 18H, *t*Bu) ppm; ^1^H-NMR (300 MHz, C_6_D_6_, 25 °C, minor isomer (asymm.)) δ: 8.22 (s, 1H, CH=N), 7.80 (s, 1H, CH=N), 7.63–7.61 (m, 2H, ArH), 7.01 (“d”, 2H, ArH), 6.05 (bs, 1H, NH), 5.80 (bs, 1H, NH), 4.47–4.38 (m, 1H, CH_2_), 3.98–3.85 (m, 1H, CH_2_), 3.57–3.42 (m, 1H, CH_2_), 3.38–3.26 (m, 1H, CH_2_), 2.56–2.40 (m, 2H, CH_2_), 2.18–2.07 (m, 1H, CH_2_), 1.64 (s, 9H, *t*Bu), 1.30 (s, 9H, *t*Bu), 1.22 (s, 9H, *t*Bu), 1.21 (s, 9H, *t*Bu), 1.15 (s, 9H, *t*Bu), 1.04 (s, 9H, *t*Bu), ppm; ^13^C-NMR (75 MHz, C_6_D_6_, 25 °C, major isomer (symm.)) δ: 169.42 (C=O), 168.29 (CH=N), 159.13 (Ar-O), 142.87, 139.64, 130.17, 129.60, 121.69 (Ar), 58.89 (CH_2_), 50.80 (q-*t*Bu), 40.31 (CH_2_), 35.60, 34.40 (q-*t*Bu) 31.50, 30.65, 28.52 (*t*Bu) ppm; IR (ATR, cm^−1^) ῦ: 3375 (w, NH), 2961 (m, C-H), 1676 (m, C=O), 1628 (C=N, s), 1524 (m), 1442 (m), 1361 (m), 1248 (s), 912 (sh, s, Mo=O), 898 (s, Mo=O), 842 (s), 752 (m), 549 (s, Mo-O), 483 (m), 436 (m); EI-MS (70 eV) *m*/*z*: 848.7 [M]^+^, 832.7 [M − O]^+^; Anal. calcd. for C_44_H_70_MoN_4_O_6_∙0.5CH_3_OH: C, 61.93; H, 8.41; N, 6.49. Found: C, 61.61, H, 8.01; N, 6.73%. 

#### 3.4.3. Synthesis of [MoO_2_(**L3**)_2_] (**3**) 

For the synthesis of **3**, 2 equiv of **HL3** (765 mg, 2.01 mmol) were suspended in a small portion of dry MeOH and mixed with 2.5 equiv of NEt_3_ (0.35 mL, 2.52 mmol). Subsequently, a solution of 1 equiv [MoO_2_Cl_2_] (200 mg, 1.01 mmol) in the same solvent (5 mL) was added dropwise to the mixture. The orange-red reaction mixture was stirred for 18 h whereupon yellow solids precipitated. The mixture was stored at −25 °C overnight and the precipitate subsequently filtered off and washed with small portions of dry MeOH and *n*-pentane. Removal of all volatiles in vacuo yielded **3** as a bright yellow solid (66%, 638 mg). Crystals suitable for single-crystal X-ray diffraction analysis were obtained via recrystallization from a concentrated MeOH solution at −35 °C (**3**·4MeOH). ^1^H-NMR (300 MHz, C_6_D_6_, 25 °C, major isomer (symm.)) δ: 8.09 (s, 2H, NH), 7.91 (s, 2H, CH=N), 7.61 (d, 2H, ArH), 7.43–7.36 (m, 4H, ArH), 6.94–6.67 (m, 8H, ArH), 3.73–3.61 (m, 2H, CH_2_), 3.48–3.37 (m, 2H, CH_2_), 3.13–2.97 (m, 2H, CH_2_), 2.35–2.24 (m, 2H, CH_2_), 1.57 (s, 18H, *t*Bu), 1.12 (s, 18H, *t*Bu) ppm. ^13^C-NMR (HSQC 300/75 MHz, C_6_D_6_, 25 °C, major isomer (symm.), q-C obscured) δ: 168.72 (CH=N), 130.89, 128.64, 128.51, 123.69, 119.38 (Ar), 59.26, 40.29 (CH_2_), 31.02, 30.42 (*t*Bu); IR (ATR, cm^−1^): ῦ: 3320 (m, NH), 2952 (m, C-H), 1666 (s, C=O), 1619 (s, C=N), 1601 (s), 1542 (s), 1442 (s), 1270 (m), 1176 (m), 929 (w), 914 (sh, s, Mo=O), 900 (s, Mo=O), 846 (s), 752 (s), 692 (m), 549 (m, Mo-O), 496 (m), 432 (w); Anal. calcd. for C_48_H_62_MoN_4_O_6_: C, 65.00; H, 7.05; N, 6.32. Found: C, 64.98, H, 7.39; N, 6.32%.

#### 3.4.4. Synthesis of [MoO(PMe_3_)(**L1**)_2_] (**4**)

For the synthesis of **4**, 1 equiv of 1 (125 mg, 0.18 mmol) was dissolved in dry toluene (5 mL) in a Schlenk tube under N_2_ atmosphere. Subsequently 5 equiv of PMe_3_ (95 μL, 0.90 mmol) were added, whereupon the reaction mixture changed color to reddish brown. The reaction mixture was stirred for 18 h at room temperature. Subsequently all volatiles were evaporated in vacuo, cold dry heptane (3 mL) was added to the crude dark residue and insoluble OPMe_3_ was removed via filtration over a glass frit packed with Celite. Evaporation of all volatiles gave **4** as a red-brown solid (80%, 110 mg). ^1^H-NMR (300 MHz, C_6_D_6_, 25 °C, major isomer) δ: 8.04 (s, 1H, CH=N), 7.90 (s,1H, CH=N), 7.50 (d, 1H, ArH), 7.40 (d, 1H, ArH), 7.18 (d, 1H, ArH), 7.03 (d, 1H, ArH), 4.22–3.86 (m, 3H, CH_2_), 3.77–3.63 (m, 1H, CH_2_), 2.59–2.42 (m, 1H, CH_2_), 2.22–1.87 (m, 3H, CH_2_), 1.45–1.20 (m, 4H, CH_2_), 1.36 (s, 27H, *t*Bu), 1.29 (s, 9H, *t*Bu), 1.01–0.82 (m, 6H, CH_3_), 0.92 (d, 9H, PMe_3_) ppm; ^13^C-NMR (75 MHz, C_6_D_6_, 25 °C, major isomer, 1 Ar obscured) δ: 168.10, 165.93 (CH=N), 162.38, 162.24 (Ar–O), 140.24, 138.53, 136.44, 136.26, 131.03, 129.90, 129.24, 121.25, 121.05 (Ar), 35.64, 35.48 (q-*t*Bu), 35.28 (CH_2_), 34.26, 34.01 (q-*t*Bu) 33.07 (CH_2_) 31.89, 31.79, 30.12, 29.99 (*t*Bu), 21.42, 20.53, 18.61, 17.70 (CH_2_), 16.84, 16.59 (CH_3_), 14.25 (d, PMe_3_) ppm; ^31^P{^1^H}-NMR (121 MHz, C_6_D_6_, 25 °C, major isomer): −3.04 ppm; IR (ATR, cm^−1^) ῦ: 2952 (s, C-H), 1611 (s, C=N), 1433 (s), 1308 (m), 1254 (s), 952 (m), 911 (s, Mo=O), 836 (s), 745 (m), 524 (w, Mo-O), 443 (m); Anal. calcd for C_41_H_69_MoN_2_O_3_P: C, 64.38; H, 9.09; N, 3.66 found: C, 64.32; H, 8.83; N, 3.57%.

#### 3.4.5. Characterization of [MoO(PMe_3_)(**L2**)_2_] (**5**)

For the characterization of **5**, 5 equiv of PMe_3_ (10 μL, 0.10 mmol) were added to a solution of 1 equiv of 2 (20 mg, 0.02 mmol) in dry C_6_D_6_ (0.5 mL), whereupon the solution turned reddish brown. NMR spectra (^1^H, ^13^C, ^31^P) were recorded after 18 h. ^1^H-NMR (300 MHz, C_6_D_6_, 25 °C, major isomer) δ: 8.19 (s, 1H, CH=N), 8.08 (s, 1H, CH=N), 7.45 (d, 1H, ArH), 7.38 (d, 1H, ArH), 7.18 (d, 1H, ArH), 7.00 (d, 1H, ArH), 6.86 (br s, 1H, NH), 5.71 (br s, 1H, NH), 4.57–4.50 (m, 2H, CH_2_), 4.38–4.13 (m, 2H, CH_2_), 3.46–3.31 (m, 1H, CH_2_), 3.28–3.11 (m, 1H, CH_2_), 3.07–2.92 (m, 1H, CH_2_), 2.71–2.60 (m, 1H, CH_2_), 1.33 (s, 9H, *t*Bu), 1.32 (s, 9H, *t*Bu), 1.31 (s, 9H, *t*Bu), 1.29 (s, 9H, *t*Bu), 1.28 (s, 9H, *t*Bu), 1.21 (s, 9H, *t*Bu), 0.81 (d, 9H, coord. PMe_3_) ppm; ^13^C-NMR (75 MHz, C_6_D_6_, 25 °C, major isomer) δ: 170.24, 169.82 (C=O), 169.33 (C=N), 165.78 (Ar-O), 163.48 (C=N), 161.42 (Ar-O), 139.57, 139.54, 138.33, 137.04, 136.93, 131.69, 130.17, 129.61, 121.18, 121.10 (Ar), 69.16, 64.34 (CH_2_), 50.94, 50.91 (q-*t*Bu), 39.95, 39.14 (CH_2_), 35.51, 35.36, 34.27, 33.95 (q-*t*Bu), 31.80, 31.69, 29.95, 29.84, 28.96, 28.76 (tBu), 16.51 (d, coord. PMe_3_) ppm; ^31^P{^1^H}-NMR (121 MHz, C_6_D_6_, 25 °C, major isomer): −5.32 ppm.

#### 3.4.6. Synthesis of [MoO(O_2_)(**L1**)_2_] (**9**)

For the synthesis of **9**, 1 equiv of 4 (30 mg, 0.04 mmol) was dissolved in dry toluene (3 mL) in a Schlenk tube under O_2_ atmosphere (1.5 atm). Subsequently, the reaction mixture was stirred for 18 h at room temperature whereupon the color gradually changed to dark orange. After removal of all volatiles in vacuo, 3 mL of cold dry heptane were added to the crude dark orange residue and insoluble OPMe_3_ was removed via filtration over a glass frit packed with Celite. Evaporation in vacuo gave **9** as an orange solid (90%, 25 mg). Alternatively, **9** is accessible directly from **1**. Thus, 1 equiv of 1 (120 mg, 0.17 mmol) was dissolved in dry toluene (5 mL). After addition of 3 equiv of PMe_3_ in toluene (0.51 mmol, 0.5 mL 1 M solution), the reaction solution was stirred under an O_2_ atmosphere (1.5 atm) for 18 h at room temperature. The reaction mixture was subsequently evaporated to dryness; cold dry heptane (5 mL) was added and residual OPMe_3_ was removed via filtration through a glass frit packed with Celite. The filtrate was subsequently stored at −35 °C overnight, whereupon residual traces of **1** precipitated. After removal of the precipitate via filtration and evaporation of the filtrate, **9** was obtained as an orange solid (84%, 103 mg). Crystals suitable for single-crystal X-ray diffraction analysis were obtained via slow evaporation of a concentrated benzene solution at room temperature (9). ^1^H-NMR (300 MHz, C_6_D_6_, 25 °C, major isomer) δ: 8.05 (s, 1H, CH=N), 8.02 (s,1H, CH=N), 7.67 (d, 1H, ArH), 7.51 (d, 1H, ArH), 7.09 (d, 1H, ArH), 7.07 (d, 1H, ArH), 5.08–4.99 (m, 1H, CH_2_), 4.88–4.79 (m, 1H, CH_2_), 3.80–3.71 (m, 2H, CH_2_), 2.31–1.82 (m, 4H, CH_2_), 1.49–1.08 (m, 4H, CH_2_), 1.34 (s, 9H, *t*Bu), 1.29 (s, 18H, *t*Bu), 1.19 (s, 9H, *t*Bu), 0.92–0.63 (m, 6H, CH_3_) ppm; ^13^C-NMR (75 MHz, C_6_D_6_, 25 °C, major isomer) δ: 167.81, 167.29 (CH=N), 162.37, 160.66 (Ar–O), 139.70, 139.61, 139.26, 138.97, 132.01, 129.16, 128.89, 122.04, 121.88, 66.70, 62.29 (CH_2_), 35.07, 34.95, 34.16 (q-tBu), 34.12 (CH_2_), 34.03 (q-tBu), 31.79 (CH_2_), 31.63, 31.50, 29.75, 29.63(tBu), 20.71, 20.68 (CH_2_), 18.50, 14.05 (CH_3_) ppm; IR (ATR, cm^−1^) ῦ: 2954 (s, C-H), 1613 (s, C=N), 1438 (m), 1413 (m), 1253 (s), 927 (s, O-O), 915 (s, Mo=O), 841 (s), 750 (w), 540 (w, Mo-O); Anal. calcd for C_38_H_60_MoN_2_O_5_: C, 63.32; H, 8.39; N, 3.89 Found: C, 63.81; H, 8.19; N, 3.82%.

#### 3.4.7. Synthesis of [MoO(O_2_)(**L2**)_2_] (**10**)

For the synthesis of **10**, 3 equiv of PMe_3_ (18 µL, 0.18 mmol) were added to a solution of 1 equiv of **2** (50 mg, 0.06 mmol) in dry toluene (3 mL), containing 3 Å molecular sieves, and subsequently stirred under an O_2_ atmosphere (1.5 atm) for 18 h at room temperature. The reaction mixture was subsequently cooled to 5 °C, filtered and evaporated in vacuo. The resulting orange residue was dissolved in a minimum amount of dry acetonitrile, layered with dry heptane and stored in a freezer at −35 °C for 24 h. The orange precipitate was filtered off and dried to afford **10** as an orange solid (55%, 28 mg). Single crystals suitable for X-ray diffraction analysis were obtained from concentrated MeCN (**10**) or benzene (**10**·OPMe_3_) solutions at −35 °C and room temperature, respectively. ^1^H-NMR (300 MHz, C_6_D_6_, 25 °C) δ: 8.53 (s, 1H, CH=N), 8.38 (s, 1H, CH=N), 7.60 (d, 1H, ArH), 7.45 (d, 1H, ArH), 7.09 (d, 1H, ArH), 7.02 (d, 1H, ArH), 5.96 (bs, 1H, NH), 5.50 (dt, 1H, CH_2_), 5.40 (bs, 1H, NH), 5.10 (dt, 1H, CH_2_), 4.49 (td, 1H, CH_2_), 4.26 (td, 1H, CH_2_), 3.26–3.01 (m, 2H, CH_2_), 2.61–2.49 (m, 2H, CH_2_), 1.33 (s, 9H, *t*Bu), 1.21 (2s, 9H, *t*Bu), 1.21 (s, 9H, *t*Bu), 1.20 (s, 9H, *t*Bu), 1.11 (s, 9H, *t*Bu), 1.05 (s, 9H, *t*Bu), ppm; ^13^C-NMR (75 MHz, C_6_D_6_, 25 °C) δ: 170.11, 169.74 (C=O), 169.33, 169.06 (CH=N), 161.09, 160.51 (Ar-O), 140.52, 139.45, 139.35, 138.96, 132.55, 130.98, 129.99, 129.46, 121.61, 121.27 (Ar), 63.21, 59.97 (CH_2_), 51.19, 51.05 (q-*t*Bu), 39.80, 39.23 (CH_2_), 34.96, 34.79, 34.13 (2×) (q-*t*Bu), 31.52, 31.39, 29.72, 29.46, 28.66, 28.49 (*t*Bu) ppm; IR (ATR, cm^−1^) ῦ: 3272 (w, NH), 2958 (m, C-H), 1666 (m, C=O), 1611 (C=N, s), 1556 (m), 1254 (s), 931 (m, O-O), 907 (s, Mo=O), 840 (s), 541 (s, Mo-O), 463 (m); EI-MS (100 V) *m*/*z*: 848.7 [M − O]^+^, 832.7 [M − O_2_]^+^; Anal. Calcd. for C_44_H_70_MoN_4_O_7_: C, 61.24; H, 8.18; N, 6.49. Found: C, 60.14; H, 7.66; N, 6.36%. 

#### 3.4.8. Synthesis of [MoO(O_2_)(**L3**)_2_] (**11**)

For the synthesis of **11**, 3 equiv of PMe_3_ (49 μL, 0.48 mmol) were added to a suspension of 1 equiv of **3** (140 mg, 0.16 mmol) in dry toluene (5 mL), containing 3 Å molecular sieves, and the mixture subsequently stirred under an O_2_ atmosphere (1.5 atm) at 50 °C for 6 h, whereupon the solution gradually turned red. Subsequently, all volatiles were removed in vacuo. The resulting dark residue was dissolved in a minimum amount of dry acetonitrile and recrystallized at −35 °C to afford **11**·OPMe_3_ as a red crystalline solid (36%, 58 mg). Single crystals suitable for X-ray diffraction analysis were obtained from a saturated MeCN solution at −35 °C (**11**·OPMe_3_, **11**·2OPMe_3_). ^1^H-NMR (300 MHz, C_6_D_6_, 25 °C) δ: 9.29 (bs, 2H, NH), 8.56 (s, 1H, CH=N), 8.47 (s, 1H, CH=N), 7.92–7.89 (m, 4H, Ph), 7.59 (d, 1H, ArH), 7.43 (d, 1H, ArH), 7.11–7.07 (m, 4H, Ph), 7.03 (d, 1H, ArH), 6.99 (d, 1H, ArH), 6.90–6.79 (m, 2H, Ph), 5.59–5.43 (m, 1H, CH_2_), 5.26–5.09 (m, 1H, CH_2_), 4.60–4.40 (m, 2H, CH_2_), 3.51–2.99 (m, 4H, CH_2_), 1.26 (s, 9H, *t*Bu), 1.17 (s, 9H, *t*Bu), 1.14 (s, 9H, *t*Bu), 1.09 (s, 9H, *t*Bu), 0.90 (d, 9H, OPMe_3_) ppm; ^13^C-NMR (75 MHz, C_6_D_6_, 25 °C) δ: 170.10, 169.50, 169.35, 169.13 (C=O, CH=N), 161.74, 160.14 (Ar‒O), 140.44, 139.75, 139.73, 139.70, 139.52, 138.99, 132.66, 131.09, 129.60, 129.32 (Ar), 129.00 (Ph), 123.78 (Ph), 121.95, 121.73 (Ar), 120.18 (Ph), 62.63, 58.65, 39.39, 38.89 (CH_2_), 34.96, 35.83, 34.10 (2×) (q-*t*Bu), 31.44, 31.33, 29.65, 29.48 (*t*Bu), 17.65 (d, OPMe_3_) ppm; ^31^P{^1^H}-NMR (121 MHz, C_6_D_6_, 25 °C): 36.19 (OPMe_3_) ppm; IR (ATR, cm^−1^) ῦ: 3325 (br m, NH), 2957(m, C-H), 1653 (m, C=O), 1612(C=N, s), 1536 (m), 1255 (s), 1170 (m), 937 (m, O-O), 904 (s, Mo=O), 836 (s), 809 (m), 535 (w, Mo-O), 463 (m); EI-MS (100 V) *m*/*z*: 848.7 [M + H_2_O]^+^; Anal. Calcd. for C_48_H_62_MoN_4_O_7_∙OPMe_3_∙H_2_O: C, 60.47; H, 7.26; N, 5.54. Found: C, 59.77; H, 6.41; N, 5.31%. 

## 4. Conclusions

The reported synthesis of iminophenolate ligands equipped with pendant amidopropyl functionalities (**HL2** and **HL3**) allowed for the successful synthesis and isolation of mononuclear dioxidomolybdenum(VI) complexes [MoO_2_**L_2_**] (**2** and **3**). The here presented ligand design was successful in terms of an undesired cyclization of the ligand moieties, as previously observed with shorter amide substituents. More precisely, this cyclization reaction was prevented by introducing an additional methylene group between the imine and the amide functionality of the ligands. 

The resulting complexes **2** and **3** together with complex **1** featuring *n*-butyl side chains were reduced with PMe_3_ and subsequently exposed to molecular oxygen allowing the isolation of oxido peroxido complexes of the structure [MoO(O_2_)**L_2_**] (**9**–**11**). Thus, all complexes are capable of activating dioxygen in the desired fashion. The isolation of the reduced molybdenum(IV) intermediate was successful for the *n*-butyl ligand system, [MoO(PMe_3_)(**L1**)_2_] (**4**), while increased sensitivity of complexes **5** and **6** prevented their isolation in pure form. Instead, three polynuclear secondary products could be identified via single-crystal X-ray diffraction analysis.

Complexes **1**–**3** were found to be moderately active catalysts in the aerobic oxidation of PMe_3_ while cyclooctene and dimethyl sulfide were not oxidized. Although formation of a weak hydrogen bond to the peroxido moiety was observed, it has no beneficial influence on the reactivity. Nevertheless, the here presented investigation reveals that hydrogen bonds may preferentially be formed to the product OPMe_3_ rather than to the peroxido group thereby decreasing their expected beneficial effect. Therefore, systems that are more reactive will have to consider less flexible substituents leading to stronger hydrogen bonds directed towards the peroxido oxygen atoms.

## Figures and Tables

**Table 1:** Selected bond lengths (Å) and angles (°) for the dioxidomolybdenum(VI) complexes **1**–**3**.

	1	2	3
Mo1–O1	1.705(3)	1.7078(10)	1.708(3)
Mo1–O2	1.712(3)	-	-
Mo1–O11	1.952(3)	1.9571(10)	1.950(2)
Mo1–O21	1.955(3)	-	-
Mo1–N17	2.351(2)	2.3548(12)	2.333(3)
Mo1–N27	2.341(4)	-	-
O1–Mo1–O2	106.80(16)	107.62(7)	108.0(2)
O11-Mo1-O21	160.48(11)	157.63(6)	159.49(16)
N17-Mo1-N27	73.30(12)	73.42(6)	74.78(16)

**Table 2:** Selected bond lengths (Å) and angles (°) for the oxido peroxido molybdenum(VI) complexes **9**, **10** and **11**·2OPMe_3._

	9	10	11·2OPMe_3_
Mo1–O1	1.6817(18)	1.6959(13)	1.698(3)
Mo1–O2	1.9573(19)	1.9398(13)	1.961(4)
Mo1-O3	1.9469(16)	1.9651(14)	1.945(3)
Mo1–O11	2.0353(10)	2.0256(13)	2.014(3)
Mo1–O21	2.0113(10)	2.0283(12)	2.036(3)
Mo1–N17	2.1670(14)	2.1542(15)	2.167(4)
Mo1–N27	2.2053(14)	2.1818(15)	2.189(4)
O2–O3	1.431(3)	1.4426(18)	1.406(4)
O1–Mo1–O2	101.97(9)	100.50(6)	99.89(15)
O1–Mo1–O3	99.60(8)	98.18(6)	98.13(14)
O2–Mo1–O3	43.01(7)	43.35(5)	42.21(13)
O11-Mo1-O21	80.51(4)	81.56(5)	82.21(12)
N17-Mo1-N27	159.62(5)	161.89(6)	161.32(13)

**Table 3:** Yield after 24 h in the aerobic oxidation of PMe_3_.

Product	-	1	2	3
OPMe_3_	<5%	46%	53%	23%
OP(OMe)Me_2_	-	-	4%	2%

Conditions: 1 mol% catalyst, rt, C_6_D_6,_ excess O_2_.

## Supplementary Materials

The supplementary materials are available online.
