# Supplementary material for: Dioxygen Activation with Molybdenum Complexes Bearing Amide-Functionalized Iminophenolate Ligands
Source: Molecules. 2019 May 10;24(9):1814. doi: 10.3390/molecules24091814 (PMC6539658; doi:10.3390/molecules24091814)
Supplement: Supplementary file 1 [file molecules-24-01814-s001.pdf]

Dioxygen Activation with Molybdenum Complexes Bearing Amide-Functionalized Iminophenolate Ligands

Niklas Zwettler,[1] Madeleine A. Ehweiner,[1] Jörg A. Schachner,[1] Antoine Dupé,[1] Ferdinand Belaj[1] and Nadia C. Mösch-Zanetti*[1]

[1]Institute of Chemistry, Inorganic Chemistry, University of Graz, Schubertstrasse 1, 8010 Graz, Austria

E-mail: nadia.moesch@uni-graz.at

Supplementary Material

Content

Additional Figures and Schemes 5

Figure S1. Comparison of the ^{1}H NMR shifts of the α-methylene and amide protons of HL2 and the corresponding C1 analogue.[1] 5

Figure S2. Molecular view (50% probability level) of ligand HL3. Solvent molecules and H-atoms (except O and N-bound) are omitted for clarity 5

Figure S3. Molecular view (50% probability level) of the mixed ligand compound 3^{acac}. Solvent molecules and H-atoms (except amide bound) are omitted for clarity. 6

Figure S4. Molecular view (50% probability level) of the dimeric compound 7. Solvent molecules, H-atoms as well as aryl-bound *t*Bu groups omitted for clarity 6

Figure S5. Left: Molecular view (50% probability level) of a single molybdenum center of compound 8. H-atoms are omitted for clarity; Right: Molecular view (50% probability level) of the tetranuclear molybdenum core found in compound 8. Ligand atoms except first coordination sphere around Mo and their connectivity are omitted for clarity. 7

Figure S6. Molecular structure of compound 12. Solvent molecules and H-atoms (except N-bound) are omitted for clarity reasons. 7

NMR Spectra of the Compounds 8

Figure S7. ^{1}H NMR spectrum of ligand HL1[2] in C_6D_6 8

Figure S8. ^{13}C NMR spectrum of of ligand HL1[2] in C_6D_6. 8

Figure S9. ^{1}H NMR spectrum of ligand HL2 in C_6D_6. 9

Figure S10. ^{13}C NMR spectrum of of ligand HL2 in C_6D_6. 9

Figure S11. ^{1}H NMR spectrum of ligand HL3 in C_6D_6. 10

Figure S12. ^{13}C NMR spectrum of ligand HL3 in C_6D_6 10

Figure S13. ^{1}H NMR spectrum of complex 1[3] (2 isomers) in C_6D_6. 11

Figure S14. ^{13}C NMR spectrum of complex 1[3] (2 isomers) in C_6D_6 11

Figure S15. ^{1}H NMR spectrum of complex 2 (2 isomers) in C_6D_6. 12

Figure S16. ^{13}C NMR spectrum of complex 2 (2 isomers) in C_6D_6. 12

Figure S17. ^{1}H NMR spectrum of complex 3 (2 isomers) in C_6D_6. 13

Figure S18. ^{13}C NMR spectrum of complex 3 (2 isomers) in C_6D_6. 13

Figure S19. ^{1}H NMR spectrum of complex 4 (2 isomers) in C_6D_6. 14

Figure S20. ^{13}C NMR spectrum of complex 4 (2 isomers) in C_6D_6. 14

Figure S21. ^{31}P NMR spectrum of complex 4 (2 isomers) in C_6D_6 15

Figure S22. ^{1}H NMR spectrum of a mixture of complex 5 (2 isomers), PMe_3 and $OPMe_3$ in C_6D_6 15

Figure S23. ^{13}C NMR spectrum of a mixture of complex 5 (2 isomers), PMe_3 and $OPMe_3$ in C_6D_6. 16

Figure S24. ^{31}P NMR spectrum of a mixture of complex 5 (2 isomers), PMe_3 and $OPMe_3$ in C_6D_6. 16

Figure S25. ^{1}H NMR spectrum of complex 9 in C_6D_6. 17

Figure S26. ^{13}C NMR spectrum of complex 9 in C_6D_6. 17

Figure S27. ^{1}H NMR spectrum of complex 10 in C_6D_6. 18

Figure S28. ^{13}C NMR spectrum of complex 10 in C_6D_6. 18

Figure S29. ^{1}H NMR spectrum of compound 11·$OPMe_3$ in C_6D_6. 19

Figure S30. ^{13}C NMR spectrum of compound 11·$OPMe_3$ in C_6D_6. 19

X-Ray single Crystal Structure Determination of HL3, 1-3, 3^{acac}, 7-10, 10·$OPMe_3$, 11·$OPMe_3$, 11·2$OPMe_3$ and 12 20

Table S1. Selected Crystallographic Data and Structure Refinement for HL3, 2, 3, 3^{acac}, 7 and 8. 20

Table S2. Selected Crystallographic Data and Structure Refinement for 9, 10, 10·$OPMe_3$, 11·$OPMe_3$, 11·2$OPMe_3$ and 12. 21

Crystal Structure Determinations – General 22

Crystal Structure Determination – Additional Refinement Informations 22

Figure S31. Stereoscopic ORTEP plot of **HL3** 24

Table S3. Hydrogen bonds [Å, °] for **HL3**. 25

Figure S32. Stereoscopic ORTEP plot of **1** 25

Table S4. Selected bond lengths [Å] and angles [°] for **1**. 25

Table S5. Hydrogen bond [Å, °] for **1**. 26

Figure S33. Stereoscopic ORTEP plot of **2** 26

Table S6. Selected bond lengths [Å] and angles [°] for **2**. 26

Table S7. Hydrogen bond [Å, °] for **2**. 27

Figure S34. Stereoscopic ORTEP plot of **3**. 28

Table S8. Selected bond lengths [Å] and angles [°] for **3**. 28

Table S9. Hydrogen bonds [Å, °] for **3** 29

Figure S35. Stereoscopic ORTEP plot of $\mathbf{3^{acac}}$. 29

Table S10. Selected bond lengths [Å] and angles [°] for $\mathbf{3^{acac}}$. 29

Table S11. Hydrogen bond [Å, °] for $\mathbf{3^{acac}}$. 30

Figure S36. Stereoscopic ORTEP plot of **7**. 30

Table S12. Selected bond lengths [Å] and angles [°] for **7**. 31

Table S13. Hydrogen bonds [Å, °] for **7**. 31

Figure S37. Stereoscopic ORTEP plot of the asymmetric unit of **8**. 32

Figure S38. Stereoscopic ORTEP plot of the tetrameric complex of **8**. 32

Table S14. Selected bond lengths [Å] and angles [°] for **8**. 33

Table S15. Hydrogen bond [Å, °] for **8**. 33

Figure S39. Stereoscopic ORTEP plot of **9**. 34

Table S16. Selected bond lengths [Å] and angles [°] for **9**. 34

Figure S40. Stereoscopic ORTEP plot of **10**. 35

Table S17. Selected bond lengths [Å] and angles [°] for **10** 35

Table S18. Hydrogen bonds [Å, °] for **10** 36

Figure S41. Stereoscopic ORTEP plot of the metal complex of **10·OPMe$_3$**. 36

Figure S42. Stereoscopic ORTEP plot of two metal complexes of **10·OPMe$_3$** together with two trimethylphosphine molecules. 37

Table S19. Selected bond lengths [Å] and angles [°] for **10·OPMe$_3$** 37

Table S20. Hydrogen bonds [Å, °] for **10·OPMe$_3$**. 38

Figure S43. Stereoscopic ORTEP plot of **11·OPMe$_3$**. 38

Table S21. Selected bond lengths [Å] and angles [°] for **11·OPMe$_3$** 38

Table S22. Hydrogen bonds [Å, °] for **11·OPMe$_3$**. 39

Figure S44. Stereoscopic ORTEP plot of **11·2OPMe$_3$**. 39

Table S23. Selected bond lengths [Å] and angles [°] for **11·2OPMe$_3$** 40

Table S24. Hydrogen bonds [Å, °] for **11·2OPMe$_3$**. 40

Figure S45. Stereoscopic ORTEP plot of **12**. 41

Table S25. Selected bond lengths [Å] and angles [°] for **12**. 41

Table S26. Hydrogen bonds [Å, °] for **12**. 42

References 43

Additional Figures and Schemes

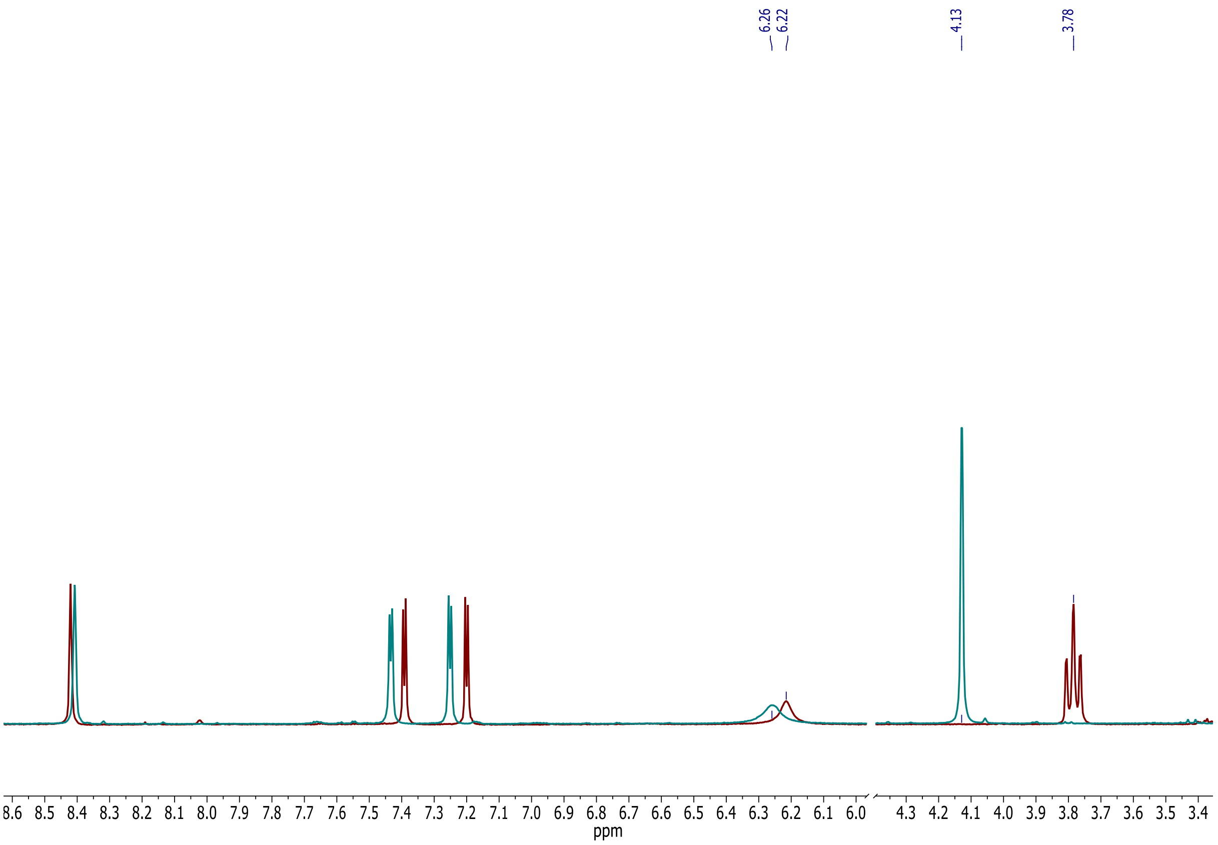

Figure S1. Comparison of the ^{1}H NMR shifts of the α-methylene and amide protons of **HL2** and the corresponding C1 analogue[1].

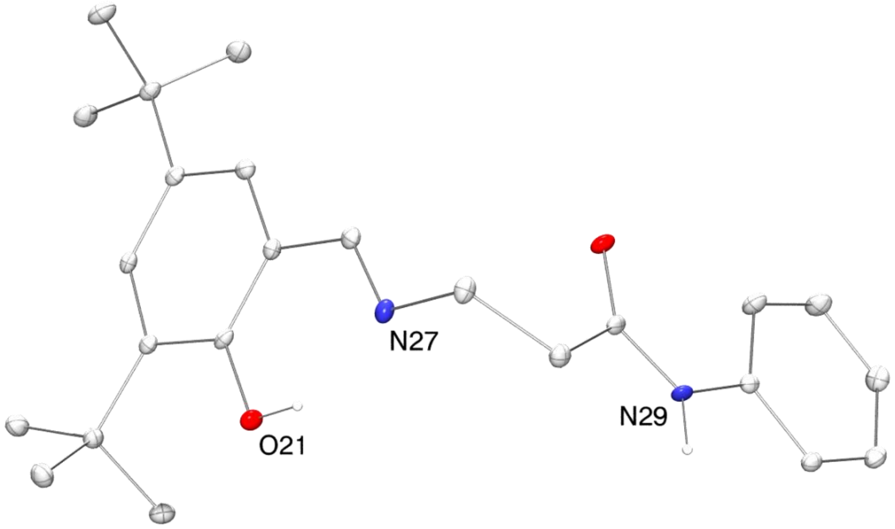

Figure S2. Molecular view (50% probability level) of ligand **HL3**. Solvent molecules and H-atoms (except O and N-bound) are omitted for clarity.

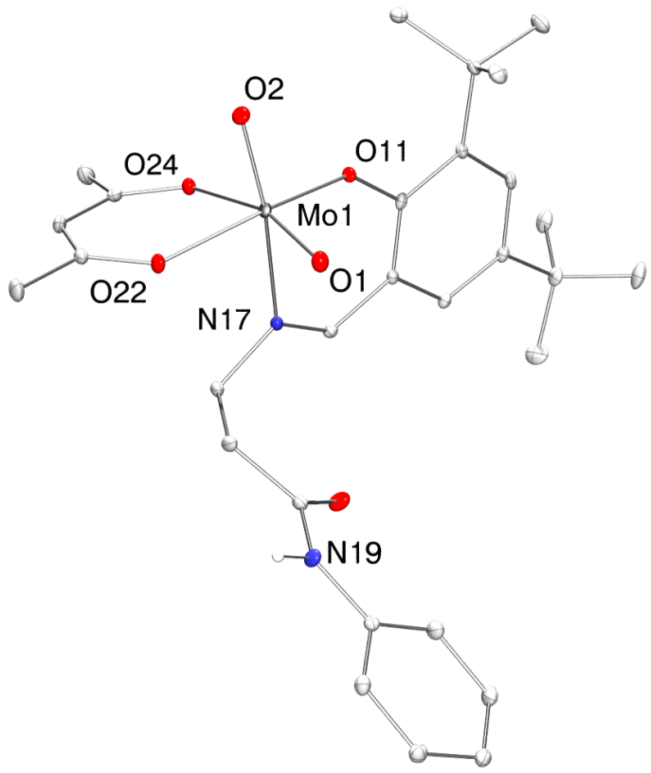

Figure S3. Molecular view (50% probability level) of the mixed ligand compound **3**[acac]. Solvent molecules and H-atoms (except amide bound) are omitted for clarity.

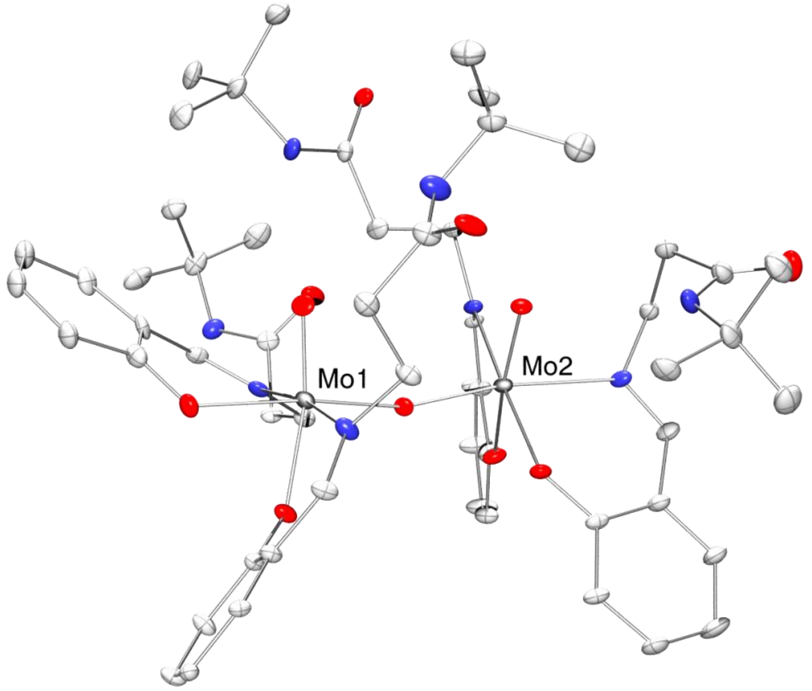

Figure S4. Molecular view (50% probability level) of the dimeric compound **7**. Solvent molecules, H-atoms as well as aryl-bound *t*Bu groups omitted for clarity.

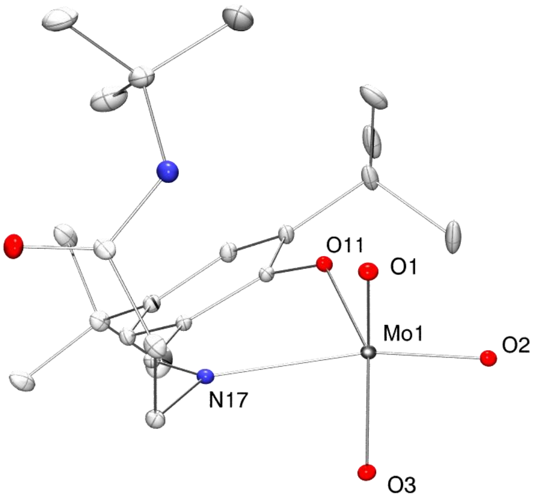

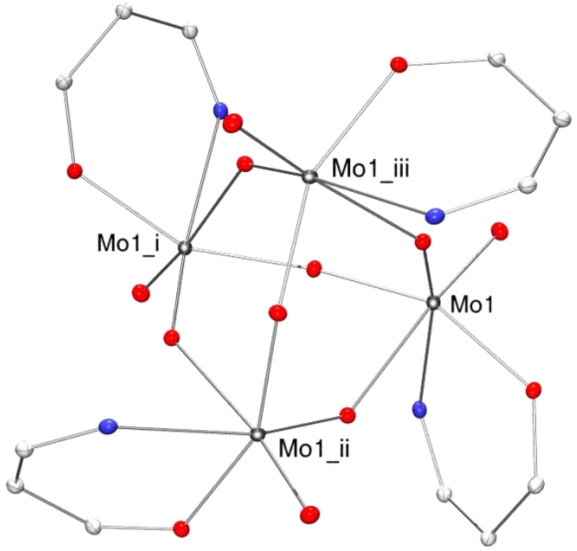

Figure S5. Left: Molecular view (50% probability level) of a single molybdenum center of compound **8**. H-atoms are omitted for clarity; Right: Molecular view (50% probability level) of the tetranuclear molybdenum core found in compound **8**. Ligand atoms except first coordination sphere around Mo and their connectivity are omitted for clarity.

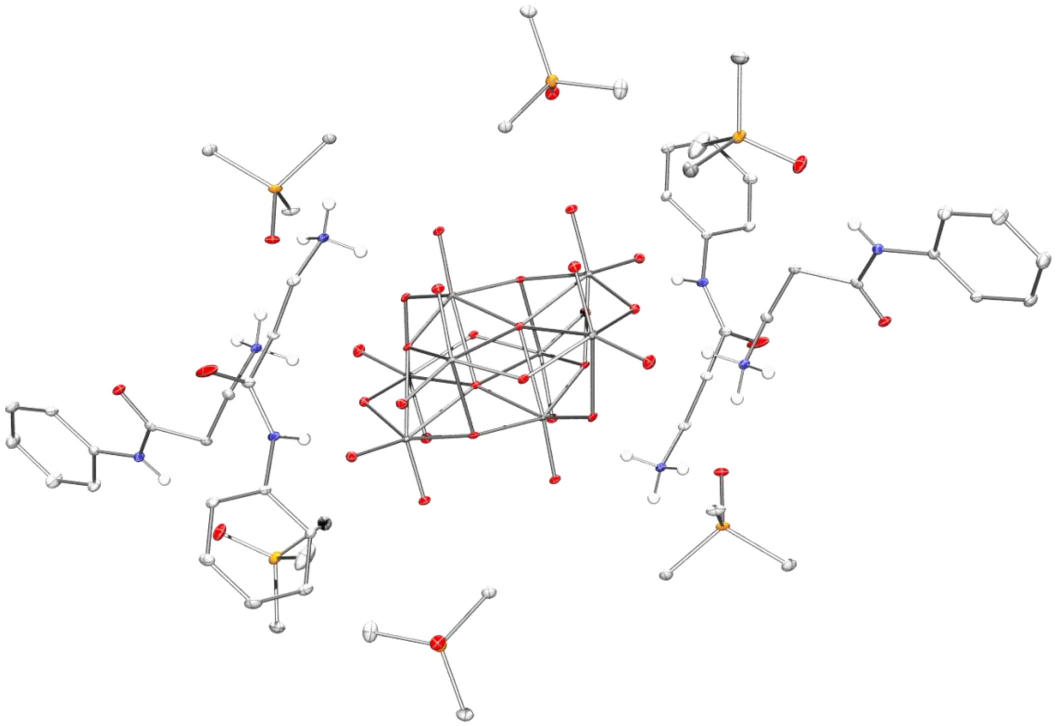

Figure S6. Molecular structure of compound **12**. Solvent molecules and H atoms (except N-bound) are omitted for clarity reasons.

NMR Spectra of the Compounds

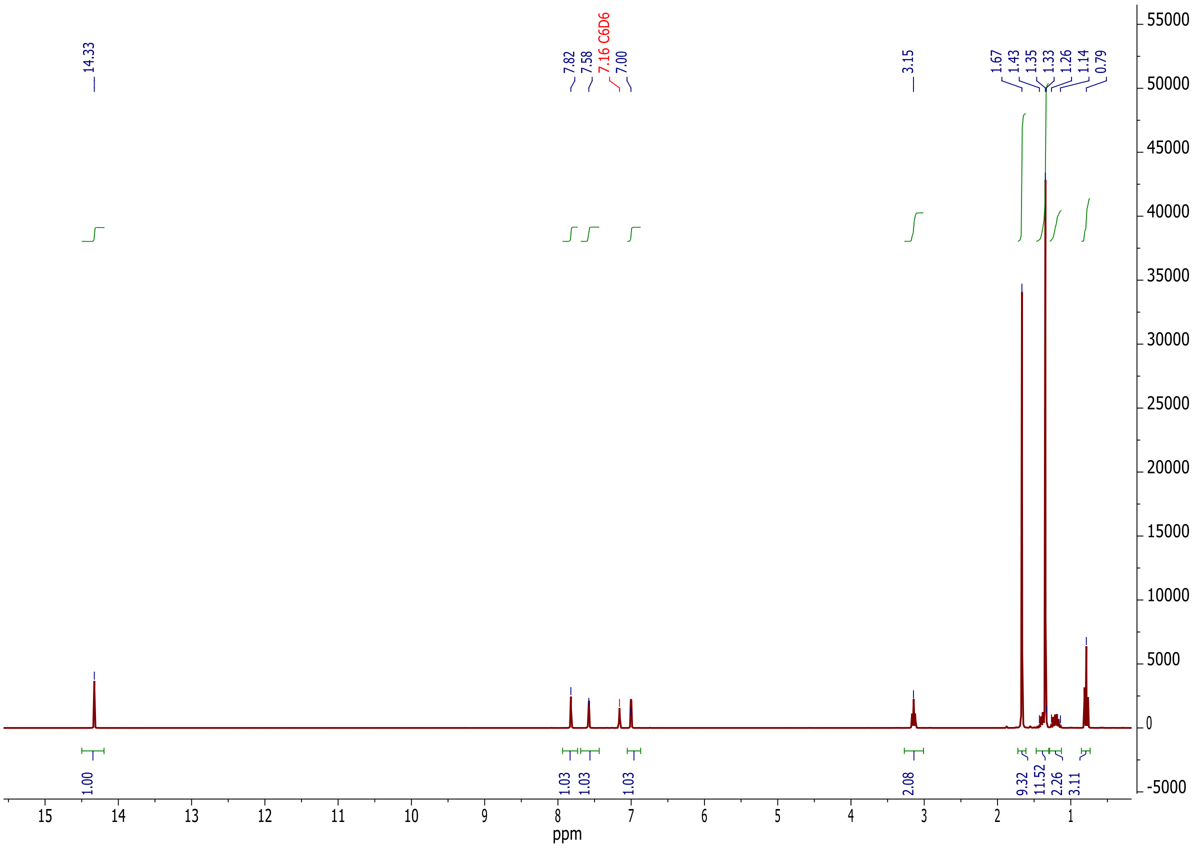

Figure S7. ^{1}H NMR spectrum of ligand **HL1**[2] in C_6D_6.

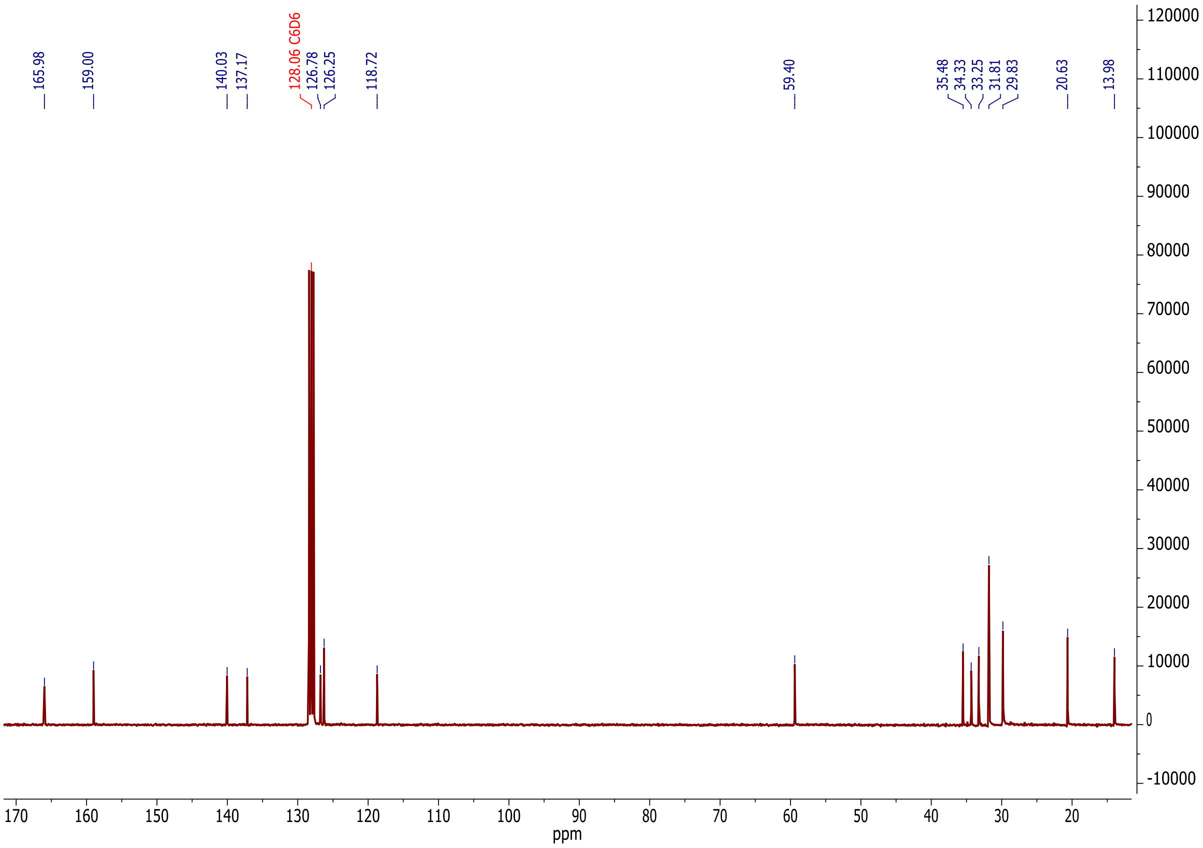

Figure S8. ^{13}C NMR spectrum of of ligand **HL1**[2] in C_6D_6.

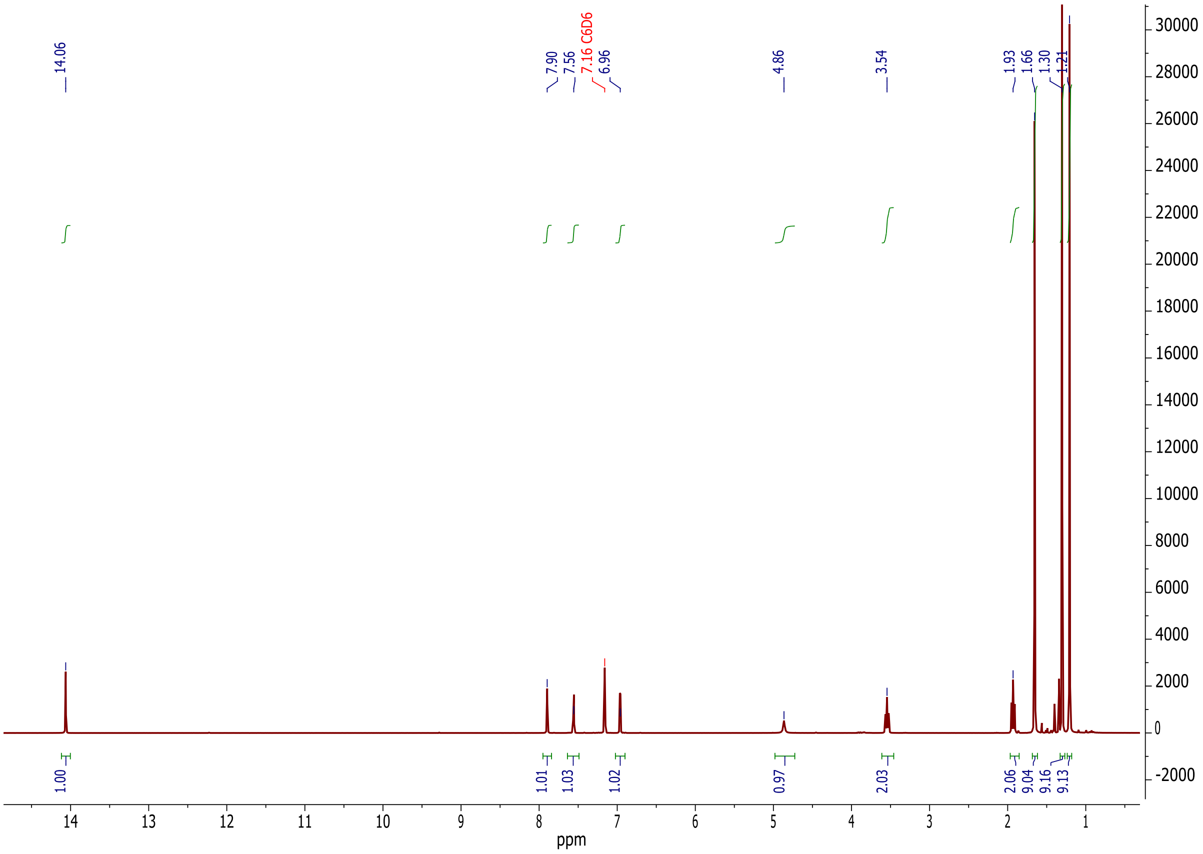

Figure S9. ^{1}H NMR spectrum of ligand **HL2** in C_6D_6.

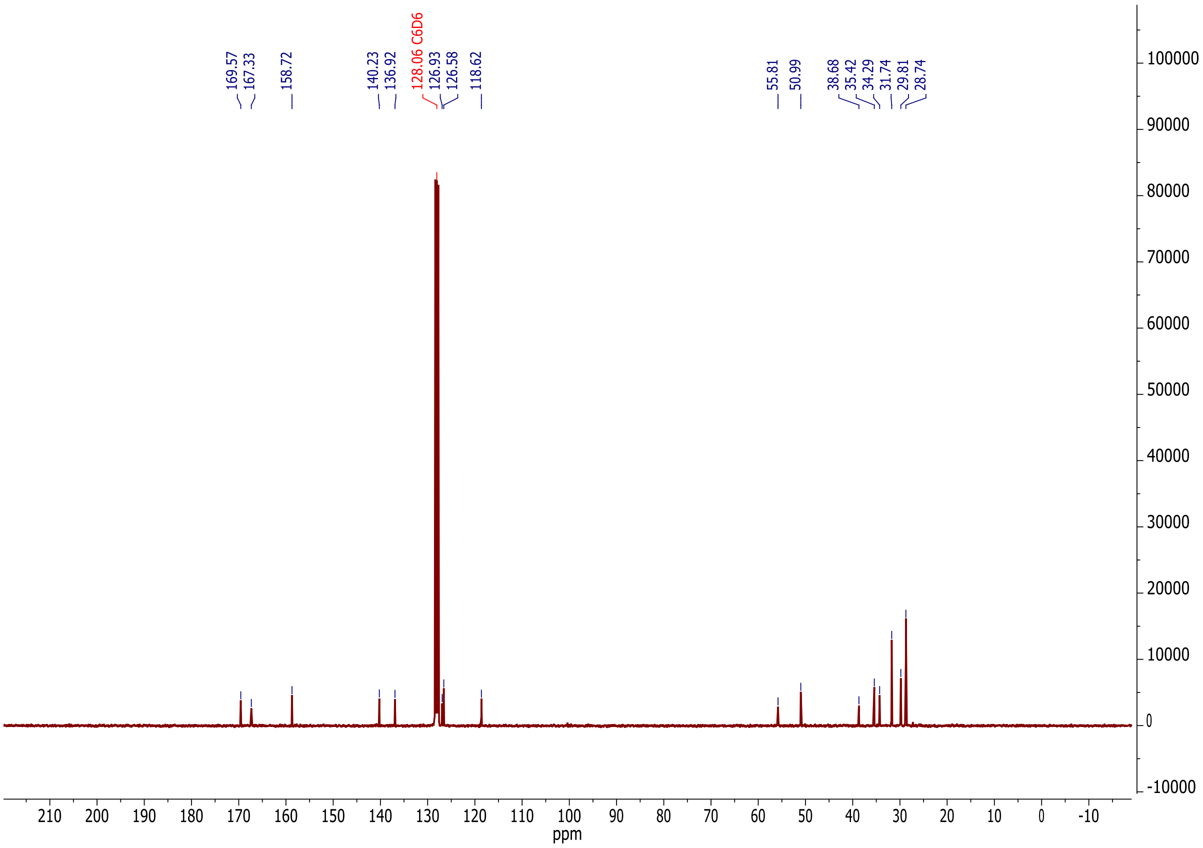

Figure S10. ^{13}C NMR spectrum of of ligand **HL2** in C_6D_6.

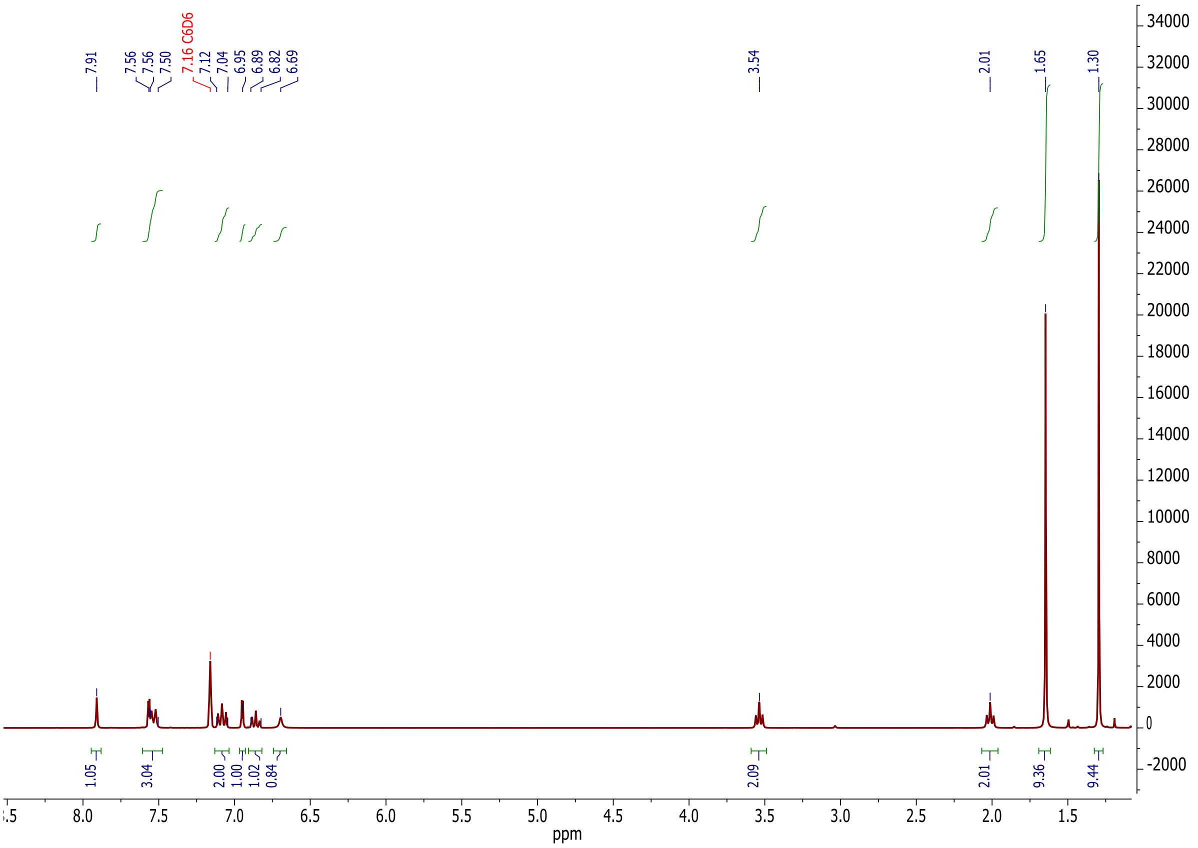

Figure S11. ^{1}H NMR spectrum of ligand **HL3** in C_6D_6.

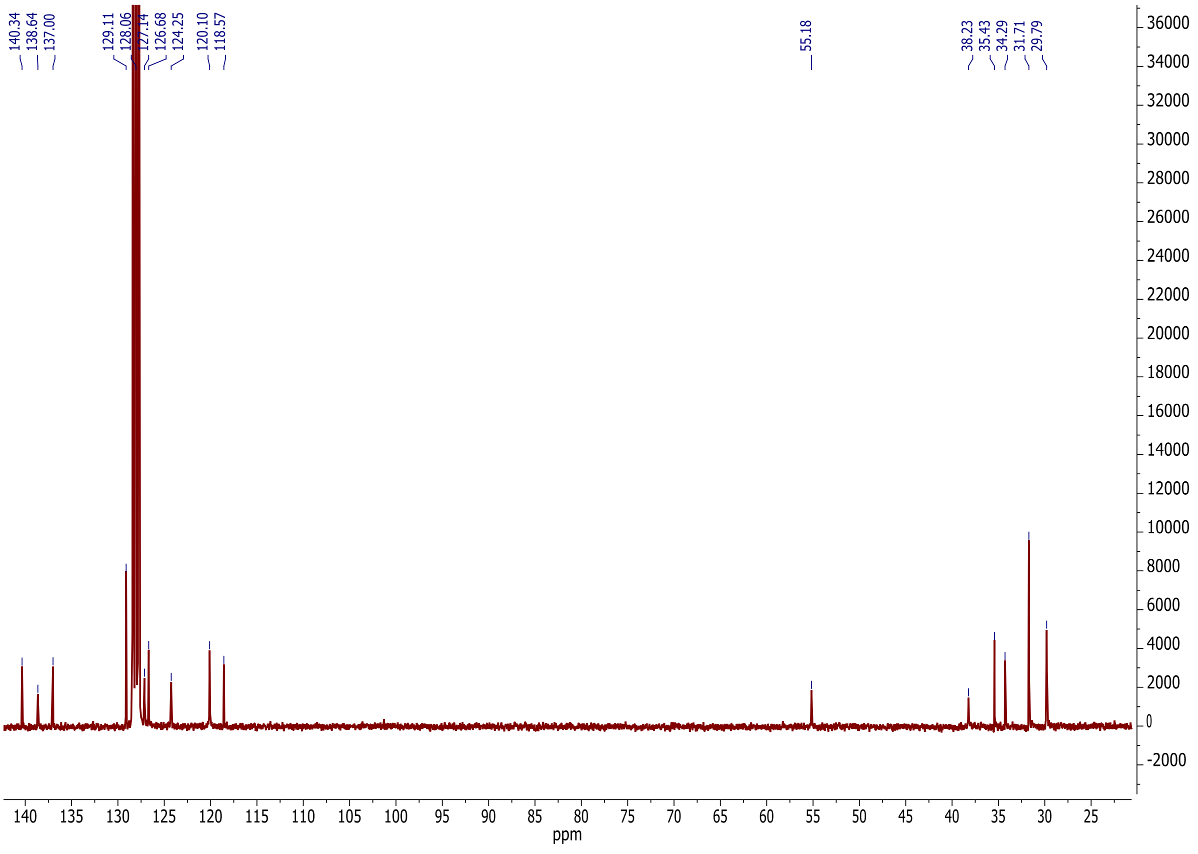

Figure S12. ^{13}C NMR spectrum of ligand **HL3** in C_6D_6.

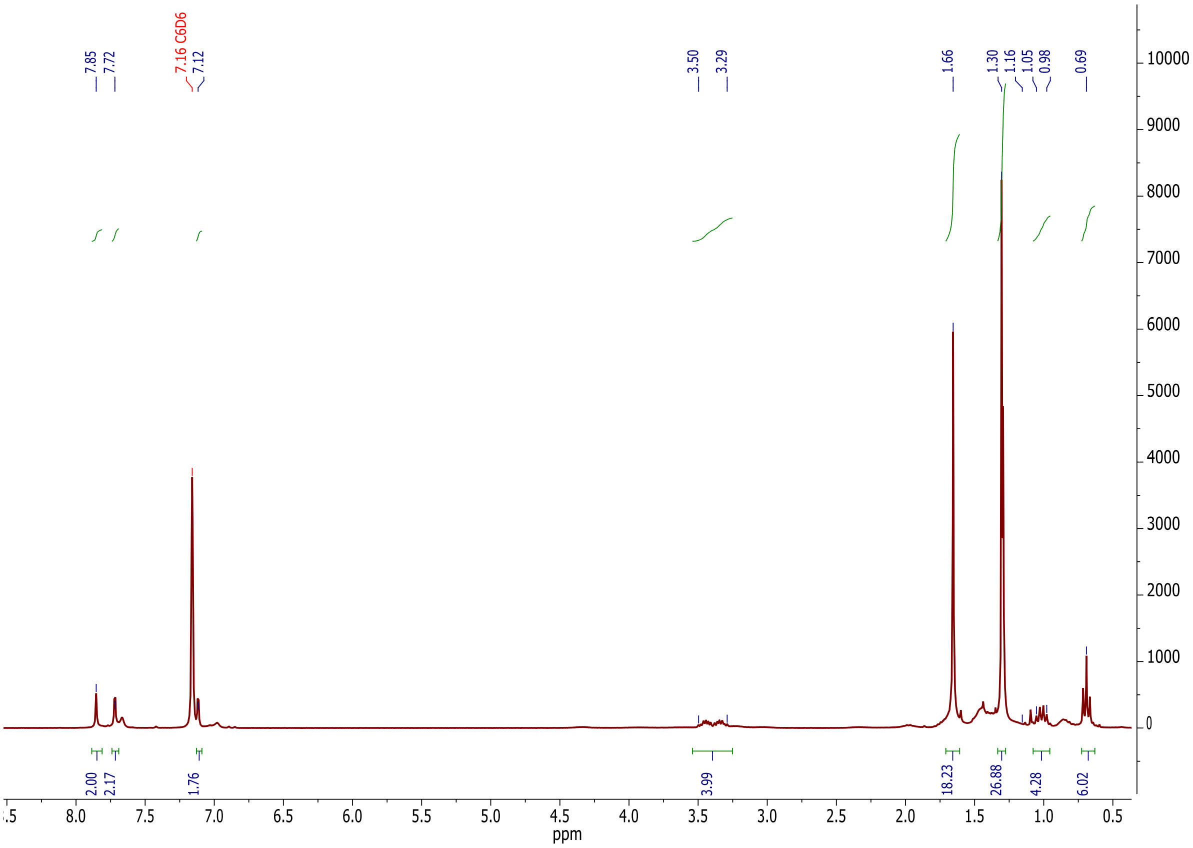

Figure S13. ^{1}H NMR spectrum of complex **1**[3] (2 isomers) in C_6D_6.

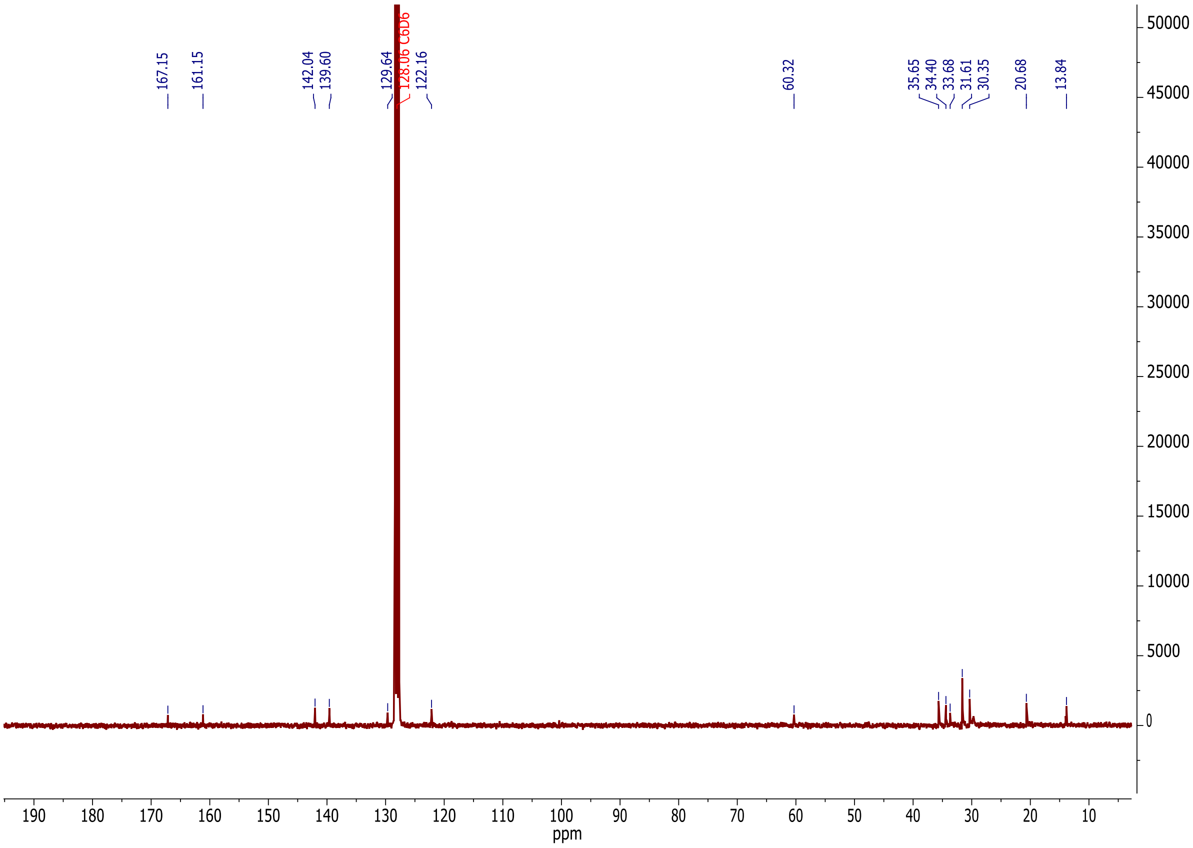

Figure S14. ^{13}C NMR spectrum of complex **1**[3] (2 isomers) in C_6D_6.

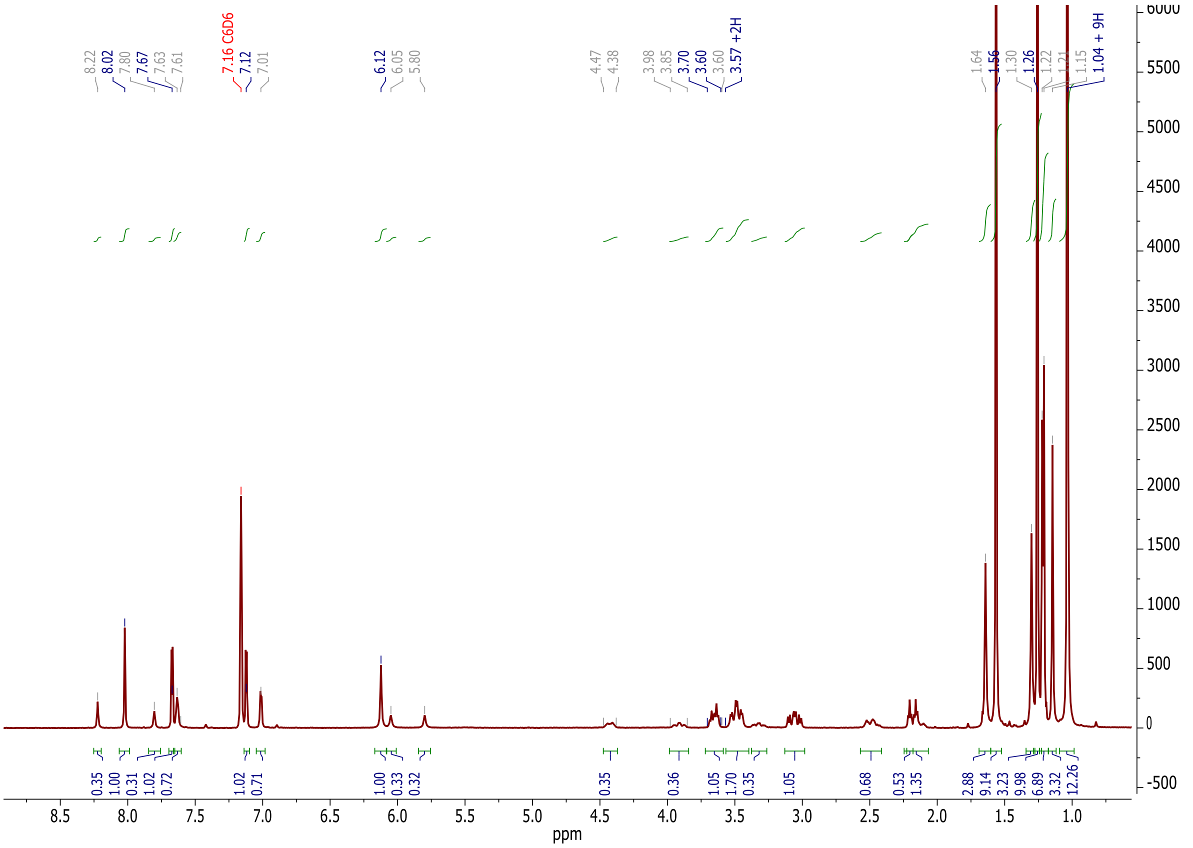

Figure S15. ^{1}H NMR spectrum of complex **2** (2 isomers) in C_6D_6.

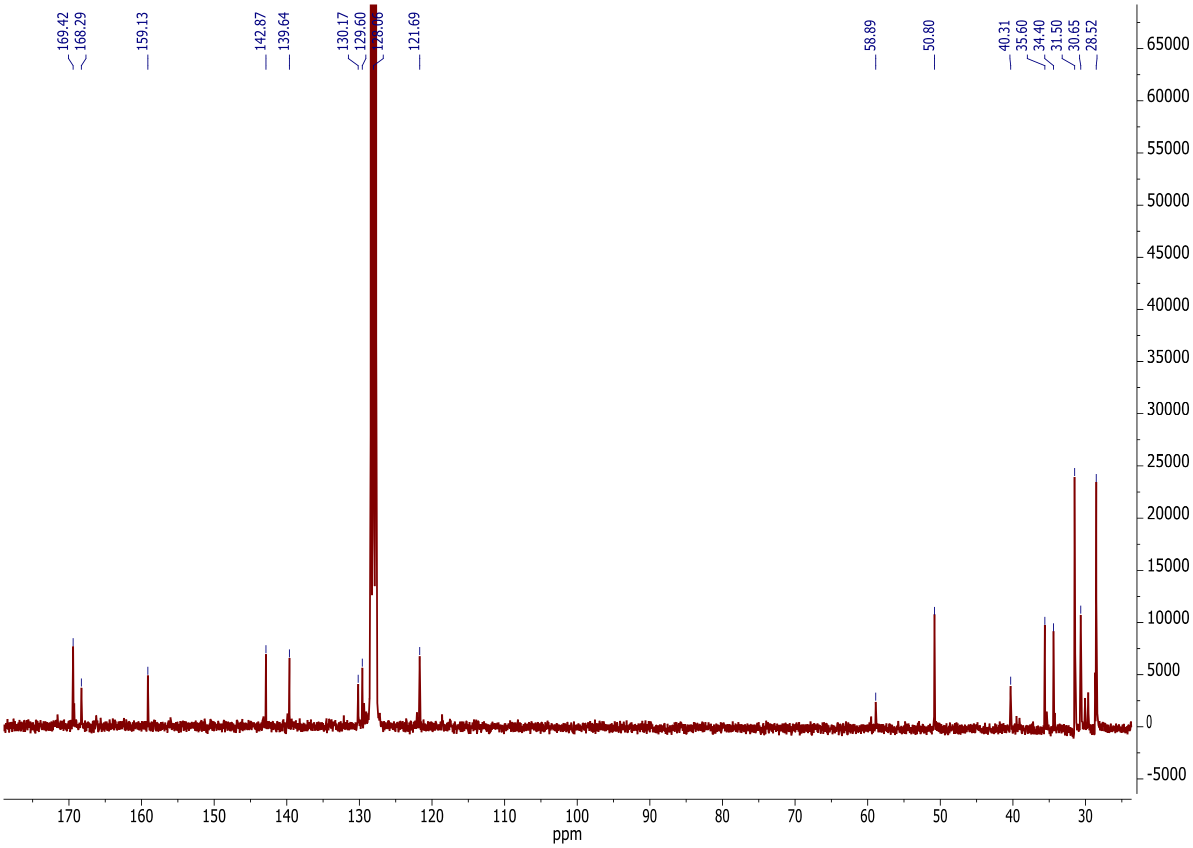

Figure S16. ^{13}C NMR spectrum of complex **2** (2 isomers) in C_6D_6.

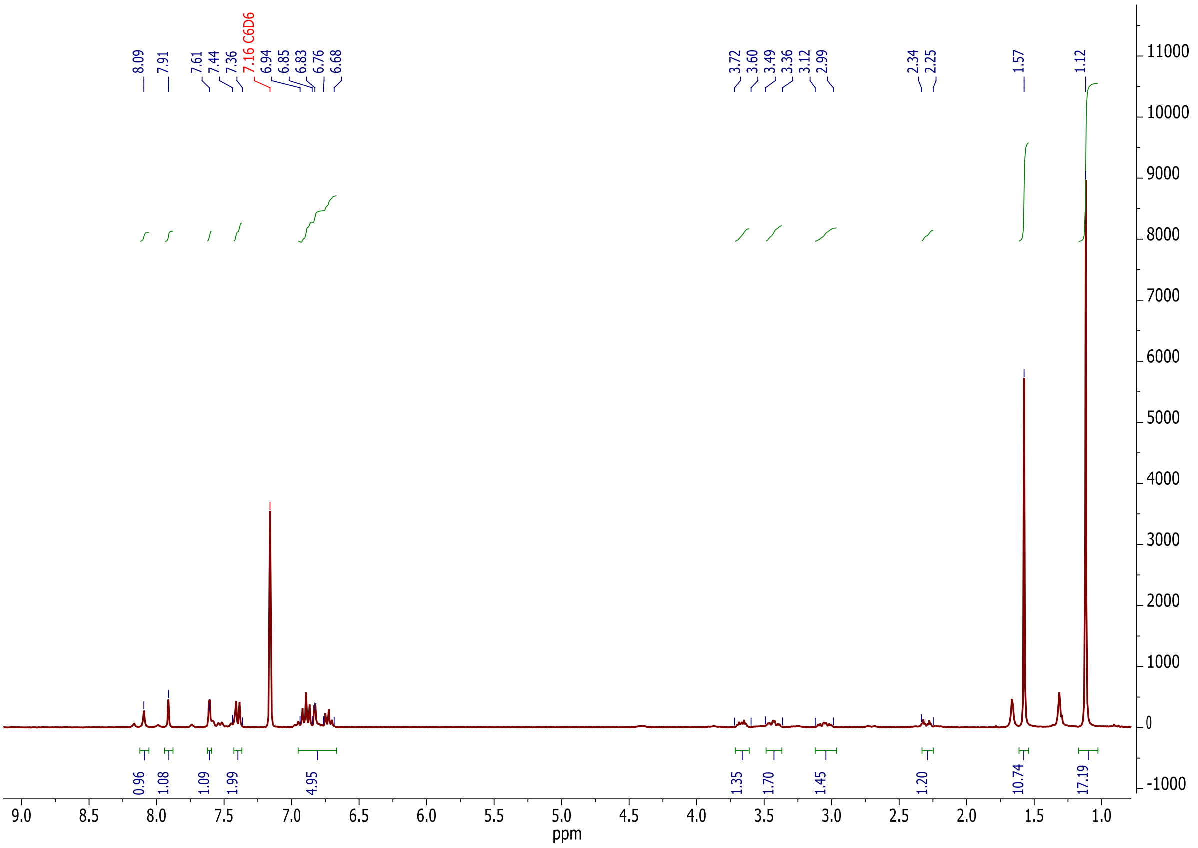

Figure S17. ^{1}H NMR spectrum of complex **3** (2 isomers) in C_6D_6.

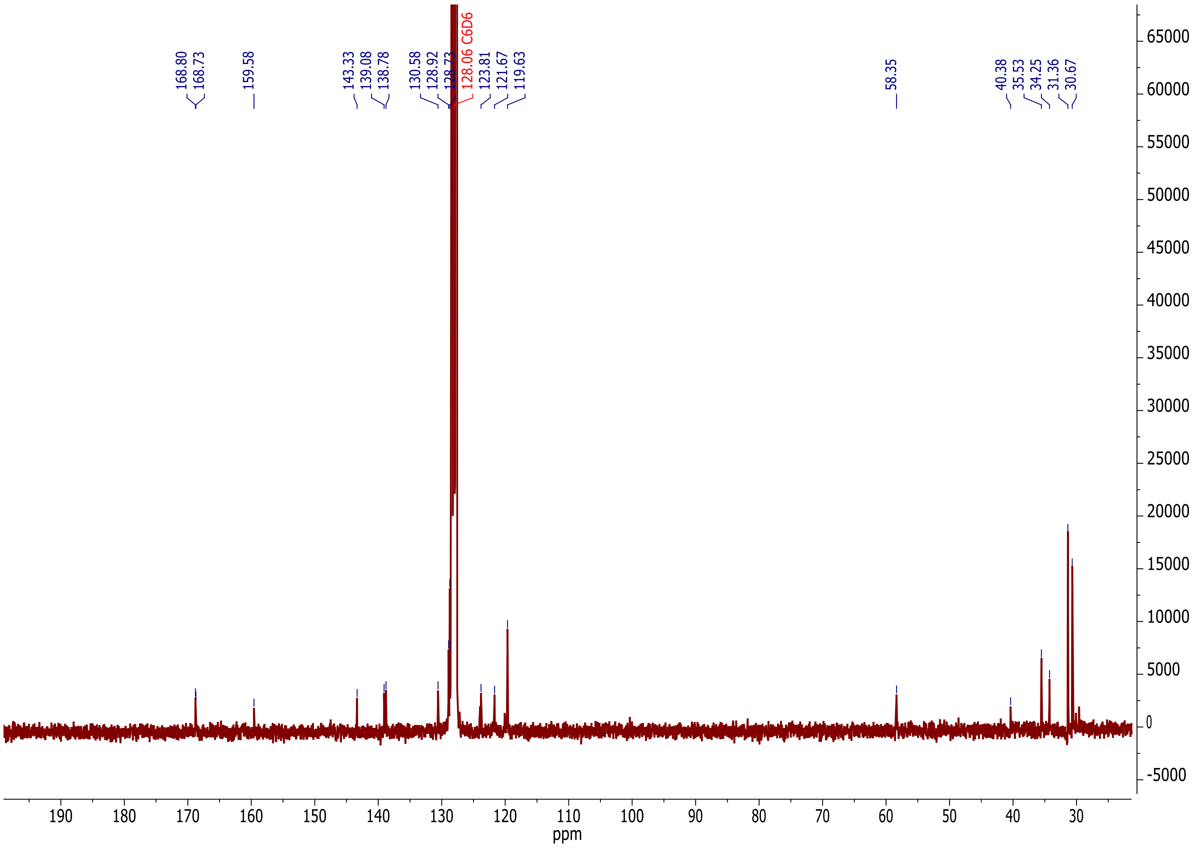

Figure S18. ^{13}C NMR spectrum of complex **3** (2 isomers) in C_6D_6.

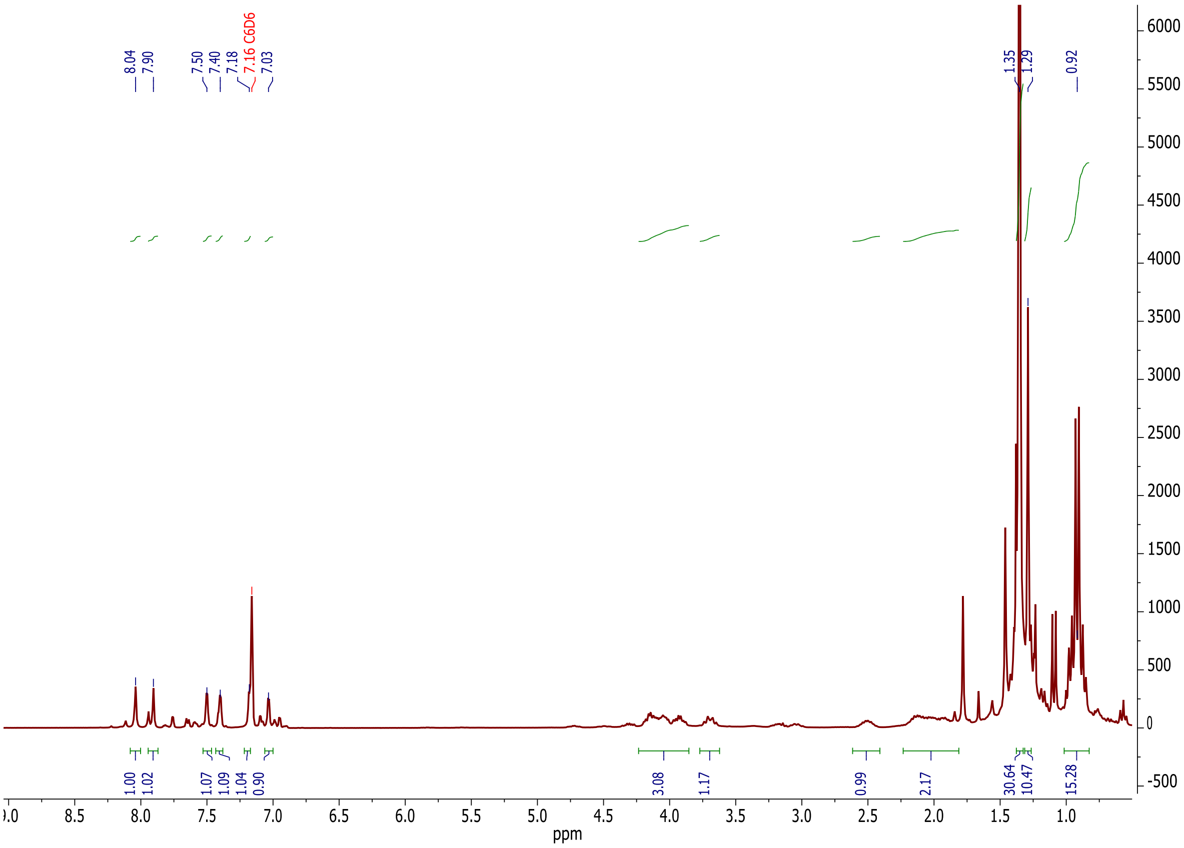

Figure S19. ^{1}H NMR spectrum of complex **4** (2 isomers) in C_6D_6.

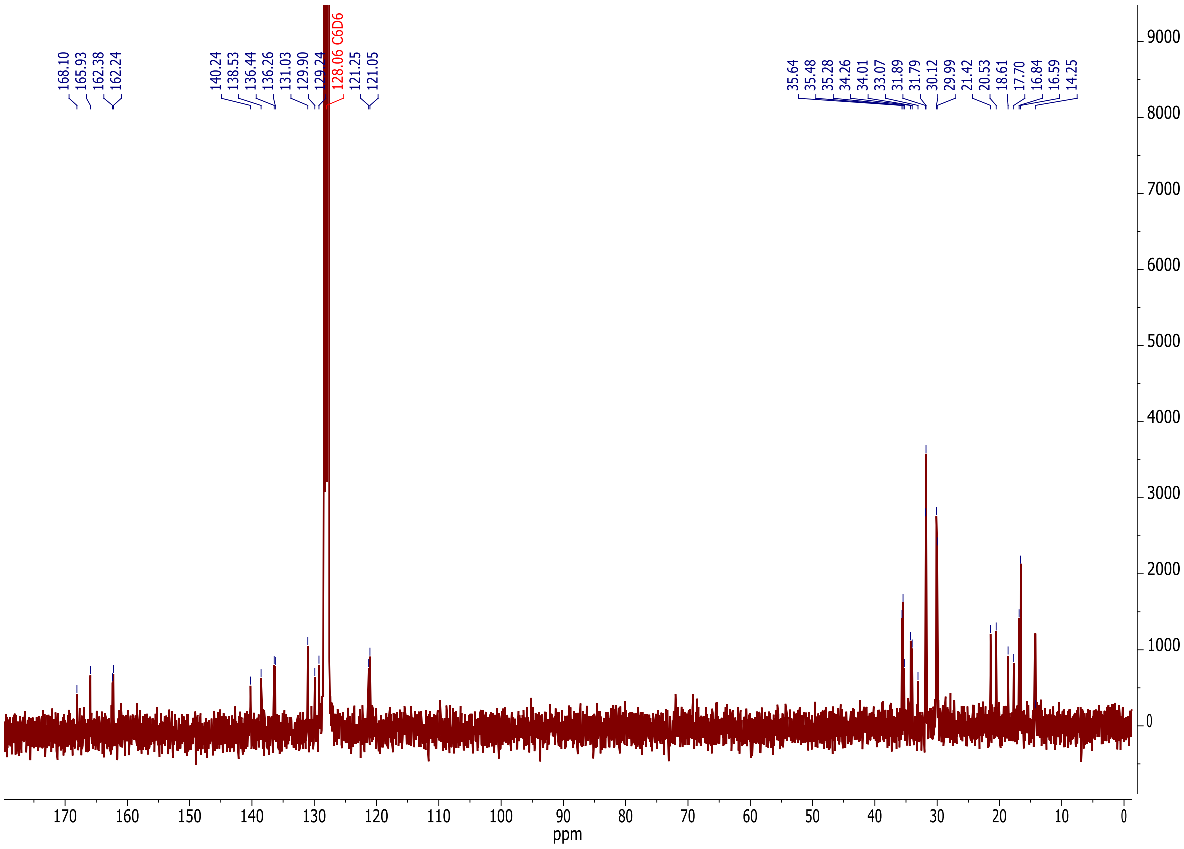

Figure S20. ^{13}C NMR spectrum of complex **4** (2 isomers) in C_6D_6.

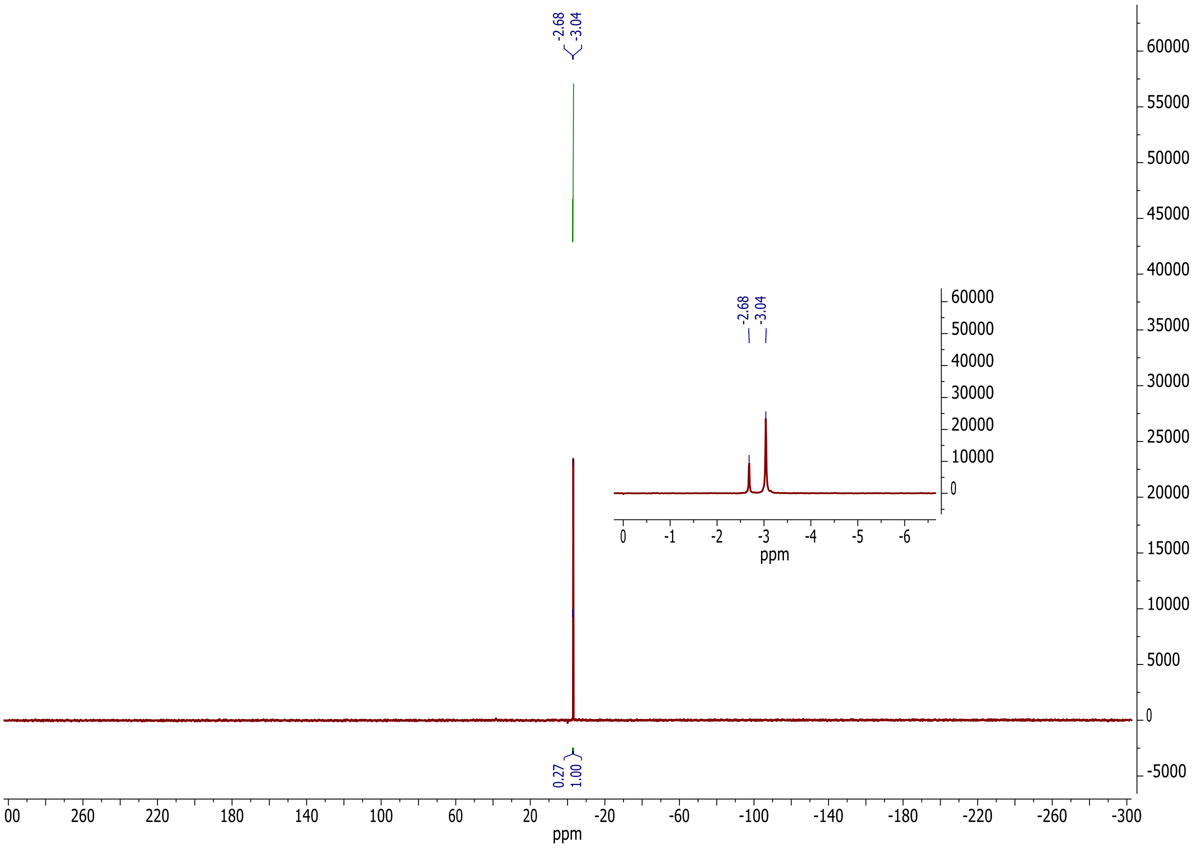

Figure S21. ^{31}P NMR spectrum of complex **4** (2 isomers) in C_6D_6.

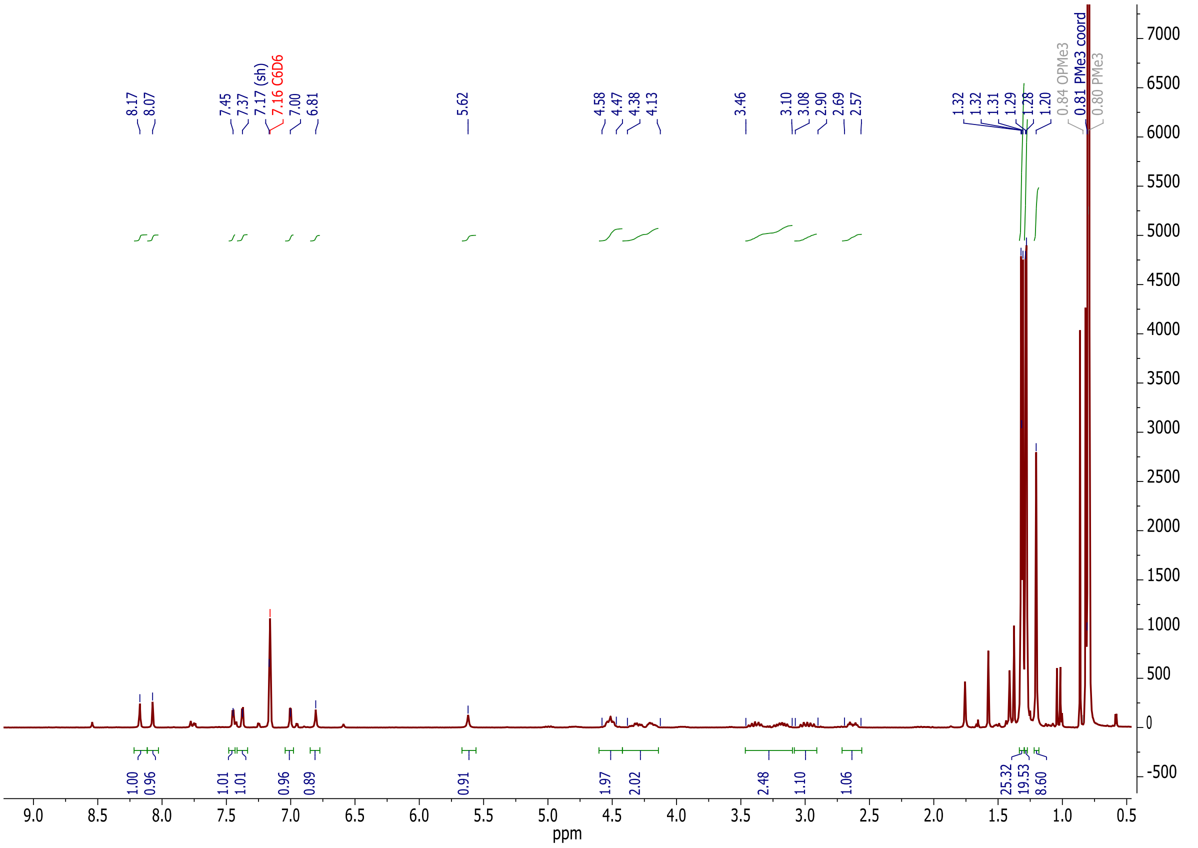

Figure S22. ^{1}H NMR spectrum of a mixture of complex **5** (2 isomers), PMe_3 and $OPMe_3$ in C_6D_6.

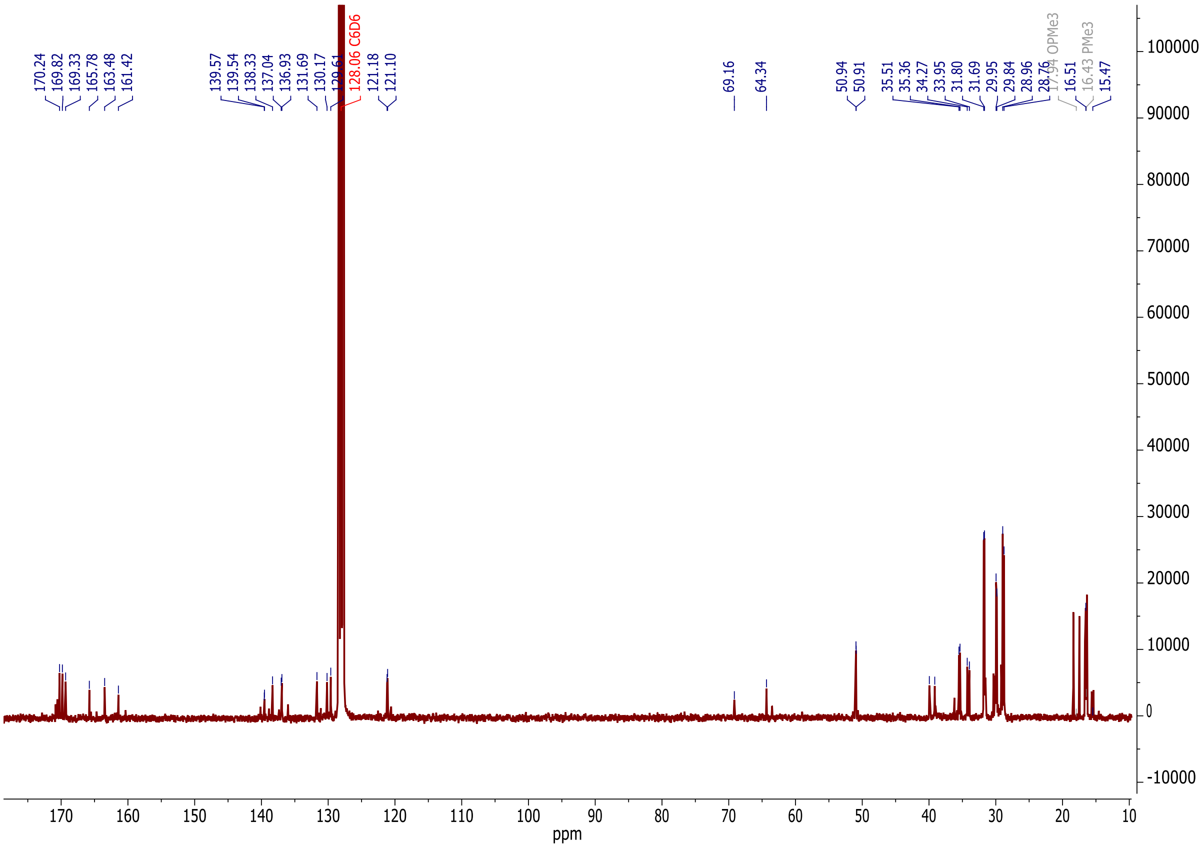

Figure S23. ^{13}C NMR spectrum of a mixture of complex **5** (2 isomers), PMe_3 and $OPMe_3$ in C_6D_6.

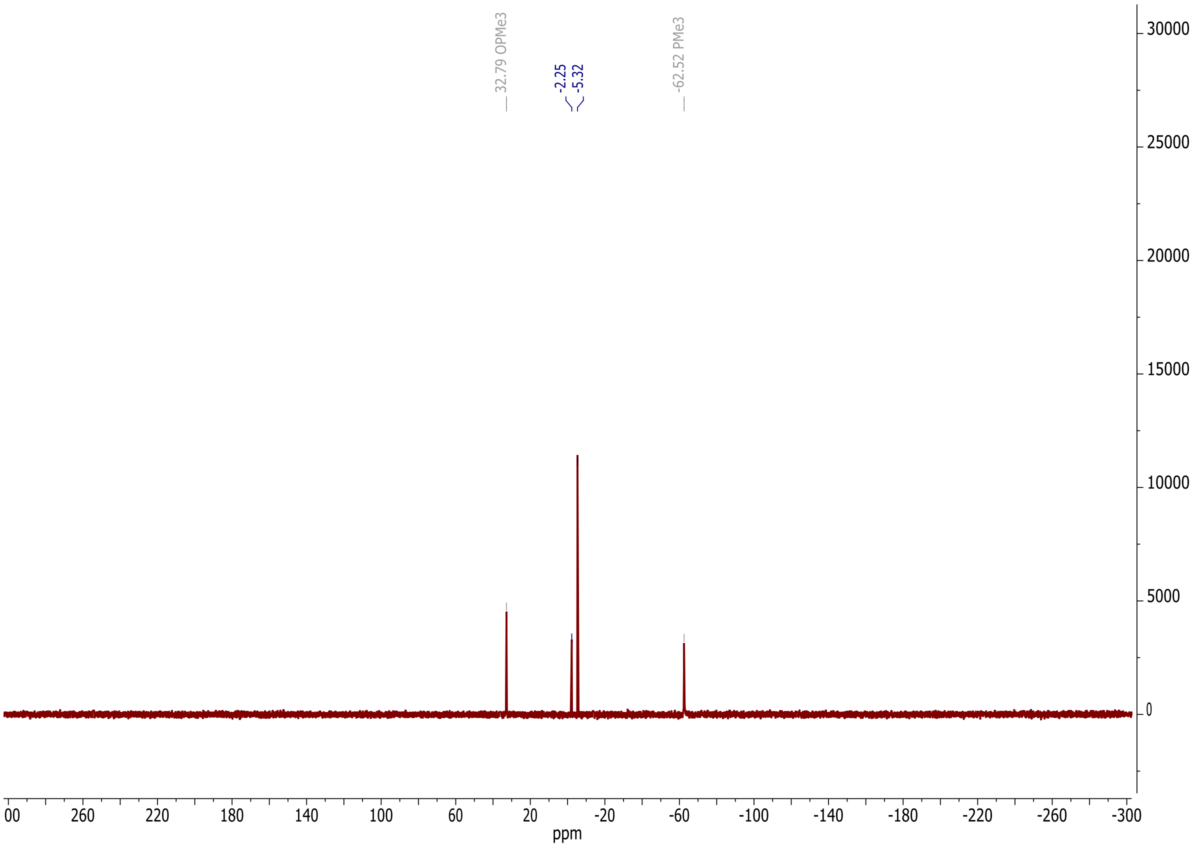

Figure S24. ^{31}P NMR spectrum of a mixture of complex **5** (2 isomers), PMe_3 and $OPMe_3$ in C_6D_6.

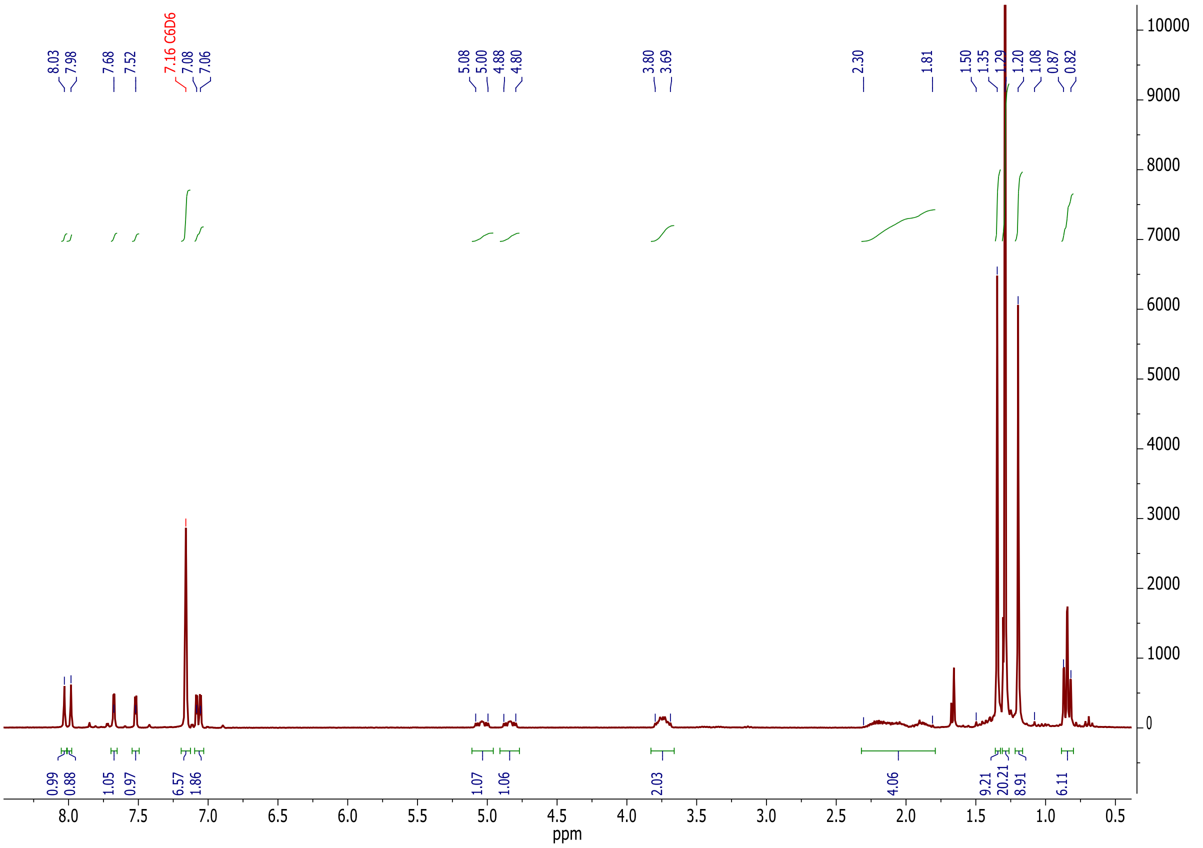

Figure S25. ^{1}H NMR spectrum of complex **9** in C_6D_6.

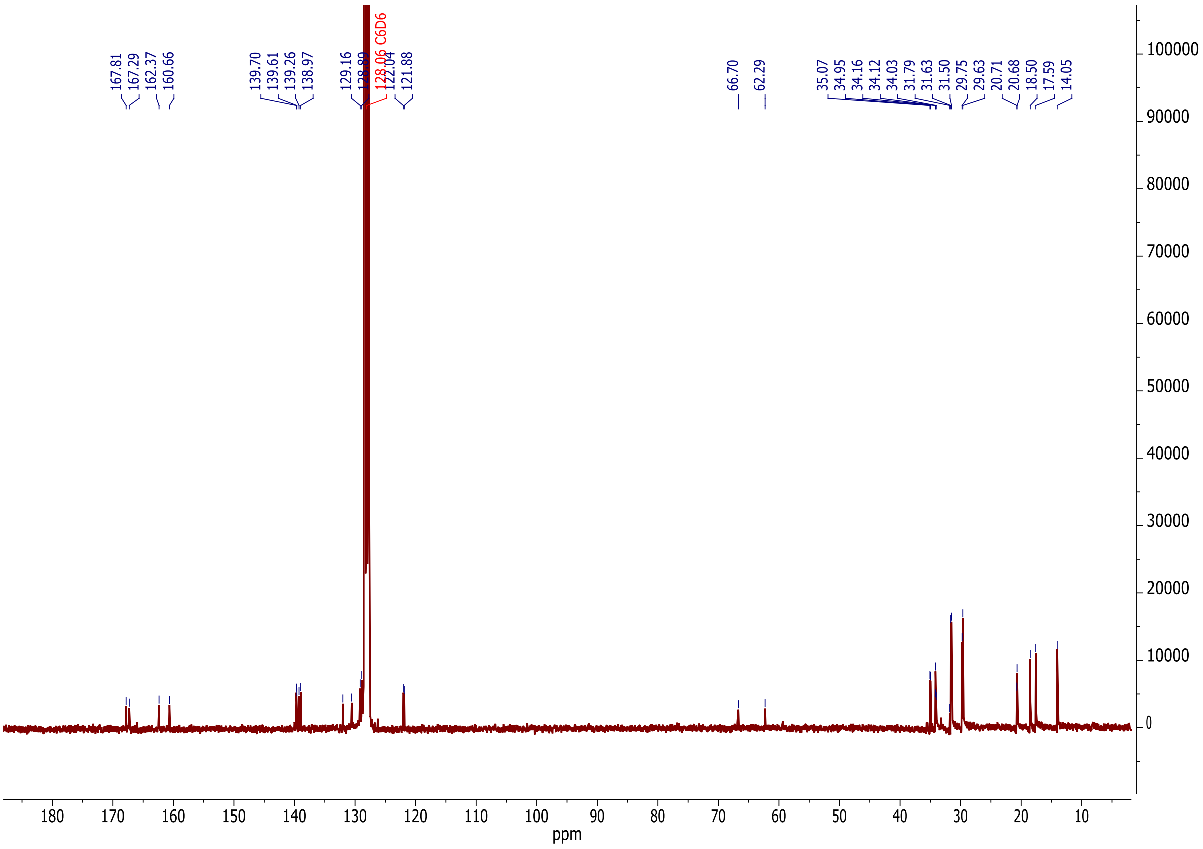

Figure S26. ^{13}C NMR spectrum of complex **9** in C_6D_6.

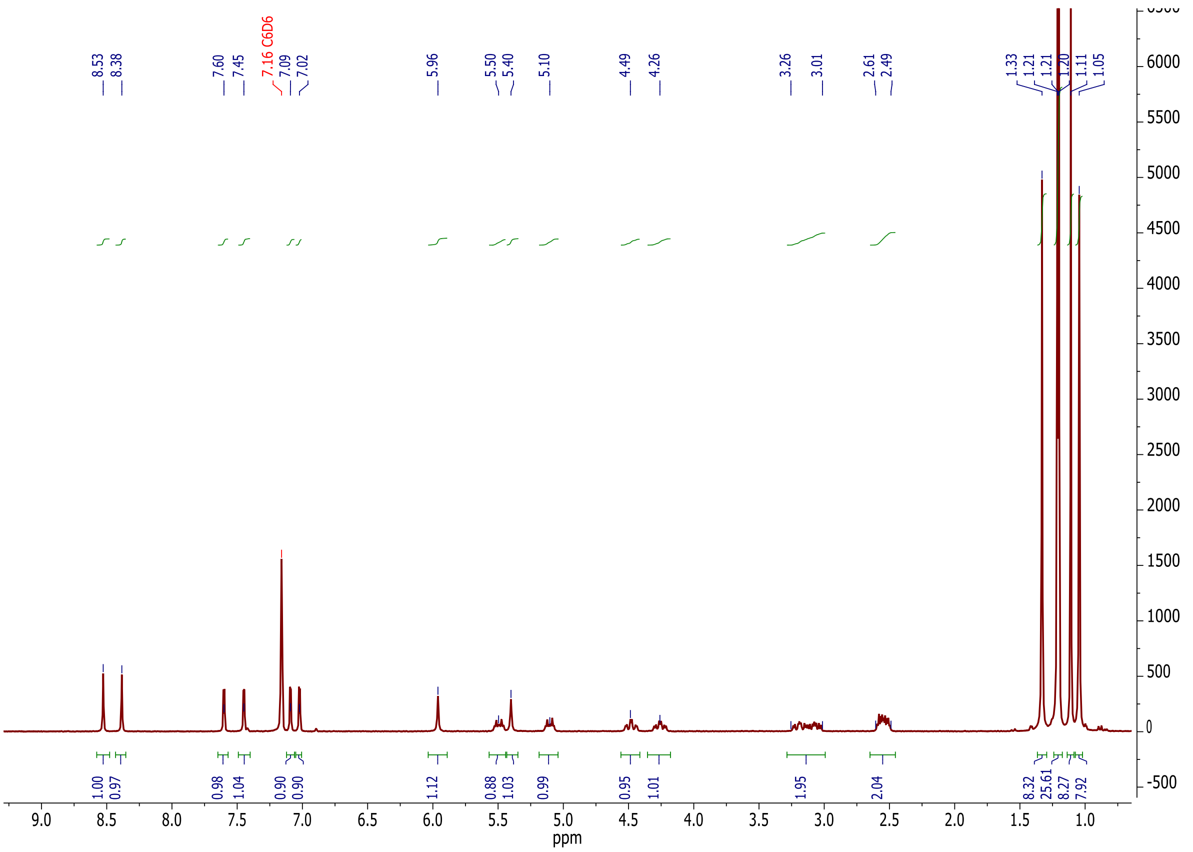

Figure S27. ^{1}H NMR spectrum of complex **10** in C_6D_6.

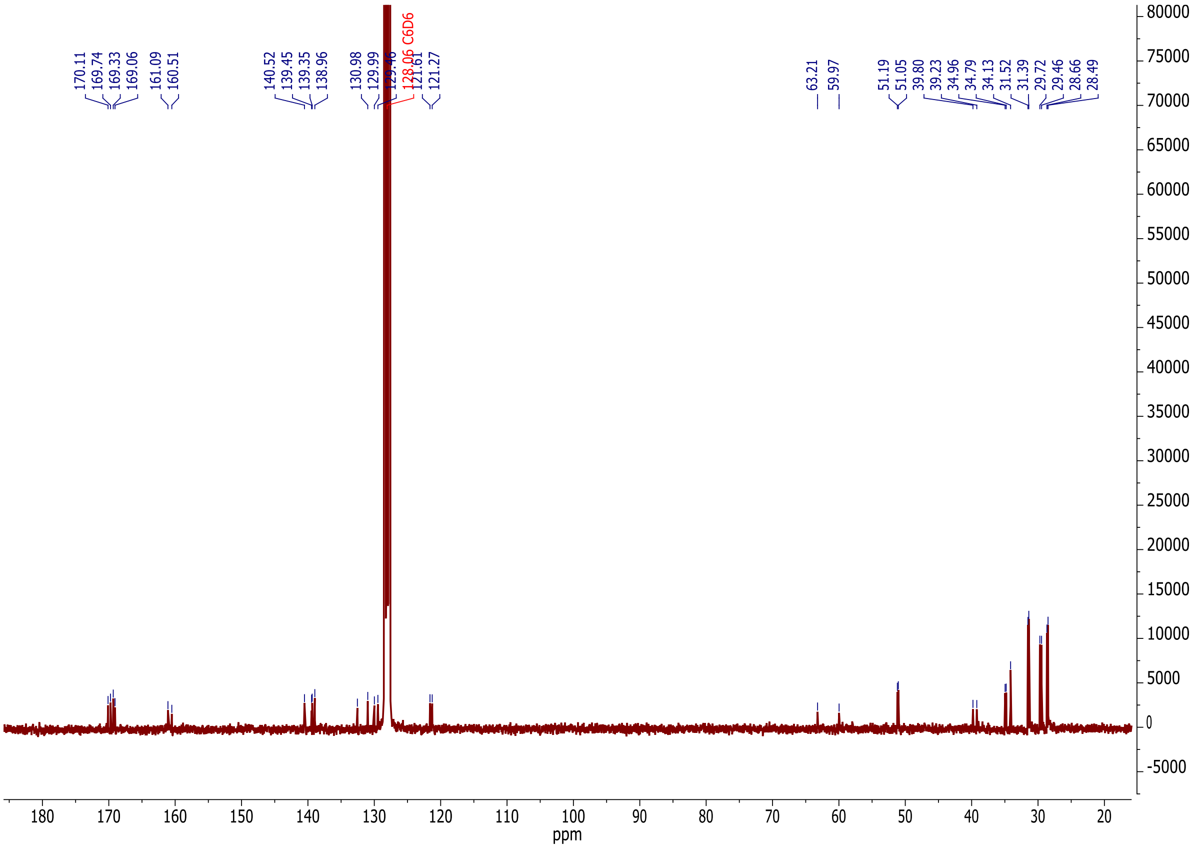

Figure S28. ^{13}C NMR spectrum of complex **10** in C_6D_6.

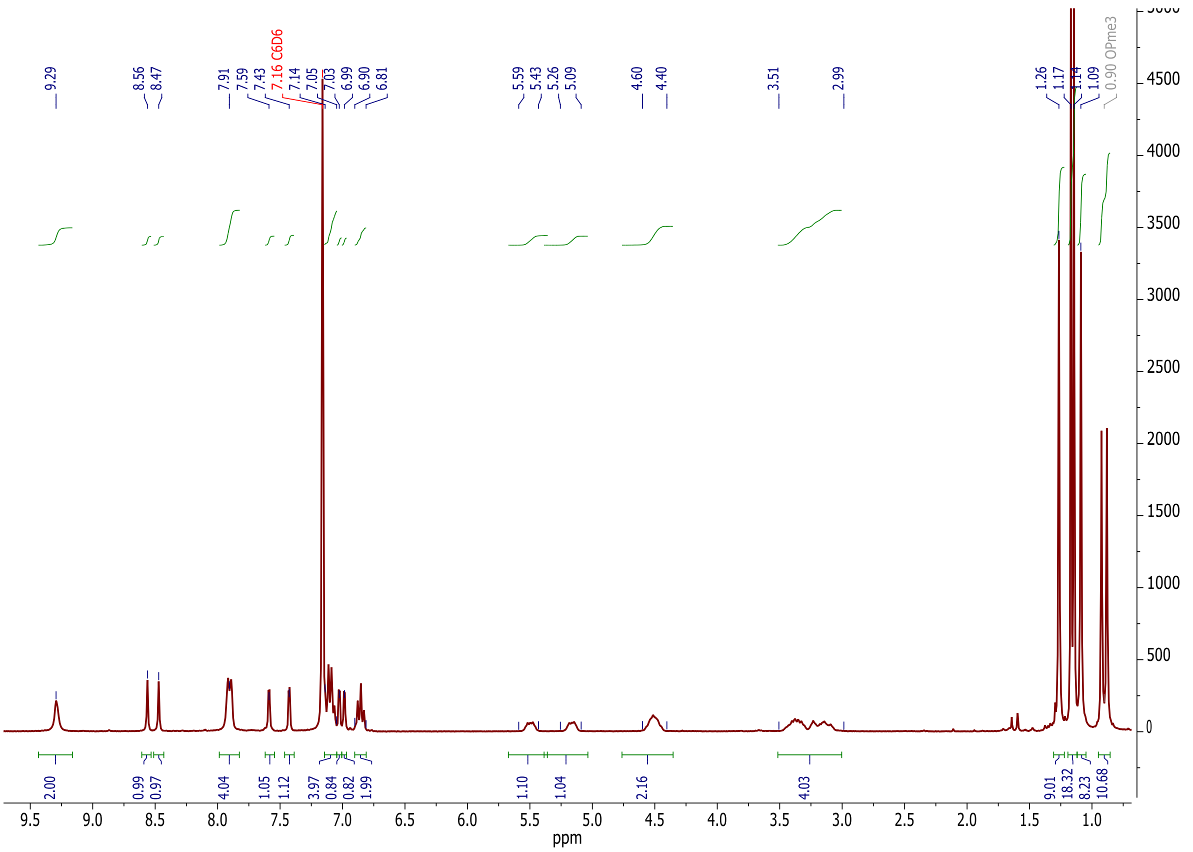

Figure S29. ^{1}H NMR spectrum of compound **11·OPMe**$_3$ in C_6D_6.

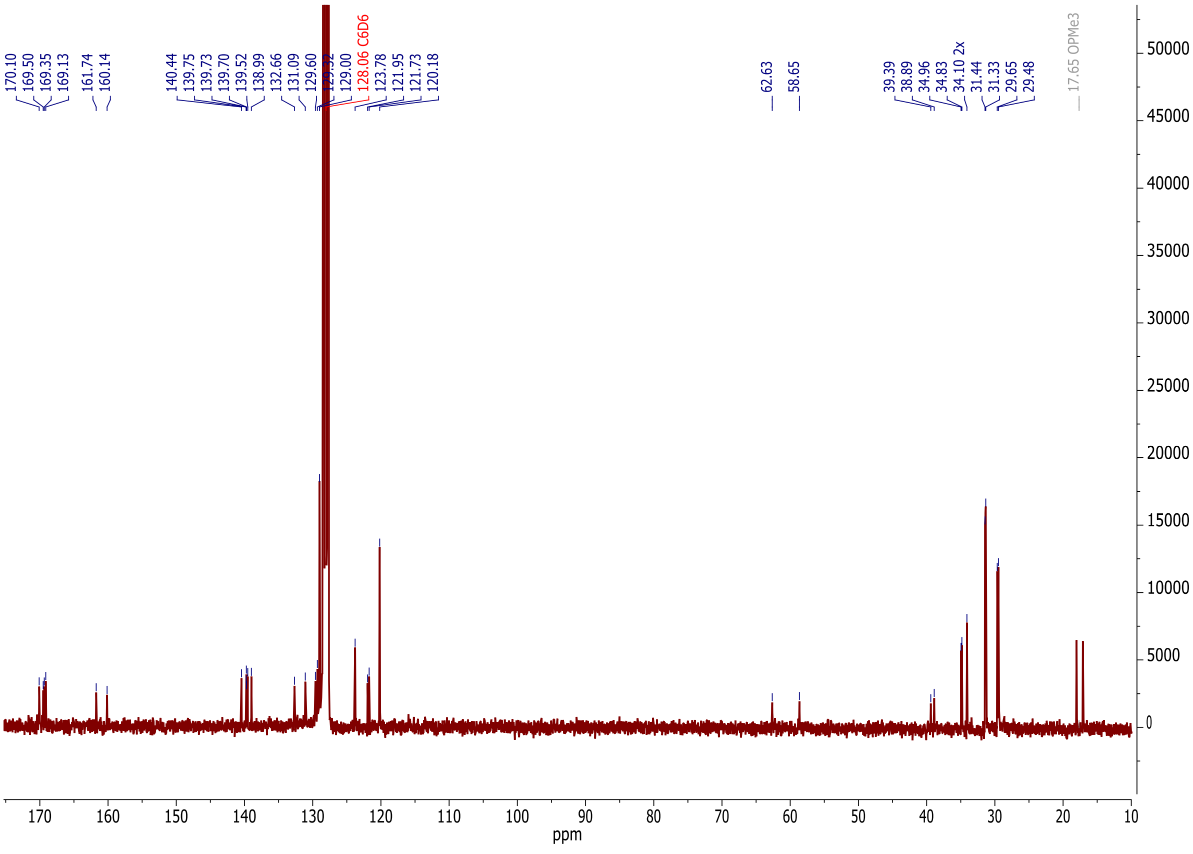

Figure S30. ^{13}C NMR spectrum of compound **11·OPMe**$_3$ in C_6D_6.

X-Ray single Crystal Structure Determination of HL3, 1-3, 3^{acac}, 7-10, 10·$OPMe_3$, 11·$OPMe_3$, 11·2$OPMe_3$ and 12

Table S1. Selected Crystallographic Data and Structure Refinement for **HL3**, **2**, **3**, **3^{acac}**, **7** and **8**.

	**HL3**	**1**	**2**	**3**	**3^{acac}**	**7**	**8**
Empirical formula	$C_{24}H_{32}N_2O_2$	$C_{38}H_{60}MoN_2O_4$ · $C_6H_{16}N^+Br^-$	$C_{44}H_{70}MoN_4O_6$ · $2C_2H_3N$	$C_{48}H_{62}MoN_4O_6$ · $4CH_3OH$	$C_{29}H_{38}MoN_2O_6$	$C_{88}H_{140}Mo_2N_8O_{11}$ · $3C_2H_3N$	$C_{88}H_{140}Mo_4N_8O_{18}$
Formula weight	380.51	886.92	929.08	1015.12	606.55	1801.11	1981.83
Crystal description	plate, yellow	plate, yellow	plate, yellow	needle,yellow	needle, yellow	needle, red	block, yellow
Crystal size	0.28 x 0.22 x 0.05 mm	0.28 x 0.17 x 0.07 mm	0.28 x 0.18 x 0.13mm	0.27 x 0.11 x 0.08mm	0.34 x 0.16 x 0.15mm	0.13 x 0.08 x 0.04mm	0.23 x 0.15 x 0.15mm
Crystal system, space group	triclinic, P -1	triclinic, P 1	Monoclinic, C 2/c	orthorhombic, P 2_1 2_1 2	monoclinic, P 2_1/c	monoclinic, P 2_1/n	tetragonal, I -4 2 d
Unit cell dimensions: a/Å	9.8997(8)	7.1260(5)	27.362(3)	15.454(3)	15.4131(18)	17.891(8)	16.1589(4)
b/Å	12.3846(10)	12.4430(9)	13.3862(16)	25.325(6)	7.5351(9)	18.434(9)	16.1589(4)
c/Å	19.4559(13)	13.6715(10)	16.5236(19)	6.8058(13)	25.632(3)	33.122(15)	38.8952(9)
α/°	102.933(3)	87.331(3)					
β/°	102.813(4)	75.155(2)	123.592(3)		102.859(5)	91.644(7)	
γ/°	102.822(4)	89.689(3)					
Volume/Å^3	2172.4(3)	1170.48(15)	5041.4(10)	2663.6(10)	2902.3(6)	10919(9)	10155.9(6)
Z	4	1	4	2	4	4	4
Unit cell determination	2.17° < Θ < 26.15°	2.96° < Θ < 42.64°	2.60° < Θ < 30.28°	2.64° < Θ < 25.68°	2.34° < Θ < 27.57°	2.20° < Θ < 24.78°	2.52° < Θ < 35.53
Reflections used	9347 (100K)	9360 (100K)	9930 (100K)	6729 (100K)	9947 (100K)	6196 (100K)	9772 (100K)
Θ range for data collection	1.76 to 26.00°	2.96 to 30.0°	2.47 to 30.00°	2.08 to 26.00°	2.71 to 27.00°	1.59 to 25.00°	2.01 to 35.00°
Reflections collected / unique	66434 / 8549	14083 / 9545	47034 / 7349	20961 / 5243	35374 / 6333	62343 / 19215	83813 / 11188
Significant unique refl.	5761 with I > 2 (I)	9155 with I > 2 (I)	6296 with I > 2 (I)	4712 with I > 2 (I)	5328 with I > 2 (I)	14132 with I > 2 (I)	10227 with I > 2 (I)
R(int), R(sigma)	0.0667, 0.0730	0.0326, 0.0713	0.0582, 0.0423	0.0529, 0.0486	0.0323, 0.0288	0.0716, 0.0989	0.0353, 0.0234
Completeness to Θ	100.0% (26.00°)	99.9 % (30.0°)	99.9%	99.8% (26.0°)	99.9% (27.0°)	99.9% (25.0°)	99.9% (35.0°)
Data/parameters/restraints	8549 / 553 / 4	9545 / 512 / 4	7349 / 303 / 1	5243 / 351 / 10	6333 / 368 / 1	19215 / 1134 / 35	11188 / 292 / 1
Goodness-of-fit on F^2	1.011	1.034	1.092	1.047	1.076	1.042	1.019
Final R indices [I > 2 (I)]	R1 = 0.0438, wR2 = 0.0962	R1 = 0.0373, wR2 = 0.0970	R1 = 0.0302, wR2 = 0.0732	R1 = 0.0381, wR2 = 0.0902	R1 = 0.0314, wR2 = 0.0739	R1 = 0.0609, wR2 = 0.1404	R1 = 0.0227, wR2 = 0.0532
R indices (all data)	R1 = 0.0798, wR2 = 0.1089	R1 = 0.0385, wR2 = 0.0973	R1 = 0.0410, wR2 = 0.0779	R1 = 0.0461, wR2 = 0.0937	R1 = 0.0426, wR2 = 0.0803	R1 = 0.0865, wR2 = 0.1479	R1 = 0.0273, wR2 = 0.0549
Weighting scheme param. a, b	0.0429, 0.0559	0.0393, 0.4566	0.0279, 3.9216	0.0548, 0.1213	0.0359, 2.6324	0.0739, 0.0000	0.0279, 3.2408
Larg. / in last cycle	0.001	0.001	0.001	0.006	0.003	0.002	0.001
Largest diff. peak and hole	0.274 and -0.263e/Å^3	2.453 and -1.381e/Å^3	0.531 and -0.431e/Å^3	0.557 and -0.454e/Å^3	0.884 and -0.578e/Å^3	0.949 and -0.954e/Å^3	0.355 and -0.329e/Å^3
CCDC	1865601	1865602	1865603	1865604	1865605	1865606	1865607
Weighting scheme	$w=1/[\ {}^2(F_o^2)+(aP)^2+bP]$ where $P=(F_o^2+2F_c^2)/3$						

Table S2. Selected Crystallographic Data and Structure Refinement for **9**, **10**, **10·OPMe$_3$**, **11·OPMe$_3$**, **11·2OPMe$_3$** and **12**.

	**9**	**10**	**10·OPMe$_3$**	**11·OPMe$_3$**	**11·2OPMe$_3$**	**12**
Empirical formula	$C_{38}H_{60}MoN_2O_5$	$C_{44}H_{70}MoN_4O_7$	$C_{44}H_{70}MoN_4O_7 \cdot C_3H_9OP \cdot 1.5C_6H_6$	$C_{48}H_{62}MoN_4O_7 \cdot C_3H_9OP \cdot 2C_2H_3N$	$C_{48}H_{62}MoN_4O_7 \cdot 2C_3H_9OP \cdot C_2H_3N$	$[C_9H_{13}N_2O]^{+}{}_4[Mo_8O_{26}]^{4-} \cdot 6C_3H_9OP$
Formula weight	720.82	862.98	1072.21	1077.13	1128.15	2396.80
Crystal description	block, orange	plate, orange	needle, orange	block, orange	needle, yellow	plate, orange
Crystal size	0.21 x 0.14 x 0.11 mm	0.23 x 0.23 x 0.10 mm	0.31 x 0.12 x 0.07 mm	0.25 x 0.12 x 0.11 mm	0.34 x 0.04 x 0.03 mm	0.31 x 0.17 x 0.06 mm
Crystal system, space group	monoclinic, P 2_1/c	triclinic, P -1	triclinic, P -1	monoclinic, P 2_1/c	monoclinic, P 2_1/n	triclinic, P -1
Unit cell dimensions: a/Å	14.5155(6)	11.0973(8)	10.493(4)	14.8508(6)	14.6489(11)	10.2915(8)
b/Å	13.1655(5)	11.1593(8)	14.499(5)	26.5216(12)	28.184(2)	11.4475(9)
c/Å	20.5026(8)	37.919(3)	20.935(7)	16.5935(9)	29.156(3)	19.6869(15)
α/°		87.6027(15)	72.273(4)			77.147(2)
β/°	107.9113(17)	82.6283(16)	84.968(4)	114.488(2)	92.308(4)	86.207(3)
γ/°		86.7040(15)	80.738(3)			75.849(2)
Volume/Å^3	3728.2(3)	4646.5(6)	2991.6(18)	5947.7(5)	12027.7(18)	2192.5(3)
Z	4	4	2	4	8	1
Unit cell determination	2.57° < Θ < 30.57°	2.31° < Θ < 26.60°	2.18° < Θ < 26.38°	2.52° < Θ < 24.64°	2.41° < Θ < 25.01°	2.29° < Θ < 26.68°
Reflections used	8735 (100K)	9424 (100K)	8542 (100K)	5495 (100K)	6780 (100K)	9861 (100K)
Θ range for data collection	2.57 to 30.00°	2.53 to 26.00°	1.49 to 26.00°	2.15 to 25.00°	2.01 to 25.00°	2.12 to 26.00°
Reflections collected / unique	19708 / 10812	61246 / 18242	34990 / 11732	38565 / 10487	102526 / 21180	39459 / 8605
Significant unique refl.	8844 with I > 2 (I)	16161 with I > 2 (I)	9107 with I > 2 (I)	6788 with I > 2 (I)	14806 with I > 2 (I)	6343 with I > 2 (I)
R(int), R(sigma)	0.0233, 0.0380	0.0289, 0.0293	0.0728, 0.0901	0.0826, 0.1113	0.0731, 0.0936	0.0589, 0.0716
Completeness to Θ	99.4% (30.0°)	99.9 % (26.0°)	99.9%	99.9% (25.0°)	99.9% (25.0°)	99.8% (26.0°)
Data/parameters/restraints	10812 / 481 / 0	18242 / 1167 / 11	11732 / 672 / 2	10487 / 668 / 12	21180 / 1392 / 4	8605 / 549 / 8
Goodness-of-fit on F^2	1.021	1.078	1.073	1.007	1.040	1.037
Final R indices [I > 2 (I)]	R1 = 0.0305, wR2 = 0.0709	R1 = 0.0337, wR2 = 0.0753	R1 = 0.0602, wR2 = 0.1543	R1 = 0.0623, wR2 = 0.1413	R1 = 0.0698, wR2 = 0.1649	R1 = 0.0393, wR2 = 0.0869
R indices (all data)	R1 = 0.0439, wR2 = 0.0760	R1 = 0.0403, wR2 = 0.0781	R1 = 0.0805, wR2 = 0.1643	R1 = 0.1114, wR2 = 0.1599	R1 = 0.1009, wR2 = 0.1743	R1 = 0.0600, wR2 = 0.0939
Weighting scheme param. a, b	0.0325, 1.7267	0.0234, 4.2346	0.0808, 2.5227	0.0760, 3.3558	0.0943, 0.0000	0.0108, 4.1376
Larg. / in last cycle	0.002	0.002	0.002	0.001	0.003	0.002
Largest diff. peak and hole	0.574 and -0.596e/Å^3	0.616 and -0.614e/Å^3	1.338 and -1.265e /Å^3	0.893 and -0.947e/Å^3	1.330 and -1.301e/Å^3	1.175 and -0.853e /Å^3
CCDC	1865608	1865609	1865610	1865611	1865612	1865613
Weighting scheme	w=1/[$^2(F_o^2)+(aP)^2+bP$] where $P=(F_o^2+2F_c^2)/3$					

Crystal Structure Determinations – General

All the measurements were performed using monochromatized Mo K radiation at 100K. Crystallographic data, refinement parameters and results are given in Tables S1 and S2 and in the CIF. The structures were solved by direct methods (SHELXS-97)[4] and refined by full-matrix least-squares techniques against F^2 (SHELXL-2014/6)[5]. The non-hydrogen atoms were refined with anisotropic displacement parameters without any constraints. The absolute configurations of compounds **1** and **8** were established by anomalous dispersion effects in the diffraction measurements on the crystals. The positions of the H atoms of the NH groups and of the OH groups were taken from a difference Fourier map, the N–H distances were fixed to 0.88 Å, the O–H distances were fixed to 0.84 Å, and the H atoms were refined with individual isotropic displacement parameters without any constraints to the bond angles. The H atoms of the phenyl rings including any adjacent CH=N group were put at the external bisectors of the C–C–C angles at C–H distances of 0.95 Å and common isotropic displacement parameters were refined for the H atoms of the same ring. The H atoms of the CH_2 groups were refined with common isotropic displacement parameters for the H atoms of the same group and idealized geometry with approximately tetrahedral angles and C–H distances of 0.99 Å. The H atoms of the methyl groups were refined with common isotropic displacement parameters for the H atoms of the same group and idealized geometries with tetrahedral angles, enabling rotation around the C–C bonds, and C–H distances of 0.98 Å.

Crystal Structure Determination – Additional Refinement Informations

Compound 3: Since racemic twinning was detected a twin matrix (-1 0 0 / 0 -1 0 / 0 0 -1) was applied. Because the refined factor between the two unequal components was not significantly different from 0.5 [e.g. 0.47(5)] it was fixed at 0.5: the crystal was refined as a perfect inversion twin. The *tert*-butyl group bonded to C14 was disordered over two orientations and refined to site occupation factors of 0.645(7) and 0.355(7), respectively. In this disordered group the C–C bonds were restrained to 1.53 Å and the same anisotropic displacement parameters were used for equivalent C atoms. The other non-hydrogen atoms of the metal complex were refined with anisotropic displacement parameters without any constraints. One methanol solvent molecule was disordered, too. The two orientations were refined with site occupation factors of 0.5 as rigid bodies.

Compound 3^{acac}: The H atoms of the methyl group C25 are disordered over two orientations and were refined with site occupation factors of 0.5 at two positions rotated from each other by 60° with a common isotropic displacement parameter and idealized geometry with tetrahedral angles, enabling rotation around the C–C bond, and C–H distances of 0.98 Å. The H atoms of the other methyl groups

were refined with common isotropic displacement parameters for the H atoms of the same group and the same idealized geometries.

Compound 7: The *tert*-butyl groups bonded to C34 and to C44 were disordered over two orientations and refined to site occupation factors of 0.617(6) and of 0.615(5), resp., for the most occupied orientations. In these disordered groups the C–C bonds were restrained to 1.53 Å and the same anisotropic displacement parameters were used for equivalent C atoms. The other non-hydrogen atoms of the metal complex were refined with anisotropic displacement parameters without any constraints. The three acetonitrile solvent molecules are disordered over two or three orientations. Their bond lengths were restrained and the same anisotropic displacement parameters were used for the atoms in the same void. The H atoms of the acetonitrile solvent molecules were included with their isotropic displacement parameters fixed to 1.2 times U_{eq} of the C atom they are bonded to and with idealized geometries with tetrahedral angles, enabling rotation around the C–C bonds, and C–H distances of 0.98 Å.

Compound 9: The oxo and peroxo groups bonded to Mo are disordered over two orientations and were refined with site occupation factors of 0.771(5) and 0.229(5), respectively. The non-hydrogen atoms were refined with anisotropic displacement parameters without any constraints. The H atoms H17 and H27 were put at the external bisectors of the C–C=N angles at C–H distances of 0.95 Å but the individual isotropic displacement parameters were free to refine.

Compound 10: The asymmetric unit consists of two molecules A and B. The *tert*-butyl group bonded to C14 was disordered over two orientations and was refined with site occupation factors of 0.672(5) and 0.328(5), respectively. The non-hydrogen atoms of molecule A were refined with anisotropic displacement parameters without any constraints. A part of molecule B was disordered over two orientations and was refined with site occupation factors of 0.537(4) and 0.463(4), respectively. In this disordered fragment the same anisotropic displacement parameters were used for atoms whose positions are close together and the equivalent bonds in the disordered (3-amino-3-oxopropyl)imino group were restrained to have the same lengths. The other non-hydrogen atoms of molecule B were refined with anisotropic displacement parameters without any constraints.

Compound 11·OPMe$_3$: One of the two acetonitrile solvate molecules was disordered over four orientations. The same anisotropic displacement parameters were used for the atoms of the disordered molecules in about the same orientations, the C–C bonds were restrained to 1.455(3) Å, the C-N bonds were restrained to 1.138(3) Å, and their site occupation factors were fixed to 0.25. The other non-hydrogen atoms were refined with anisotropic displacement parameters without any constraints.

Compound 12: The positions of the H atoms of the NH_3 groups were taken from a difference Fourier map, the N–H distances were fixed to 0.91 Å, and the H atoms were refined with common isotropic displacement parameters without any constraints to the bond angles.

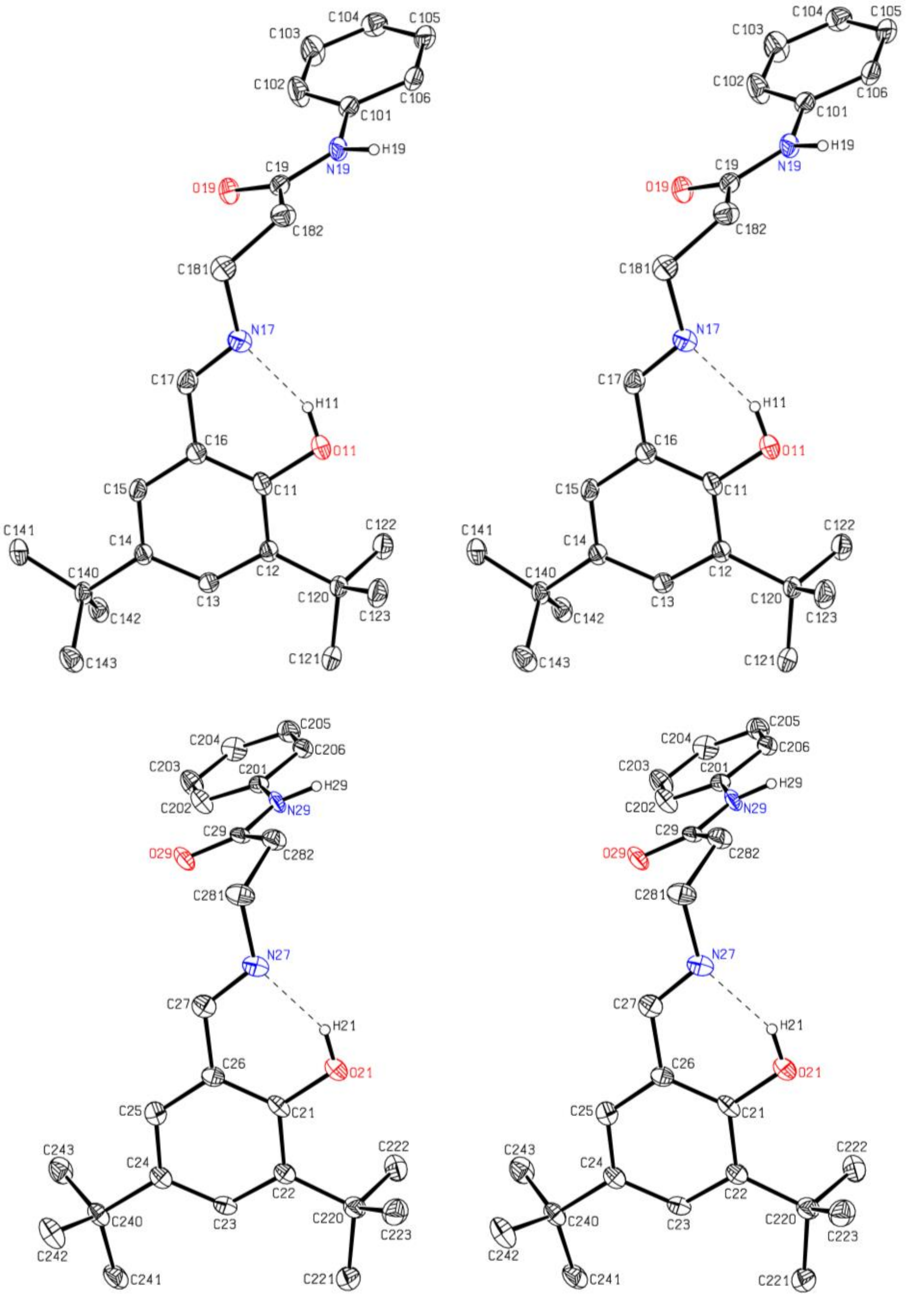

Figure S31. Stereoscopic ORTEP[6] plot of molecule A (above) and of molecule B (below) of **HL3** showing the atomic numbering scheme. The probability ellipsoids are drawn at the 50% probability level. The H atoms bonded to N or O are drawn with arbitrary radii, the other H atoms were omitted for clarity. The intra-molecular hydrogen bond is indicated by a dashed line.

Table S3. Hydrogen bonds [Å, °] for ligand **HL3**.

D-H···A	d(D-H)	d(H···A)	d(D···A)	<(DHA)
O(11)-H(11)···N(17)	0.84	1.824(8)	2.6019(18)	153.3(16)
N(19)-H(19)···O(29)	0.88	2.0435(17)	2.9206(17)	174.4(8)
O(21)-H(21)···N(27)	0.84	1.837(5)	2.5950(18)	149.3(10)
N(29)-H(29)···O(19)[i)]	0.88	2.1721(17)	3.0487(16)	174.0(5)

Symmetry transformation used to generate equivalent atoms: [i)] x-1, y, z

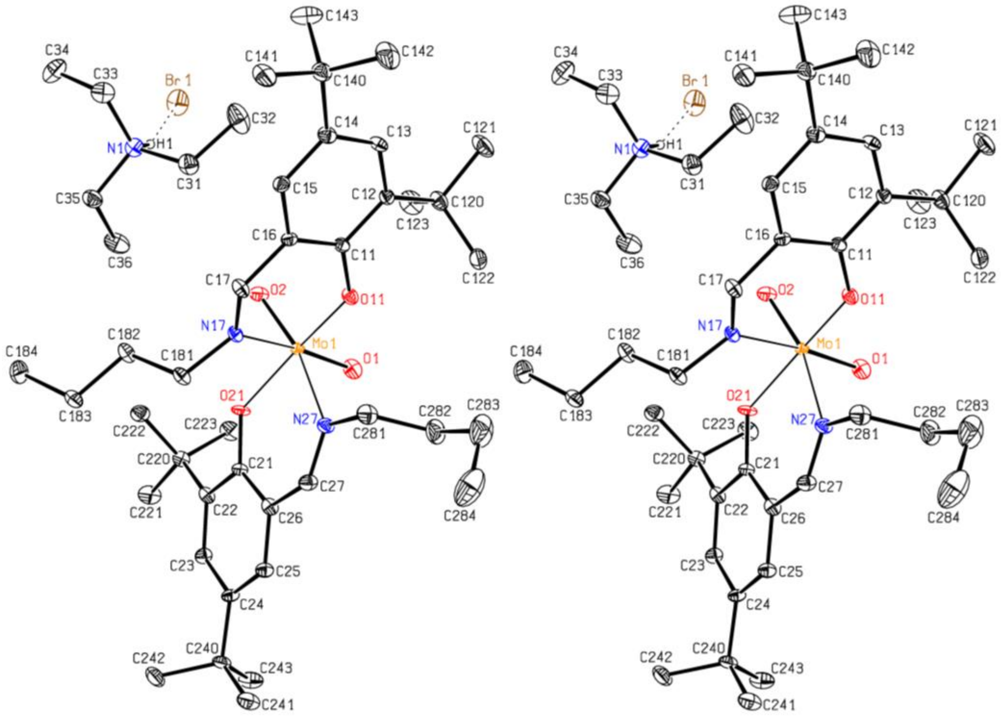

Figure S32. Stereoscopic ORTEP[6] plot of the asymmetric unit of **1** showing the atomic numbering scheme. The probability ellipsoids are drawn at the 50% probability level. The H atom H1 is drawn with an arbitrary radius, the other H atoms were omitted for clarity reasons. The hydrogen bond is indicated by a dotted line.

Table S4. Selected bond lengths [Å] and angles [°] for **1**.

Mo(1)-O(1)	1.705(3)	O(11)-C(11)	1.341(5)
Mo(1)-O(2)	1.712(3)	C(17)-N(17)	1.283(5)
Mo(1)-O(11)	1.952(3)	N(17)-C(181)	1.479(5)
Mo(1)-O(21)	1.955(3)	O(21)-C(21)	1.337(5)
Mo(1)-N(17)	2.351(3)	C(27)-N(27)	1.298(5)
Mo(1)-N(27)	2.341(4)	N(27)-C(281)	1.480(5)
O(1)-Mo(1)-O(2)	106.80(16)	O(1)-Mo(1)-N(17)	163.26(14)
O(11)-Mo(1)-O(21)	160.48(11)	O(2)-Mo(1)-N(27)	161.75(14)

N(17)-Mo(1)-N(27)	73.30(12)	C(21)-O(21)-Mo(1)	137.1(3)
C(11)-O(11)-Mo(1)	135.0(3)	C(27)-N(27)-C(281)	113.1(4)
C(17)-N(17)-C(181)	114.9(3)	C(27)-N(27)-Mo(1)	124.5(3)
C(17)-N(17)-Mo(1)	125.1(3)	C(281)-N(27)-Mo(1)	122.4(3)
C(181)-N(17)-Mo(1)	120.0(2)		

Table S5. Hydrogen bond [Å, °] for **1**.

D-H···A	d(D-H)	d(H···A)	d(D···A)	<(DHA)
N(1)-H(1)···Br(1)	0.88	2.378(7)	3.216(3)	159.2(14)

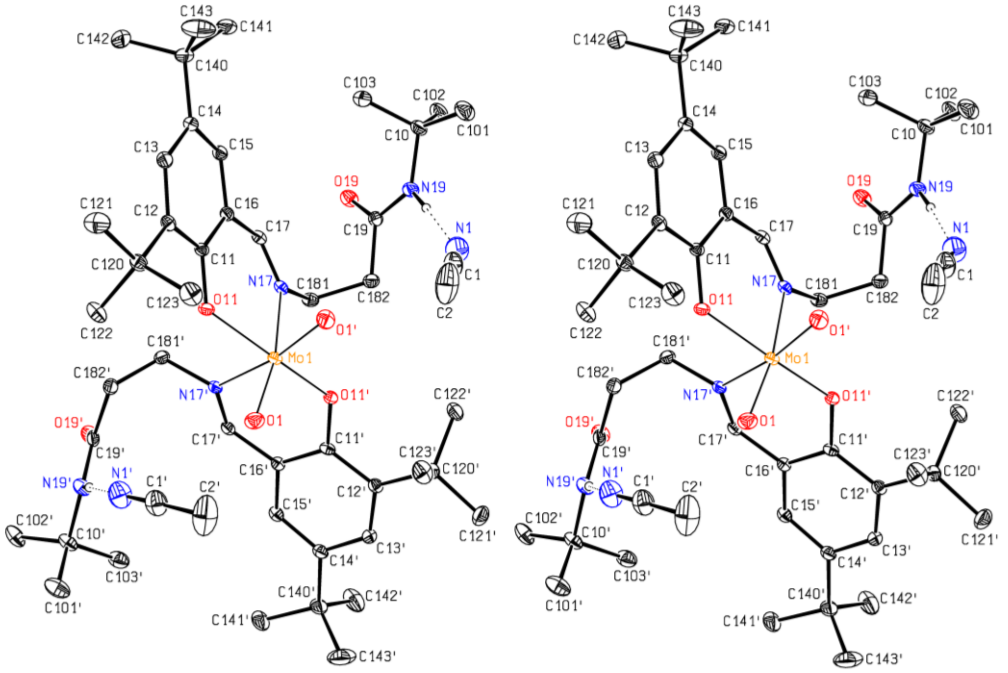

Figure S33. Stereoscopic ORTEP[6] plot of **2** showing the atomic numbering scheme. The probability ellipsoids are drawn at the 50% probability level. The H atoms bonded to N are drawn with arbitrary radii, the other H atoms were omitted for clarity. The hydrogen bonds are indicated by dotted lines.

Table S6. Selected bond lengths [Å] and angles [°] for **2.**

Mo(1)-O(1)	1.7078(10)	N(17)-C(181)	1.4798(17)
Mo(1)-O(11)	1.9571(10)	C(19)-O(19)	1.2295(17)
Mo(1)-N(17)	2.3548(12)	C(19)-N(19)	1.3462(19)
O(11)-C(11)	1.3485(15)	N(19)-C(10)	1.4815(18)
C(17)-N(17)	1.2882(17)		
O(1)-Mo(1)-O(1)$^{i)}$	107.62(7)	O(11)-Mo(1)-O(11)$^{i)}$	157.63(6)

O(1)-Mo(1)-N(17)	162.85(5)	C(17)-N(17)-C(181)	116.24(11)
N(17)-Mo(1)-N(17)[i)]	73.42(6)	C(17)-N(17)-Mo(1)	122.79(9)
C(11)-O(11)-Mo(1)	130.14(9)	C(181)-N(17)-Mo(1)	120.96(8)
N(17)-C(17)-C(16)	125.60(12)		

Symmetry transformations used to generate equivalent atoms: [i)] 1-x, y, 3/2-z

Table S7. Hydrogen bond [Å, °] for **2**.

D-H···A	d(D-H)	d(H···A)	d(D···A)	<(DHA)
N(19)-H(19)···N(1)	0.88	2.196(3)	3.0701(19)	172.0(14)

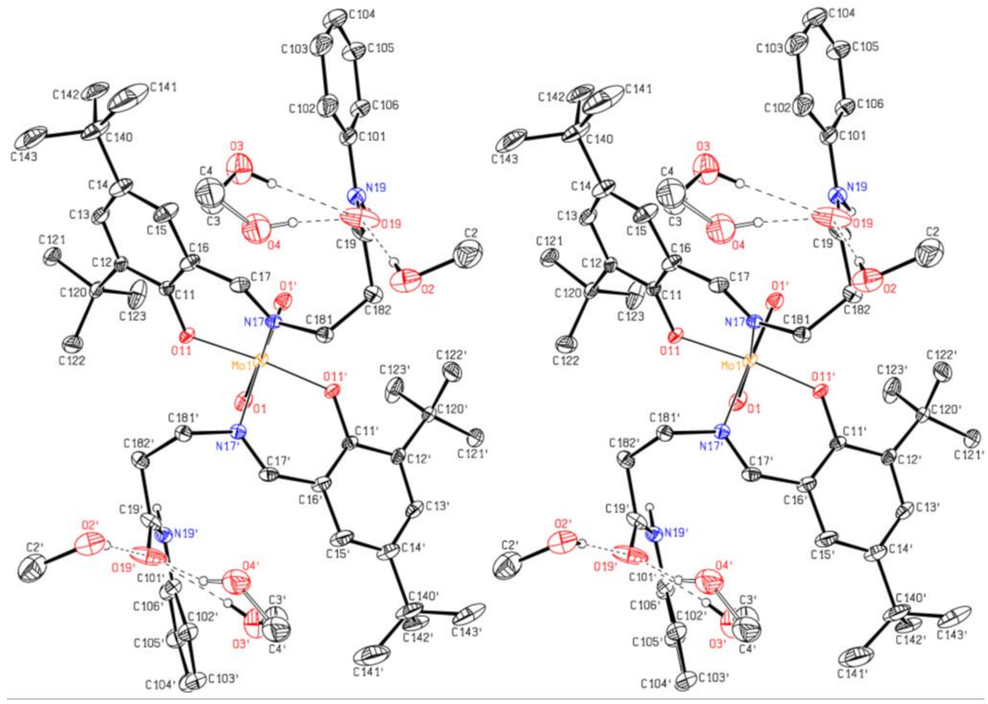

Figure S34. Stereoscopic ORTEP[6] plot of **3** showing the atomic numbering scheme. The probability ellipsoids are drawn at the 30% probability level. The H atoms bonded to N or O are drawn with arbitrary radii, the other H atoms as well as the disordered *tert*-butyl group with a site occupation factor of 0.355(7) were omitted for clarity. Two of the disordered methanol molecules are plotted with open bonds, the hydrogen bonds are indicated by dotted lines.

Table S8. Selected bond lengths [Å] and angles [°] for **3**.

Mo(1)-O(1)	1.708(3)	O(11)-C(11)	1.350(5)
Mo(1)-O(11)	1.950(2)	C(17)-N(17)	1.275(5)
Mo(1)-N(17)	2.333(3)	N(17)-C(181)	1.469(5)
O(1)-Mo(1)-N(17)	163.23(14)	C(11)-O(11)-Mo(1)	131.8(2)
O(11)-Mo(1)-O(11)[i)]	159.49(16)	C(17)-N(17)-C(181)	114.6(4)
O(1)-Mo(1)-O(1)[i)]	108.0(2)	C(17)-N(17)-Mo(1)	123.8(3)
N(17)-Mo(1)-N(17)[i)]	74.78(16)	C(181)-N(17)-Mo(1)	121.6(2)

Symmetry transformation used to generate equivalent atoms: [i)] 1-x, 1-y, z

Table S9. Hydrogen bonds [Å, °] for **3**.

D-H···A	d(D-H)	d(H···A)	d(D···A)	<(DHA)
N(19)-H(19)···O(2)[i)]	0.88	1.9774(3)	2.8398(4)	166.20(2)
O(2)-H(2)···O(19)	0.84	1.847(4)	2.679(4)	170.63(15)
O(3)-H(3)···O(19)	0.84	2.261(6)	3.099(6)	174.95(11)
O(4)-H(4)···O(19)	0.84	2.030(5)	2.817(5)	155.84(11)

Symmetry transformation used to generate equivalent atoms: [i)] x, y, z-1

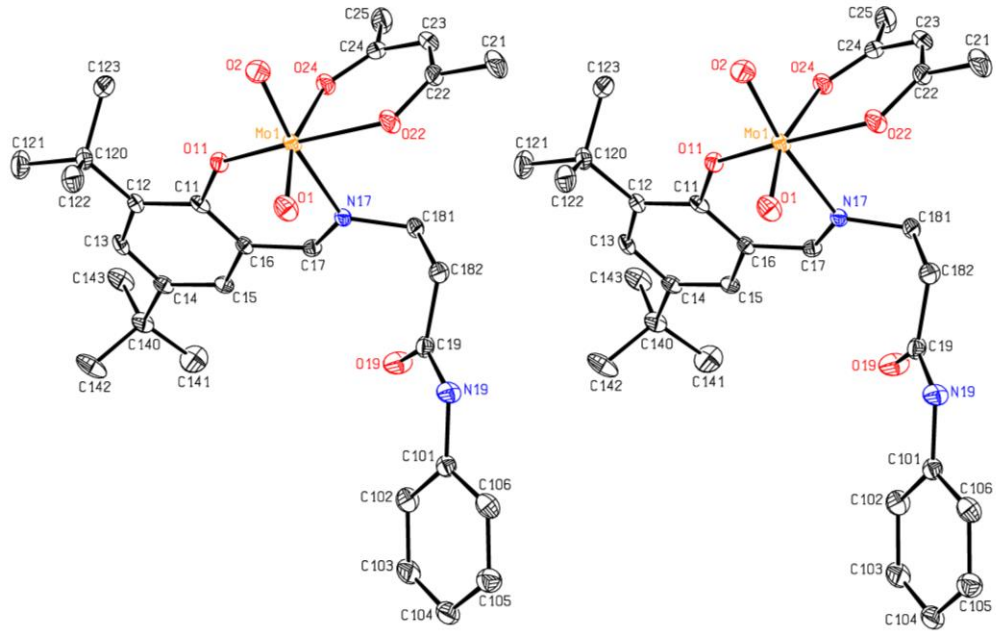

Figure S35. Stereoscopic ORTEP[6] plot of **3**[acac] showing the atomic numbering scheme. The probability ellipsoids are drawn at the 50% probability level. The H atoms were omitted for clarity.

Table S10. Selected bond lengths [Å] and angles [°] for **3**[acac].

Mo(1)-O(1)	1.6969(16)	O(11)-C(11)	1.344(2)
Mo(1)-O(2)	1.7119(15)	C(17)-N(17)	1.287(3)
Mo(1)-O(11)	1.9069(14)	N(17)-C(181)	1.479(3)
Mo(1)-O(22)	2.0288(15)	C(22)-O(22)	1.311(3)
Mo(1)-O(24)	2.2012(15)	C(24)-O(24)	1.266(3)
Mo(1)-N(17)	2.3393(18)		
O(11)-Mo(1)-O(22)	160.61(6)	C(17)-N(17)-Mo(1)	124.32(14)
O(1)-Mo(1)-O(24)	161.28(7)	C(181)-N(17)-Mo(1)	119.94(12)
O(2)-Mo(1)-N(17)	166.13(7)	C(22)-O(22)-Mo(1)	133.33(14)
C(11)-O(11)-Mo(1)	142.48(14)	C(24)-O(24)-Mo(1)	131.50(14)
C(17)-N(17)-C(181)	115.71(18)		

Table S11. Hydrogen bond [Å, °] for **3**[acac].

D-H···A	d(D-H)	d(H···A)	d(D···A)	<(DHA)
N(19)-H(19)···O(2)[i]	0.88	2.408(5)	3.250(2)	160.3(11)

Symmetry transformation used to generate equivalent atoms: [i] 2-x, 1-y, 1-z

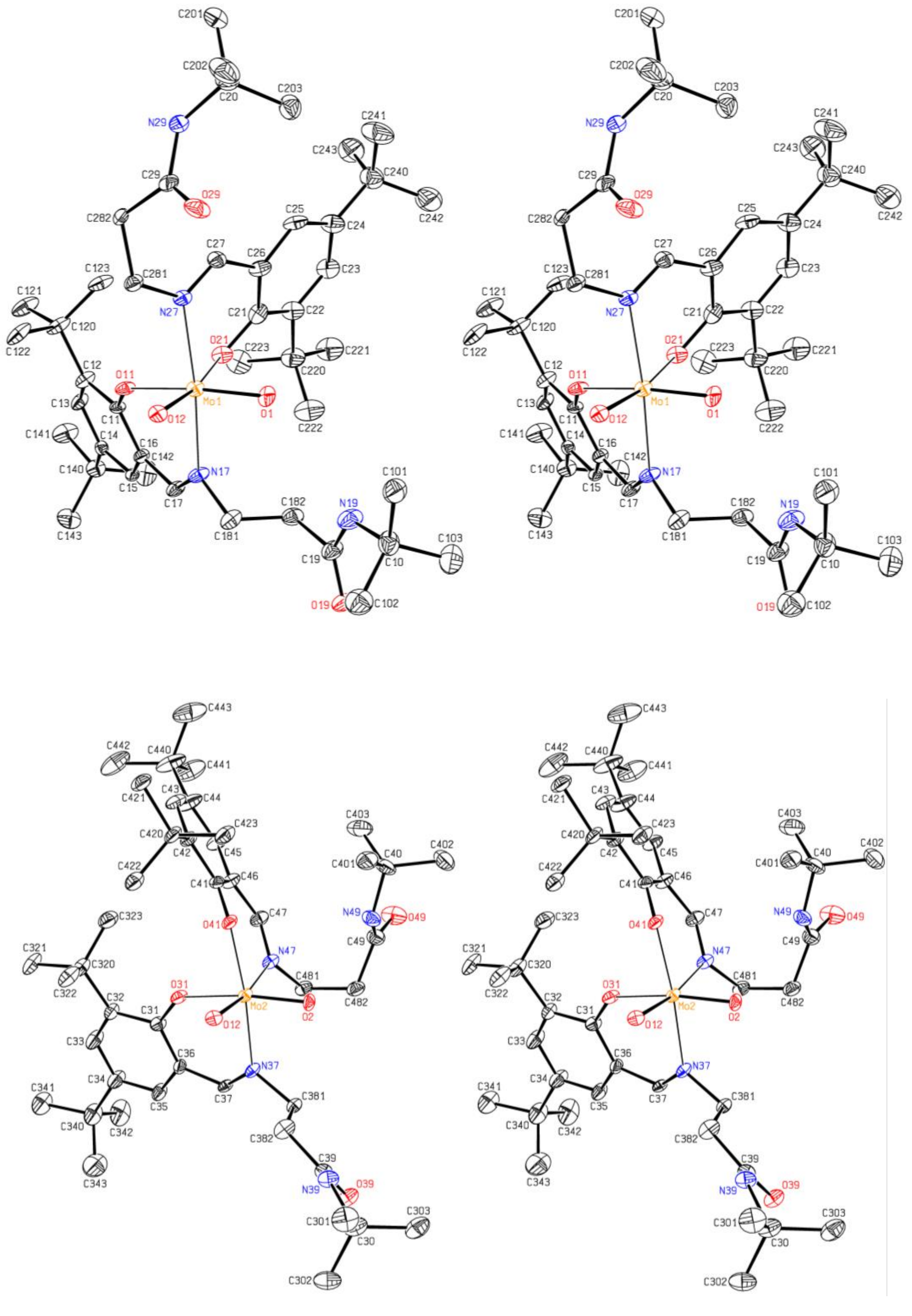

Figure S36. Stereoscopic ORTEP[6] plot of the ligands bonded to Mo1 (above) and those bonded to Mo2 (below) of **7** showing the atomic numbering scheme. The probability ellipsoids are drawn at the 30% probability level. The H atoms were omitted for clarity.

Table S12. Selected bond lengths [Å] and angles [°] for **7**.

O(12)-Mo(1)	1.878(2)	Mo(1)-N(27)	2.165(3)
O(12)-Mo(2)	1.883(2)	Mo(2)-O(2)	1.709(2)
Mo(1)-O(1)	1.701(3)	Mo(2)-O(31)	2.012(3)
Mo(1)-O(11)	1.995(2)	Mo(2)-O(41)	1.999(2)
Mo(1)-O(21)	2.063(2)	Mo(2)-N(37)	2.207(3)
Mo(1)-N(17)	2.185(3)	Mo(2)-N(47)	2.231(3)
Mo(1)-O(12)-Mo(2)	163.04(14)	C(21)-O(21)-Mo(1)	123.3(2)
O(1)-Mo(1)-O(11)	160.39(10)	C(27)-N(27)-C(281)	122.4(3)
O(12)-Mo(1)-O(11)	95.27(10)	C(27)-N(27)-Mo(1)	120.7(3)
O(12)-Mo(1)-O(21)	170.53(10)	C(281)-N(27)-Mo(1)	116.6(2)
O(11)-Mo(1)-O(21)	78.02(10)	O(2)-Mo(2)-O(31)	164.10(11)
O(12)-Mo(1)-N(17)	92.41(10)	O(12)-Mo(2)-O(31)	92.14(10)
O(12)-Mo(1)-N(27)	90.60(10)	O(12)-Mo(2)-O(41)	88.28(10)
N(17)-Mo(1)-N(27)	171.62(10)	O(31)-Mo(2)-O(41)	88.27(10)
C(11)-O(11)-Mo(1)	130.7(2)	O(12)-Mo(2)-N(37)	97.42(10)
C(17)-N(17)-C(181)	118.3(3)	O(12)-Mo(2)-N(47)	171.18(10)
C(17)-N(17)-Mo(1)	125.0(2)	N(37)-Mo(2)-N(47)	88.85(10)
C(181)-N(17)-Mo(1)	116.7(2)	O(41)-Mo(2)-N(37)	168.58(10)

Table S13. Hydrogen bonds [Å, °] for **7**.

D-H···A	d(D-H)	d(H···A)	d(D···A)	<(DHA)
N(19)-H(19)···O(1)	0.88	2.709(5)	3.524(4)	154.6(6)
N(29)-H(29)···N(51)	0.88	2.156(15)	2.974(14)	154.5(9)
N(39)-H(39)···O(1)	0.88	2.187(17)	3.004(4)	154(3)
N(49)-H(49)···O(2)	0.88	2.182(11)	3.035(3)	163(3)

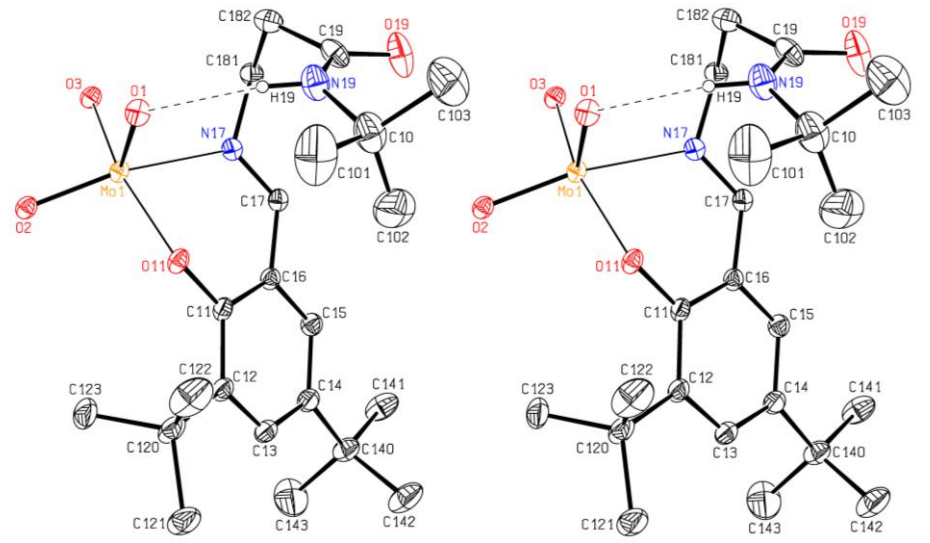

Figure S37. Stereoscopic ORTEP[6] plot of the asymmetric unit of **8** showing the atomic numbering scheme. The probability ellipsoids are drawn at the 50% probability level. H19 is drawn with an arbitrary radius, the other H atoms were omitted for clarity. The intra-molecular hydrogen bond is indicated by a dashed line.

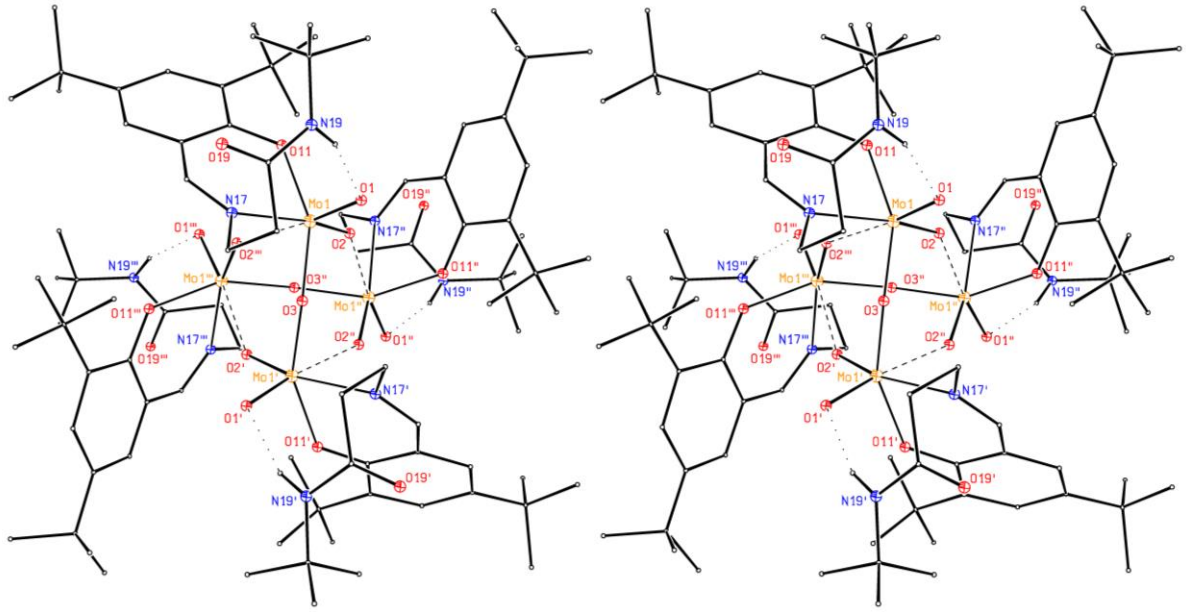

Figure S38. Stereoscopic ORTEP[6] plot of **8** in a view approx. parallel to the tetragonal c-axis. The atoms are drawn with arbitrary radii. The H atoms not involved in hydrogen bonds were omitted for clarity. The hydrogen bonds are indicated by dotted lines, the long Mo–O distances are plotted with dashed lines.

Table S14. Selected bond lengths [Å] and angles [°] for **8**.

Mo(1)-O(1)	1.7079(12)	Mo(1)-O(11)	1.9481(11)
Mo(1)-O(2)	1.7530(11)	Mo(1)-N(17)	2.2807(13)
Mo(1)-O(3)	1.9065(4)	O(11)-C(11)	1.346(2)
O(3)-Mo(1)[i)]	1.9065(4)	C(17)-N(17)	1.292(2)
Mo(1)-O(2)[iii)]	2.2500(11)	N(17)-C(181)	1.476(2)
Mo(1)-O(3)-Mo(1)[i)]	144.96(7)	C(11)-O(11)-Mo(1)	132.76(10)
Mo(1)-O(2)-Mo(1)[ii)]	142.20(6)	C(17)-N(17)-C(181)	115.97(13)
O(1)-Mo(1)-O(2)[iii)]	171.89(5)	C(17)-N(17)-Mo(1)	124.00(10)
O(2)-Mo(1)-N(17)	161.81(5)	C(181)-N(17)-Mo(1)	119.97(10)
O(3)-Mo(1)-O(11)	154.79(4)		

Symmetry transformations to generate equivalent atoms: [i)] 1-x, 1-y, z [ii)] y, 1-x, 1-z [iii)] 1-y, x, 1-z

Table S15. Hydrogen bond [Å, °] for **8**.

D-H···A	d(D-H)	d(H···A)	d(D···A)	<(DHA)
N(19)-H(19)···O(1)	0.88	2.183(4)	3.028(2)	160.8(8)

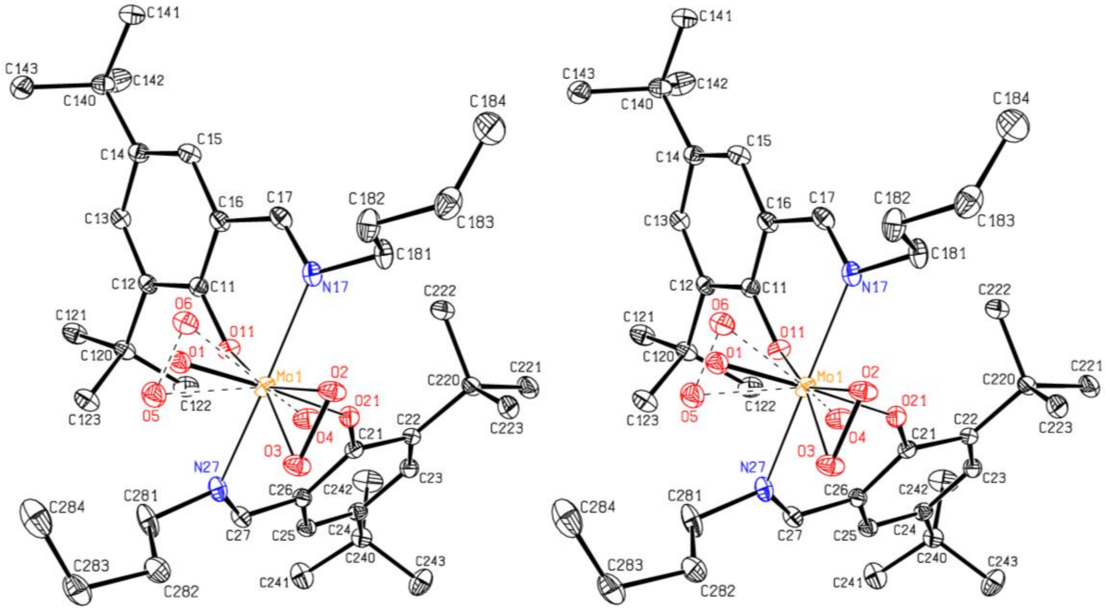

Figure S39. Stereoscopic ORTEP[6] plot of **9** showing the atomic numbering scheme. The probability ellipsoids are drawn at the 50% probability level. The bonds to atoms with site occupation factors of 0.226(5) are plotted with dashed lines. The H atoms were omitted for clarity.

Table S16. Selected bond lengths [Å] and angles [°] for **9**.

Mo(1)-O(1)	1.6817(18)	O(2)-O(3)	1.431(3)
Mo(1)-O(2)	1.9573(19)	O(11)-C(11)	1.3244(17)
Mo(1)-O(3)	1.9469(16)	C(17)-N(17)	1.2922(19)
Mo(1)-O(11)	2.0353(10)	N(17)-C(181)	1.4835(19)
Mo(1)-O(21)	2.0113(10)	O(21)-C(21)	1.3197(18)
Mo(1)-N(17)	2.1670(14)	C(27)-N(27)	1.285(2)
Mo(1)-N(27)	2.2053(14)	N(27)-C(281)	1.478(2)
N(17)-Mo(1)-N(27)	159.62(5)	C(17)-N(17)-C(181)	115.17(14)
O(11)-Mo(1)-O(21)	80.51(4)	C(17)-N(17)-Mo(1)	123.02(11)
O(1)-Mo(1)-O(21)	167.81(7)	C(181)-N(17)-Mo(1)	121.71(10)
O(2)-Mo(1)-O(11)	156.51(7)	C(21)-O(21)-Mo(1)	135.57(9)
O(3)-Mo(1)-O(11)	156.08(6)	C(27)-N(27)-C(281)	116.77(14)
O(4)-Mo(1)-O(11)	172.6(2)	C(27)-N(27)-Mo(1)	124.51(11)
C(11)-O(11)-Mo(1)	128.85(9)	C(281)-N(27)-Mo(1)	118.19(10)

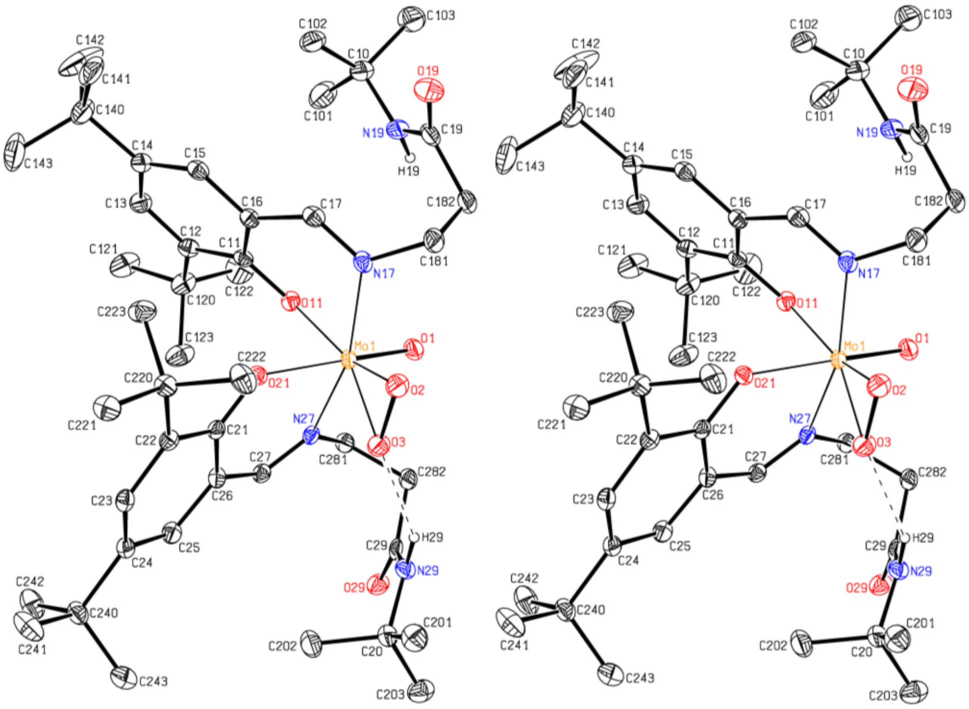

Figure S40. Stereoscopic ORTEP[6] plot of molecule A of **10** showing the atomic numbering scheme. The probability ellipsoids are drawn at the 50% probability level. The H atoms bonded to N are drawn with arbitrary radii, the other H atoms as well as the less occupied disordered *tert*-butyl group were omitted for clarity. The intra-molecular hydrogen bond is indicated by a dashed line.

Table S17. Selected bond lengths [Å] and angles [°] for **10**.

Mo(1)-O(1)	1.6959(13)	Mo(1)-N(17)	2.1542(15)	O(21)-C(21)	1.324(2)
Mo(1)-O(2)	1.9398(13)	Mo(1)-N(27)	2.1818(15)	C(27)-N(27)	1.288(2)
Mo(1)-O(3)	1.9651(14)	O(2)-O(3)	1.4426(18)	N(27)-C(281)	1.480(2)
Mo(1)-O(11)	2.0256(13)	C(17)-N(17)	1.294(2)		
Mo(1)-O(21)	2.0283(12)	N(17)-C(181)	1.480(2)		

O(1)-Mo(1)-O(21)	170.18(6)	C(17)-N(17)-C(181)	117.31(16)
O(2)-Mo(1)-O(11)	154.28(5)	C(17)-N(17)-Mo(1)	123.06(13)
O(3)-Mo(1)-O(11)	156.51(5)	C(181)-N(17)-Mo(1)	119.47(12)
O(1)-Mo(1)-O(2)	100.50(6)	C(21)-O(21)-Mo(1)	134.16(11)
O(1)-Mo(1)-O(3)	98.18(6)	C(27)-N(27)-C(281)	116.77(15)
N(17)-Mo(1)-N(27)	161.89(6)	C(27)-N(27)-Mo(1)	124.96(12)
C(11)-O(11)-Mo(1)	127.88(11)	C(281)-N(27)-Mo(1)	118.26(11)

Table S18. Hydrogen bonds [Å, °] for **10**.

D-H···A	d(D-H)	d(H···A)	d(D···A)	<(DHA)
N(19)-H(19)···O(29)i)	0.88	2.190(5)	2.998(2)	152.6(9)
N(29)-H(29)···O(3)	0.88	2.277(7)	3.0718(19)	150.1(12)
N(49)-H(49)···O(39)ii)	0.88	2.252(8)	3.060(4)	152.5(12)

Symmetry transformations used to generate equivalent atoms: i) 1-x, 1-y, 1-z ii) 2-x, 1-y, -z

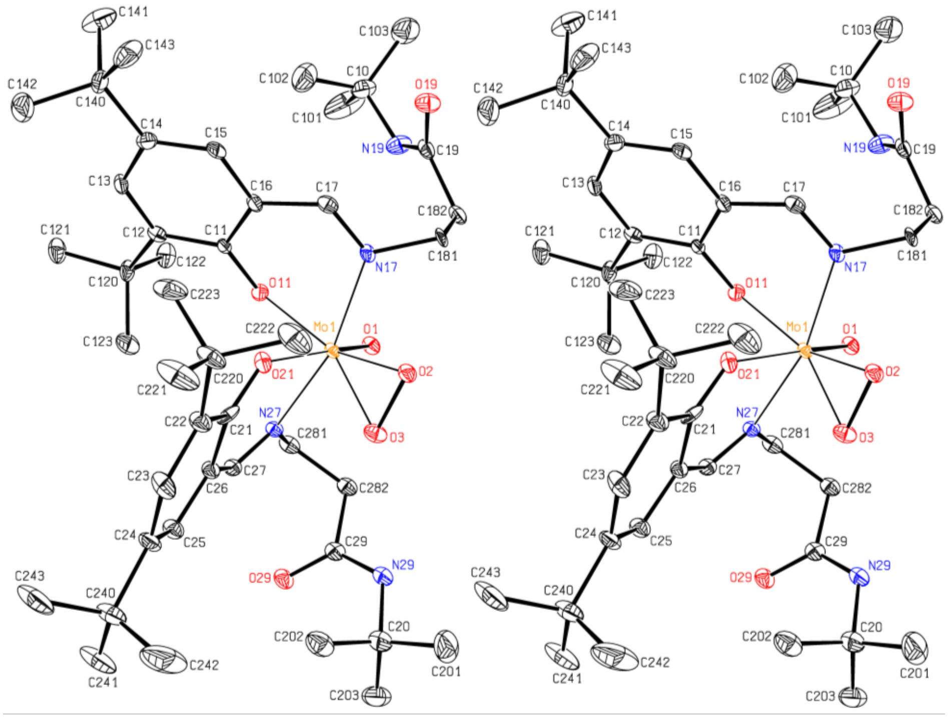

Figure S41. Stereoscopic ORTEP[6] plot of the metal complex of **10·OPMe$_3$** showing the atomic numbering scheme. The probability ellipsoids are drawn at the 50% probability level. The H atoms and trimethylphosphine oxide were omitted for clarity.

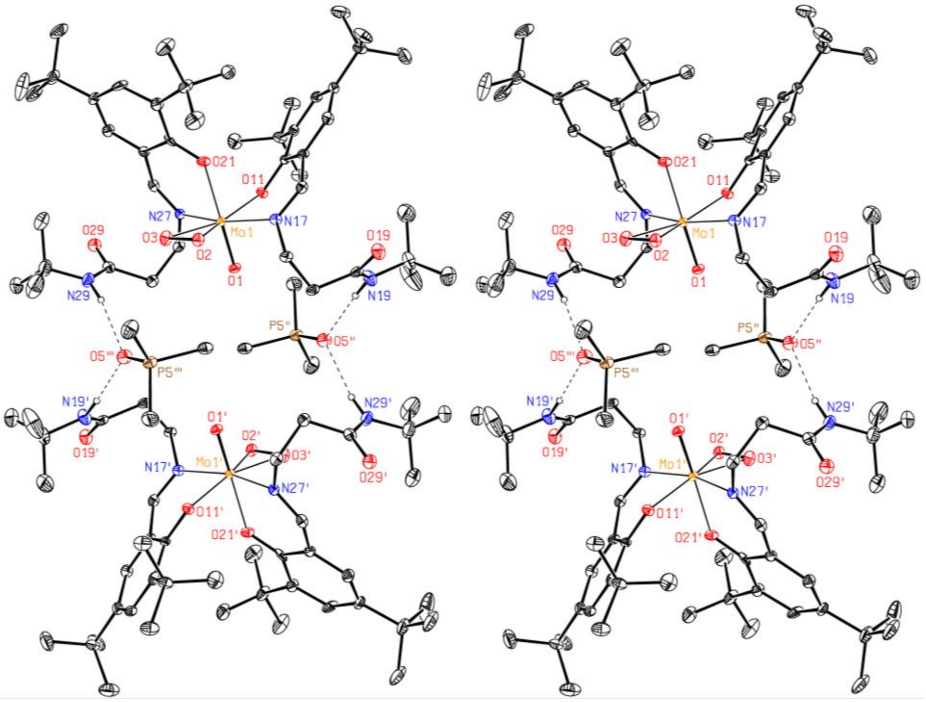

Figure S42. Stereoscopic ORTEP[6] plot of four metal complexes of **10·OPMe**$_3$ together with four molecules of trimethylphosphine oxide. The probability ellipsoids are drawn at the 50% probability level. The hydrogen bonds are indicated by dashed lines. The involved H atoms are drawn with arbitrary radii, the other H atoms were omitted for clarity.

Table S19. Selected bond lengths [Å] and angles [°] for **10·OPMe**$_3$.

Mo(1)-O(1)	1.698(2)	O(2)-O(3)	1.446(3)
Mo(1)-O(2)	1.937(3)	O(11)-C(11)	1.342(4)
Mo(1)-O(3)	1.961(3)	C(17)-N(17)	1.292(5)
Mo(1)-O(11)	2.028(3)	N(17)-C(181)	1.491(5)
Mo(1)-O(21)	2.035(3)	O(21)-C(21)	1.287(5)
Mo(1)-N(17)	2.162(3)	C(27)-N(27)	1.291(5)
Mo(1)-N(27)	2.204(3)	N(27)-C(281)	1.478(4)
O(1)-Mo(1)-O(21)	166.78(11)	C(17)-N(17)-Mo(1)	123.5(3)
O(2)-Mo(1)-O(11)	154.17(10)	C(181)-N(17)-Mo(1)	119.9(2)
O(3)-Mo(1)-O(11)	157.66(11)	C(21)-O(21)-Mo(1)	134.6(2)
N(17)-Mo(1)-N(27)	163.96(12)	C(27)-N(27)-C(281)	116.6(3)
C(11)-O(11)-Mo(1)	126.6(2)	C(27)-N(27)-Mo(1)	124.5(2)
C(17)-N(17)-C(181)	116.5(3)	C(281)-N(27)-Mo(1)	118.3(2)

Table S20. Hydrogen bonds [Å, °] for **10·OPMe$_3$**.

D-H···A	d(D-H)	d(H···A)	d(D···A)	<(DHA)
N(19)-H(19)···O(5)[i)]	0.88	2.079(5)	2.957(4)	175(3)
N(29)-H(29)···O(5)[ii)]	0.88	2.156(4)	3.027(4)	170.1(7)

Symmetry transformations used to generate equivalent atoms: [i)] x, y, z+1 [ii)] 2-x, 1-y, 1-z

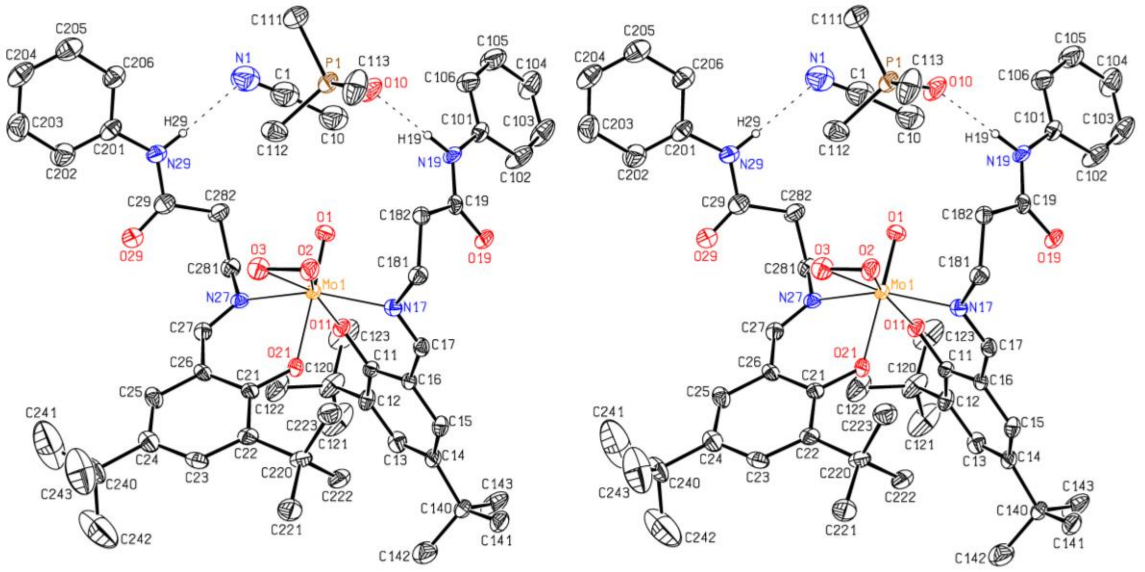

Figure S43. Stereoscopic ORTEP[6] plot of **11·OPMe$_3$** showing the atomic numbering scheme. The probability ellipsoids are drawn at the 50% probability level. The H atoms of the NH groups are drawn with arbitrary radii, the other H atoms as well as the disordered acetonitrile solvate molecules were omitted for clarity. The hydrogen bonds are indicated by dashed lines.

Table S21. Selected bond lengths [Å] and angles [°] for **11·OPMe$_3$**.

Mo(1)-O(1)	1.698(3)	Mo(1)-O(21)	2.036(3)
Mo(1)-O(2)	1.961(4)	Mo(1)-N(17)	2.167(4)
Mo(1)-O(3)	1.945(3)	Mo(1)-N(27)	2.189(4)
Mo(1)-O(11)	2.014(3)	O(2)-O(3)	1.406(4)
O(1)-Mo(1)-O(21)	170.75(15)	C(17)-N(17)-Mo(1)	123.2(3)
O(3)-Mo(1)-O(11)	156.69(13)	C(181)-N(17)-Mo(1)	120.8(3)
O(2)-Mo(1)-O(11)	156.74(12)	C(21)-O(21)-Mo(1)	132.0(3)
N(17)-Mo(1)-N(27)	161.32(13)	C(27)-N(27)-C(281)	116.7(4)
C(11)-O(11)-Mo(1)	131.1(3)	C(27)-N(27)-Mo(1)	124.1(3)
C(17)-N(17)-C(181)	115.7(4)	C(281)-N(27)-Mo(1)	119.1(3)

Table S22. Hydrogen bonds [Å, °] for **11·OPMe$_3$**.

D-H···A	d(D-H)	d(H···A)	d(D···A)	<(DHA)
N(19)-H(19)···O(10)	0.88	1.918(4)	2.795(4)	174.0(11)
N(29)-H(29)···N(1)	0.88	2.240(6)	3.117(5)	175(2)

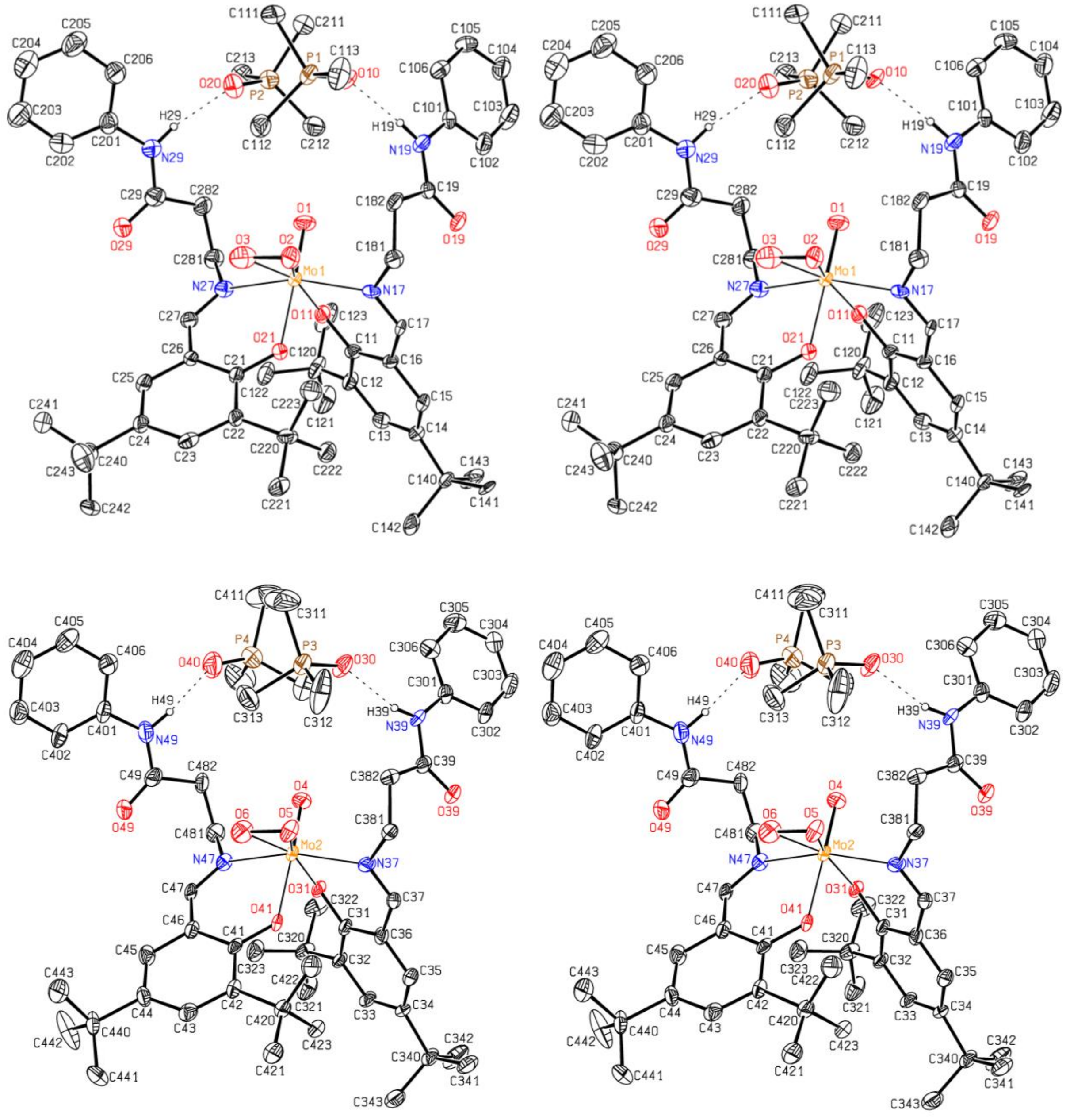

Figure S44. Stereoscopic ORTEP[6] plot of complex A (above) and of complex B (below) of **11·2OPMe$_3$** showing the atomic numbering schemes. The probability ellipsoids are drawn at the 50% probability level. The H atoms of the NH groups are drawn with arbitrary radii, the other H atoms were omitted for clarity. The hydrogen bonds are indicated by dashed lines.

Table S23. Selected bond lengths [Å] and angles [°] for **11·2OPMe$_3$**.

Mo(1)-O(1)	1.693(3)	Mo(1)-O(21)	2.028(3)
Mo(1)-O(2)	1.929(3)	Mo(1)-N(17)	2.164(4)
Mo(1)-O(3)	1.943(3)	Mo(1)-N(27)	2.191(4)
Mo(1)-O(11)	2.001(3)	O(2)-O(3)	1.360(5)
O(1)-Mo(1)-O(21)	171.03(14)	C(17)-N(17)-Mo(1)	125.1(3)
O(2)-Mo(1)-O(11)	156.64(14)	C(181)-N(17)-Mo(1)	120.2(2)
O(3)-Mo(1)-O(11)	157.20(13)	C(21)-O(21)-Mo(1)	132.1(2)
N(17)-Mo(1)-N(27)	162.58(12)	C(27)-N(27)-C(281)	116.2(4)
C(11)-O(11)-Mo(1)	132.8(3)	C(27)-N(27)-Mo(1)	124.3(3)
C(17)-N(17)-C(181)	114.4(4)	C(281)-N(27)-Mo(1)	119.5(3)

Table S24. Hydrogen bonds [Å, °] for **11·2OPMe$_3$**.

D-H···A	d(D-H)	d(H···A)	d(D···A)	<(DHA)
N(19)-H(19)···O(10)	0.88	1.890(4)	2.766(4)	173.5(13)
N(29)-H(29)···O(20)	0.88	1.977(5)	2.836(4)	164.9(11)
N(39)-H(39)···O(30)	0.88	1.940(14)	2.782(4)	160(4)
N(49)-H(49)···O(40)	0.88	1.945(9)	2.790(4)	161(2)

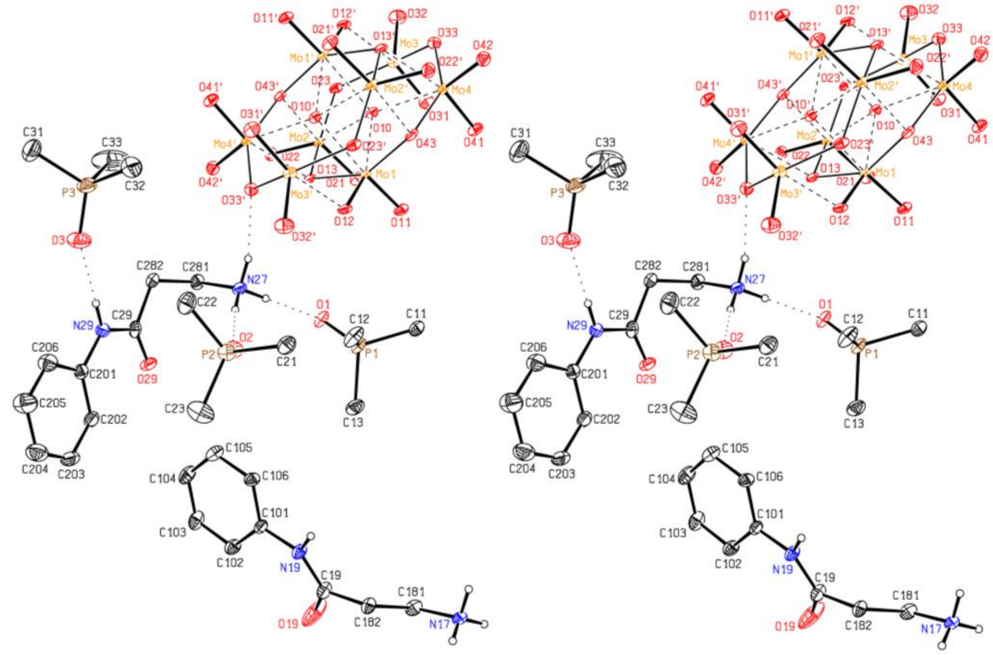

Figure S45. Stereoscopic ORTEP[6] plot of **12** showing the atomic numbering scheme. The probability ellipsoids are drawn at the 50% probability level. The H atoms of the NH groups and those of the NH_3 groups are drawn with arbitrary radii, the other H atoms were omitted for clarity. The hydrogen bonds are indicated by dotted lines. The Mo···O bonds in the range 2.169(3)-2.349(3) Å were plotted with dashed lines.

Table S25. Selected bond lengths [Å] and angles [°] for **12**.

Mo(1)-O(11)	1.693(3)	Mo(3)-O(31)	1.707(3)
Mo(1)-O(12)	1.745(3)	Mo(3)-O(32)	1.700(3)
Mo(1)-O(13)	1.947(3)	Mo(3)-O(33)	1.927(3)
O(13)-Mo(2)	2.011(3)	O(33)-Mo(4)	1.925(3)
Mo(2)-O(21)	1.720(3)	Mo(4)-O(41)	1.698(3)
Mo(2)-O(22)	1.695(3)	Mo(4)-O(42)	1.708(3)
Mo(2)-O(23)	1.875(3)	Mo(4)-O(43)	2.003(3)
O(23)-Mo(3)	1.936(3)	O(43)-Mo(1)	1.954(3)

Mo(1)-O(11)	1.693(3)	Mo(3)-O(31)	1.707(3)
Mo(1)-O(12)	1.745(3)	Mo(3)-O(32)	1.700(3)
Mo(1)-O(13)	1.947(3)	Mo(3)-O(33)	1.927(3)
O(13)-Mo(2)	2.011(3)	O(33)-Mo(4)	1.925(3)
Mo(2)-O(21)	1.720(3)	Mo(4)-O(41)	1.698(3)
Mo(2)-O(22)	1.695(3)	Mo(4)-O(42)	1.708(3)
Mo(2)-O(23)	1.875(3)	Mo(4)-O(43)	2.003(3)
O(23)-Mo(3)	1.936(3)	O(43)-Mo(1)	1.954(3)

Symmetry transformation used to generate equivalent atoms: [i] 1-x, 2-y, 1-z

Table S26. Hydrogen bonds [Å, °] for **12**.

D-H···A	d(D-H)	d(H···A)	d(D···A)	<(DHA)
N(17)-H(171)···O(1)ii)	0.91	1.831(4)	2.737(4)	174.1(15)
N(17)-H(172)···O(11)ii)	0.91	2.563(7)	3.286(4)	136.8(7)
N(17)-H(172)···O(21)ii)	0.91	2.150(6)	2.915(4)	141.2(6)
N(17)-H(173)···O(21)iii)	0.91	2.037(7)	2.849(4)	148.0(10)
N(19)-H(19)···O(22)ii)	0.88	2.40(2)	2.935(5)	119.6(18)
N(19)-H(19)···O(42)iv)	0.88	2.122(9)	2.966(4)	161(2)
N(27)-H(271)···O(1)	0.91	1.920(4)	2.828(4)	175.8(10)
N(27)-H(272)···O(2)	0.91	1.786(6)	2.692(4)	173(3)
N(27)-H(273)···O(33)i)	0.91	1.965(4)	2.855(4)	165.2(8)
N(29)-H(29)···O(3)	0.88	1.940(5)	2.817(4)	174.0(17)

Symmetry transformations used to generate equivalent atoms:

i) 1-x, 2-y, 1-z   ii) x, y-1, z   iii) -x, 1-y, 1-z   iv) 1-x, 1-y, 1-z

References

[1] N. Zwettler, A. Dupé, J. A. Schachner, F. Belaj, N. C. Mösch-Zanetti, *Inorg. Chem.* **2015**, *54*, 11969–11976.

[2] E. Safaei, M. M. Kabir, A. Wojtczak, Z. Jagličić, A. Kozakiewicz, Y.-I. Lee, *Inorg. Chim. Acta* **2011**, *366*, 275–282.

[3] M. E. Judmaier, C. Holzer, M. Volpe, N. C. Mösch-Zanetti, *Inorg. Chem.* **2012**, *51*, 9956–9966.

[4] G. M. Sheldrick, *Acta Crystallogr, A* **2008**, *64*, 112–122.

[5] G. M. Sheldrick, *Acta Crystallogr, C* **2015**, *71*, 3–8.

[6] C. K. Johnson, ORTEP. Report ORNL-3794. Oak Ridge National Laboratory, Tennessee, USA (1965).
